# Host–gut microbiota interactions shape parasite infections in farmed Atlantic salmon

**DOI:** 10.1128/msystems.01043-23

**Published:** 2024-01-31

**Authors:** Jaelle C. Brealey, Miyako Kodama, Jacob A. Rasmussen, Søren B. Hansen, Luisa Santos-Bay, Laurène A. Lecaudey, Martin Hansen, Even Fjære, Lene S. Myrmel, Lise Madsen, Annette Bernhard, Harald Sveier, Karsten Kristiansen, M. Thomas P. Gilbert, Michael D. Martin, Morten T. Limborg

**Affiliations:** 1 Department of Natural History, NTNU University Museum, Norwegian University of Science and Technology (NTNU), Trondheim, Norway; 2 Center for Evolutionary Hologenomics, Globe Institute, Faculty of Health and Medical Sciences,University of Copenhagen, Copenhagen, Denmark; 3 Department of Biology, Laboratory of Genomics and Molecular Biomedicine, University of Copenhagen, Copenhagen, Denmark; 4 Aquaculture Department, SINTEF Ocean, Trondheim, Norway; 5 Department of Environmental Science, Environmental Metabolomics Lab, Aarhus University, Roskilde, Denmark; 6 Institute of Marine Research, Bergen, Norway; 7 Department of Clinical Medicine, University of Bergen, Norway, Bergen, Norway; 8 Lerøy Seafood Group ASA, Bergen, Norway; The University of Maine, Orono, Maine, USA

**Keywords:** holobiont, hologenome, microbiome, metagenomics, multi-omics, salmonid, aquaculture

## Abstract

Animals and their associated microbiota share long evolutionary histories. However, it is not always clear how host genotype and microbiota interact to affect phenotype. We applied a hologenomic approach to explore how host–microbiota interactions shape lifetime growth and parasite infection in farmed Atlantic salmon (*Salmo salar*). Multi-omics data sets were generated from the guts of 460 salmon, 82% of which were naturally infected with an intestinal cestode. A single *Mycoplasma* bacterial strain, MAG01, dominated the gut metagenome of large, non-parasitized fish, consistent with previous studies showing high levels of *Mycoplasma* in the gut microbiota of healthy salmon. While small and/or parasitized salmon also had high abundance of MAG01, we observed increased alpha diversity in these individuals, driven by increased frequency of low-abundance Vibrionaceae and other *Mycoplasma* species that carried known virulence genes. Colonization by one of these cestode-associated *Mycoplasma* strains was associated with host individual genomic variation in long non-coding RNAs. Integrating the multi-omic data sets revealed coordinated changes in the salmon gut mRNA transcriptome and metabolome that correlated with shifts in the microbiota of smaller, parasitized fish. Our results suggest that the gut microbiota of small and/or parasitized fish is in a state of dysbiosis that partly depends on the host genotype, highlighting the value of using a hologenomic approach to incorporate the microbiota into the study of host–parasite dynamics.

**IMPORTANCE:**

Studying host–microbiota interactions through the perspective of the hologenome is gaining interest across all life sciences. Intestinal parasite infections are a huge burden on human and animal health; however, there are few studies investigating the role of the hologenome during parasite infections. We address this gap in the largest multi-omics fish microbiota study to date using natural cestode infection of farmed Atlantic salmon. We find a clear association between cestode infection, salmon lifetime growth, and perturbation of the salmon gut microbiota. Furthermore, we provide the first evidence that the genetic background of the host may partly determine how the gut microbiota changes during parasite-associated dysbiosis. Our study therefore highlights the value of a hologenomic approach for gaining a more in-depth understanding of parasitism.

## INTRODUCTION

There is constant interaction between the vertebrate host and its gut microbiota via the host’s immune system (1, 2). The microbiota trains and matures the host immune system (3), while the host immune system keeps the microbiota under control to maintain important symbiotic functions, such as providing the host with key nutrients from microbial metabolism of host dietary components (4). On one hand, the host genetic background likely affects gut microbiota composition (5–8). On the other hand, the gut microbiota has been found to broadly modulate host gene expression by triggering changes in transcription factor binding and chromatin assembly (9). Indeed, many studies have demonstrated links between an altered gut microbiota and host disease states, such as infections and inflammatory diseases (reviewed in references 1, 2). Such disruptions have been referred to as dysbiosis, defined as any change to the resident commensal gut microbiota relative to the community found in healthy individuals (10).

Host–microbiota interactions in the gut are particularly relevant to understanding infections by intestinal parasites like helminths (worms), which directly interact with the host immune system and directly or indirectly influence the host–gut microbiota (11). Gut dysbiosis is frequently associated with helminth infections (12–14), and in some systems, the gut microbiota composition directly promotes or inhibits helminth colonization or reproduction (15–17). Furthermore, some parasitic helminths have been shown to carry an internal microbiota (13, 18–20), including essential endosymbionts (21). Helminths and other parasites may also act as vectors, introducing novel, potentially pathogenic microbes to the host animal (22–24).

The complex interactions among host genotype, host immune system, and host microbiota have led to the concept of a “holobiont,” which considers the host organism and its associated microbiota as a single co-functioning scaffold of organisms (25). The holobiont has a hologenome consisting of the host genome and the genomes of all its microbiota (the metagenome) (26). Thus, it has been argued that the host–gut microbiota has to be studied in the context of the host genotype and gene expression landscape to fully understand complex phenotypes, such as lifetime growth or disease susceptibility (10). Such hologenomic approaches use multi-omic data sets from both the host (e.g., genomic and transcriptomic) and the microbiota (e.g., metagenomic) to untangle host–microbiota interactions and their associations with phenotypes of interest (27).

We explored how host–microbiota interactions shape parasite infections and lifetime growth in a commercial cohort of Atlantic salmon (*Salmo salar*). Production animals often provide excellent systems for hologenomic research because production cohorts are raised under controlled environmental conditions and have well defined phenotypes of interest (27, 28). Previous research in Atlantic salmon has primarily focused on the genomic variation of the salmon host when aiming to explain differences in phenotypes of relevance to production, such as lifetime growth (29, 30). In light of the growing body of holobiont research, we hypothesized that part of the observed variation in growth and parasite prevalence of farmed Atlantic salmon could be explained by variation in the salmon gut microbiota or vice versa (28). Thus, our overall objective was to characterize the gut environment (metagenome, metabolome, and host transcriptome) and underlying genomic variability of 460 farmed Atlantic salmon exhibiting extensive variance in both lifetime growth and parasite infection levels.

The study salmon population was housed in open seawater pens, allowing natural infection with cestodes (or tapeworms) of the genus *Eubothrium*. This cestode colonizes the intestine of the definite salmon host, impeding the salmon’s growth or even causing its death in extreme cases (31, 32). We aimed to apply a hologenomic framework to this system to investigate (i) how the gut environment differed among salmon of different size classes and parasitism levels and (ii) whether there was evidence for host genetic control of the salmon gut microbiota.

## RESULTS

### Summary of data sets

**Fig 1:**
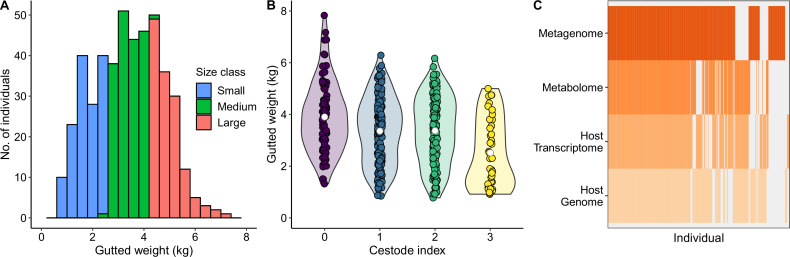
Overview of fish metadata and -omic data sets. (**A**) Distribution of lifetime growth of the 460 sampled salmon, measured as gutted weight (kg) at harvest. Individuals were classified into three discrete size classes as indicated by the colored bars, with the aim to have approximately equal numbers of individuals in each class. (**B**) Gutted weight at harvest decreases as cestode infection levels increase. Violin plots show the distribution of salmon weights; white circles indicate the mean. (**C**) Gut metagenomes, gut metabolomes, host transcriptomes, and host genomes were successfully generated for a subset of individuals. Gray bars indicate that an -omic data set is missing for that individual. Individuals are ordered by the number of -omic data sets with data available.

Under our experimental design, we aimed to sample equal numbers of Atlantic salmon across the entire size distribution of a commercial cohort at harvest (gutted weight range: 0.78–7.83 kg), resulting in 140 fish classified as “small” (gutted weight ca. <2.6 kg), 139 “large” fish (gutted weight ca. >4.2 kg), and 181 “medium” fish (Fig. 1A). Salmon were sourced from two sea pens on the same aquaculture farm in southwest Norway fed two different commercial diets (pen1/feed1 = 250 individuals and pen2/feed2 = 210 individuals). Of these 460 individuals, 375 (81.5%) were parasitized by at least one intestinal cestode. We used a semiquantitative cestode index as a measure of cestode infection load. There was strong evidence that salmon lifetime growth (gutted weight at harvest) decreased as cestode infection levels increased (Fig. 1B; *P* < 0.001, *R*^2^ = 0.057).

After filtering for data quality (see Materials and Methods), metagenomes were generated from gut content samples for 392 individuals. Host genomes were recovered from gill tissue for 361 individuals (coverage average: 4.7×, range: 0.0003–17.3×, 998,475 sites after filtering). Transcriptomes from salmon gut epithelial tissue were generated using mRNA sequencing for 343 individuals, resulting in expression data for 19,500 transcripts after processing. Metabolomes, including both salmon and microbial metabolites, were generated from gut content samples for 334 individuals, resulting in an inventory of 969 metabolites, 764 of which could be annotated at the superclass level. A total of 208 individuals were represented in all four -omic data sets (Fig. 1C). We therefore aimed to use statistical methods that can accommodate missing data, where possible. We also generated fatty acid profiles from salmon muscle tissue for 412 individuals to assess any potential link between microbiota functions and this key production trait for salmon.

We included salmon from two sea pens/commercial diets (hereafter referred to as “feed types”) to act as pseudo-replicates, to identify robust associations among different -omic levels consistent between pen environments, rather than to evaluate the effect of diet as an explanatory variable. Indeed, cestode detection was similar between feed1 and feed2 (84.4% and 78.1%, respectively; *P* = 0.092), confirming that pen environment was not strongly affecting salmon parasitism levels. However, we observed a generally small but significant effect of feed type at all -omic levels (Fig. S1–S4). The composition of fatty acids in salmon muscle was strongly affected by feed type (Fig. S4), explaining 34.8% of the variation in this data set (*P* = 0.001, Table S1) and confirming that feed has a large effect on the fatty acid profile of salmon fillets. At the other -omic levels, feed type explained 1%–3% of the variation in the data sets (*P* = 0.001, Table S1). We therefore treated feed type as a confounding covariate and control for its effect in all subsequent statistical analyses.

### Cestode infection is associated with an altered gut microbiota

**Fig 2:**
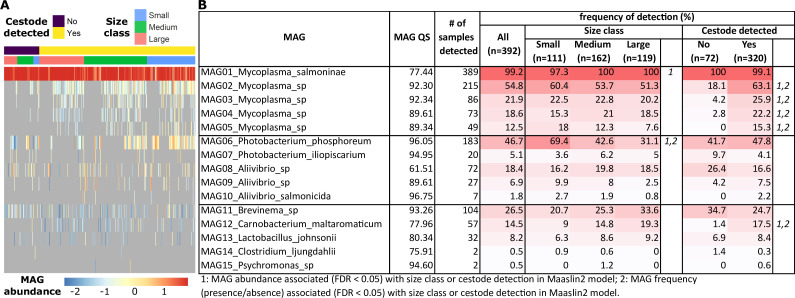
MAG abundance and frequency of detection in the Atlantic salmon gut metagenome. (**A**) Normalized MAG abundances per sample (log-transformed). Samples are ordered and annotated by cestode detection and size class, while MAGs are ordered by taxonomy and frequency of detection. MAGs that were not detected in a sample are shown in gray. (**B**) MAG detection in all samples, separated by size class or cestode detection. MAGs with more frequent detection (percentage of samples) are colored in a darker red. MAG quality score (QS) indicates the quality score of the MAG based on completeness and redundancy scores (values closer to 100 are better). MAG abundance or detection frequency that was associated (FDR < 0.05) with size class or cestode detection is indicated by 1 or *2*, respectively. Results from the Maaslin2 models with FDR of <0.2 are presented in Table S4.

The intestinal microbiota community structure was characterized by low diversity, with only 15 unique bacterial metagenome-assembled genomes (MAGs) recovered from all 392 metagenomic samples (Table S2). Most individuals were dominated by MAG01, *Mycoplasma salmoninae* (Fig. 2A), a species known to be positively associated with a healthy and undisturbed gut microbiota in salmonids (19, 33–39). However, some variation in the microbiota composition was observed in small individuals and those infected with cestodes (Fig. S1; Table S1). Alpha diversity was higher in small and/or parasitized fish (Fig. S5; Table S3, *P* < 0.001 and *P* = 0.014, respectively). The abundance of MAG01 *M. salmoninae* decreased in small fish (Fig. 2A; Table S4 false discovery rate [FDR] = 0.0007). Four other *Mycoplasma* species (MAG02-MAG05) were strongly associated with cestode presence (Fig. 2B; Table S4; FDR < 0.02 for all). Two of these species, MAG02 and MAG03, have been previously associated with the internal cestode microbiota (19) (Table S2). MAG12 *Carnobacterium maltaromaticum* was also associated with cestode presence (Table S4, FDR < 0.05). MAG06 *Photobacterium phosphoreum* was at higher frequency and higher abundance in small fish (Fig. 2; Table S4; FDR < 0.001). Overall, we observed a shift in the gut microbiota, characterized by decreased abundance of commensal *M. salmoninae* and increased detection of other taxa in small, parasitized salmon compared to larger cestode-free fish.

In general, the gut microbiota of the salmon was similar in both feed types (Fig. S1; Table S1). However, MAG08 *Aliivibrio* was associated with increased abundance and frequency in feed2 compared to feed1 (Table S4, FDR < 0.01).

We confirmed that the 15 MAGs detected in the salmon gut metagenomes captured the majority of diversity in the gut content at the genus level via 16S metabarcoding of a subset of gut content samples (Fig. S6). We also compared the gut content samples with paired gut mucosa scrapes and feed pellet samples (Fig. S7). The most abundant taxa, namely, *Mycoplasma*, *Photobacterium*, *Aliivibrio*, and *Brevinema*, were detected in both gut content and gut mucosa samples. The only genus detected at high abundance in the feed pellet samples was *Lactobacillus*, which was also detected at low abundance in the gut content samples (Fig. S7). We therefore conclude that while MAG13 *Lactobacillus johnsonii* may be derived from the feed pellets, the other most abundant MAGs (MAG01–MAG11) are likely true residents of the core salmon gut microbiota.

### Host genomic variation is associated with microbiota composition

**Fig 3:**
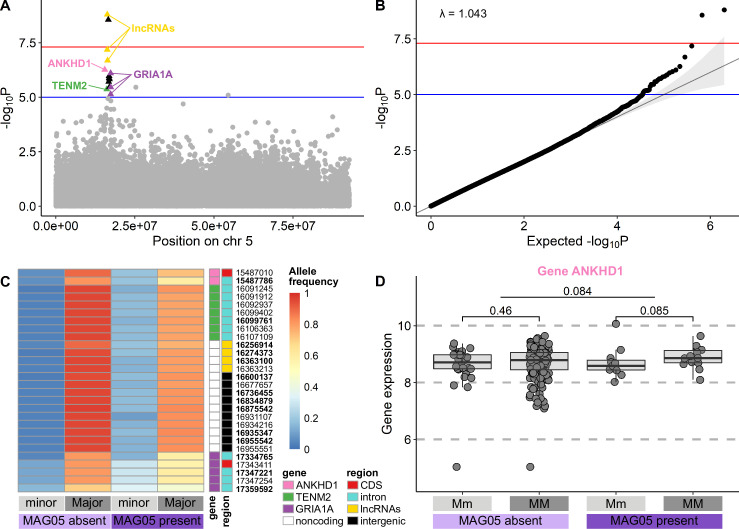
MAG05 *Mycoplasma* GWAS results. (**A**) SNP peak observed on chromosome 5 for the GWAS association with MAG05 presence/absence. *P* values have been −log10 transformed so that higher values are more significant. Moderately (*P* < 1e−5, blue line) and highly (*P* < 5e−8, red line) associated SNPs on the peak are highlighted in triangles against the background gray circles of chromosome 5. SNPs falling within annotated transcripts in this peak are colored and annotated by their gene symbol, where relevant. SNPs falling within intergenic regions are shown as black triangles. (**B**) QQ plot and genomic inflation factor (λ) for the GWAS association with MAG05 presence/absence. (**C**) Minor and major allele frequencies for the 28 associated (bold face) and linked (plain face) SNPs. Frequencies have been calculated separately for individuals with MAG05 present or absent. SNPs are annotated by gene symbol (where relevant, same as in panel A) and genomic region (CDS). (**D**) Gene expression (normalized and scaled counts) of *ankhd1*, the only MAG05-associated gene for which comparable transcriptomic data were available. Samples have been divided by MAG05 presence/absence and genotype at SNP 15487786 (Mm or MM; minor/minor individuals were excluded due to low sample numbers). *P* values are shown for Mm vs MM and MAG05 absent vs present gene expression comparisons using the Wilcoxon rank-sum test. *ANKHD1*, ankyrin repeat and KH domain-containing protein 1-like; CDS, coding sequence; *GRIA1A*, glutamate receptor 1-like; lncRNA, long non-coding RNA; Mm, major/minor; MM, major/major; *TENM2*, teneurin-2-like.

We investigated associations between the underlying genomic variability of the salmon host and its gut microbiota composition with a series of genome-wide association studies (GWAS) using both MAG abundance and presence/absence as phenotypes. Cestode detection, size class, and feed type were included as covariates. Generally, little host genetic variation was associated with microbiota composition (Fig. S8; Table S5). However, there was one 1.8-Mbp region on chromosome 5 associated with the detection of MAG05 *Mycoplasma* (Fig. 3A and B), with two single nucleotide polymorphisms (SNPs) strongly and 12 SNPs moderately associated with MAG05 detection (*P* < 5e−8 and *P* < 1e−5, respectively; Table S6). Through linkage disequilibrium analysis, we identified 14 additional SNPs linked to these sites. Of these 28 SNPs, 12 SNPs were located within introns of three coding genes; 2 SNPs were found in coding regions in two of these genes that resulted in synonymous variants; 4 SNPs were linked to long non-coding RNAs; and 10 SNPs fell in intergenic regions (Fig. 3C; Table S7). MAG05 was detected more frequently in individuals carrying at least one copy of the minor allele at these sites (Fig. 3C). While MAG05 detection was also associated with cestode presence (Fig. 2B), cestode presence or absence was not associated with the same host genomic region in the GWAS (Fig. S8).

Expression of only one of the three coding genes (*ankhd1*-like) could be quantified in the mRNA transcriptome data set, likely due to high levels of RNA degradation in this data set (Data S1). There was weak evidence that *ankhd1*-like had increased expression in individuals with the major/major genotype compared to the major/minor genotype (*P* = 0.084; Fig. 3D; only one individual was homozygous for the minor allele; thus, this individual/genotype was excluded from statistical analysis). This weak association remained in individuals where MAG05 was present, but the trend was not observed in MAG05-negative individuals (Fig. 3D). Gene expression analyses of a 1-Mbp region surrounding the chromosome 5 peak in the transcriptomic data did not reveal any strong patterns of differentially expressed genes associated with MAG05 detection or genotype of the 14 SNPs (Table S8).

### Multi-omics reveals joint host–microbiota changes in salmon gut environment

We performed multi-omics factor analysis (MOFA) to identify coordinated changes among the salmon gut metabolome, transcriptome, and metagenome and correlated these changes with cestode detection and size class variables. MOFA is analogous to principal component analysis (PCA), where matrices of -omic data from the same individuals are reduced to a small number of latent factors representing the key contributors of variation across a multi-omic data set (40). Like PCA, factors are ordered by the amount of variance explained (i.e., factor 1 explains the most variance). In our MOFA model, we included the 500 most variable features in the metabolome and transcriptome data sets. For the metagenome data set, we included MAG presence/absence (excluding MAGs detected in <10 samples), detection of any low-abundant *Mycoplasma* MAG (i.e., MAG02–MAG05), detection of any *Photobacterium* MAG (MAG06–MAG07), detection of any *Aliivibrio* MAG (MAG08–MAG10), and detection of samples with “high” vs “low” abundance of *M. salmoninae* (MAG01; see Materials and Methods for details). The model was trained with 15 latent factors. To account for the potential confounding effect of feed type, we also repeated the MOFA within each feed type separately. We focus here on consistent results across the three models, i.e., the total data set, feed1, or feed2 only (Data S2 through S4).

Overall, the MOFA models explained 65%–71% of the total variance in the transcriptomic data set, 48%–49% in the metabolomic data set, and 13%–22% in the metagenomic data set (Table S9). Generally, each factor in each model explained substantial variation (> 1%) in only one -omic data set (Table S9). However, we identified several factors in each model that explained variation connecting at least two -omic data sets.

**Fig 4:**
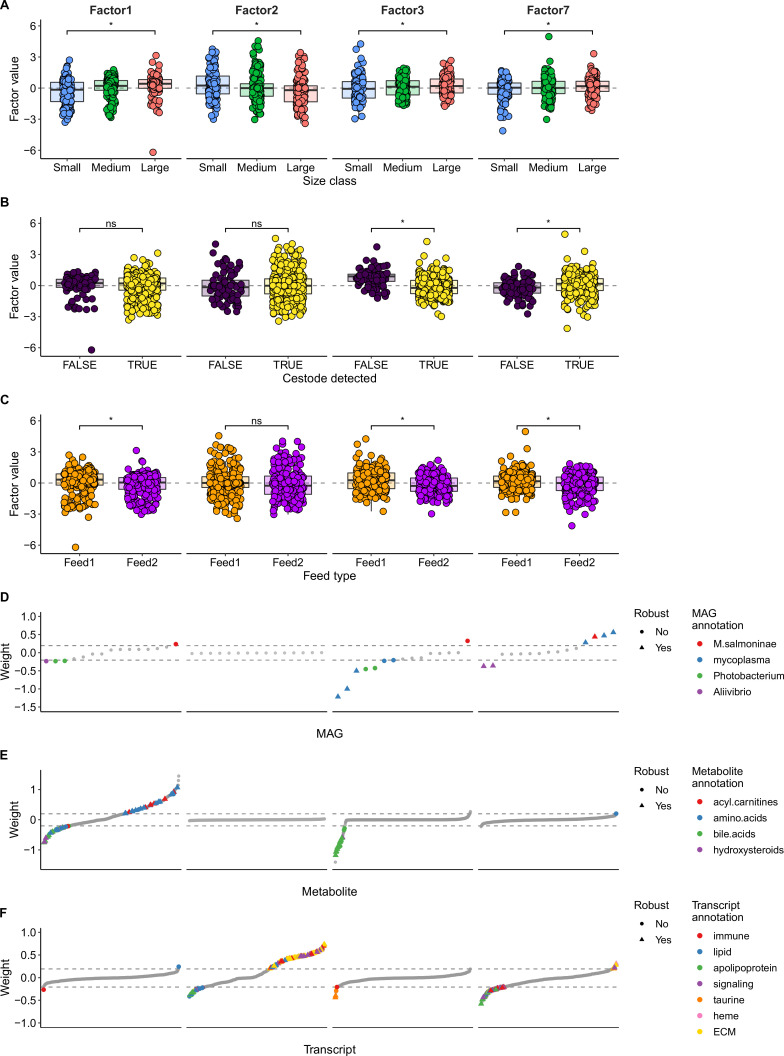
Multi-omics factor analysis (MOFA) results for factors 1, 2, 3, and 7 in the full model (i.e., both feed types combined). (**A**) All four factors were correlated with size class. (**B**) Factors 3 and 7 were correlated with cestode detection. (**C**) Factors 1, 3, and 7 were correlated with feed type. In panels A–C, * indicates adjusted *P* < 0.05; ns indicates adjusted *P* ≥ 0.05. (D–F) Feature weights for the metagenome (**D**), metabolome (**E**), and transcriptome (**F**) for factors 1, 2, 3, and 7. Features are ranked according to their weight. The higher the absolute weight, the more strongly associated a feature is with that factor. A positive weight indicates the feature has higher levels in samples with positive factor values, while a negative weight indicates the opposite. Features with weights of >0.2 or <−0.2 are colored by MAG species or genus (**D**) or most frequent functional annotation (**E and F**), while features with less frequent annotations or those with weights between −0.2 and 0.2 (as indicated by the dashed lines) are shown in gray. Features that were consistently found between the two feed type MOFA models to have similar patterns as in the full model (i.e., found in factors with similar fish phenotype correlations and similar metagenome composition patterns) are labeled as “robust.” ECM: extracellular matrix.

The first two factors in each model captured changes generally correlated with size class in either the metabolome or the transcriptome (Fig. 4; Fig. S9 and S10). These changes were usually consistent between feed types. Metabolites associated with large fish included amino acids, peptides, acyl carnitines, fatty acids, and fatty acid esters, whereas metabolites associated with small fish included bile acids, hydroxysteroids, and derivatives. Gene expression associated with large fish was inconsistent between feed types; however, genes with increased expression in small fish included those with functions in cytoskeleton signaling (e.g., actin and tensin) and immune processes (e.g., the immune modulator thrombospondin-1).

One factor in each model captured correlated changes in the metagenome and metabolome (factor 3 in the combined model in Fig. 4, factor 3 in the feed1 model in Fig. S9, and factor 4 in the feed2 model in Fig. S10). This factor was correlated with cestode detection (Fig. 4B). Consistent with previous observations, low abundance of mycoplasma MAGs showed increased detection in parasitized fish, while a high abundance of MAG01 *M. salmoninae* tended to be associated with increased detection in non-parasitized fish (Fig. 4D). Several metabolites showing an increased abundance in parasitized fish were annotated as bile acids, alcohols, and derivatives (factor 3 in Fig. 4E). While this factor did not substantially contribute to the transcriptome data set, two genes with core roles in the biosynthesis of taurine, a key component of taurinated bile acids, had consistently higher expression in parasitized fish (Factor three in Fig. 4F).

We also observed several factors that captured changes generally correlated with size class in the metagenome and transcriptome (factor 7 in Fig. 4). However, these changes were partially dependent on feed type; while MAG01 was associated with large fish in all three models, within feed1, MAG06 *P. phosphoreum* was associated with small fish (factors 6 and 7 in Fig. S9), while within feed2, MAG08 *Aliivibrio* tended to be associated with small fish (factor 5 in Fig. S10). Generally, antimicrobial peptides, like ladderlectin and cathelicidin, and lipid-binding apolipoproteins had increased expression in small fish, while collagen had increased gene expression in large fish (factors 6 and 7 in Fig. S9 and factors 5 and 6 in Fig. S10).

### Phylogenetic and functional characterization of cestode- and size-associated MAGs

**Fig 5:**
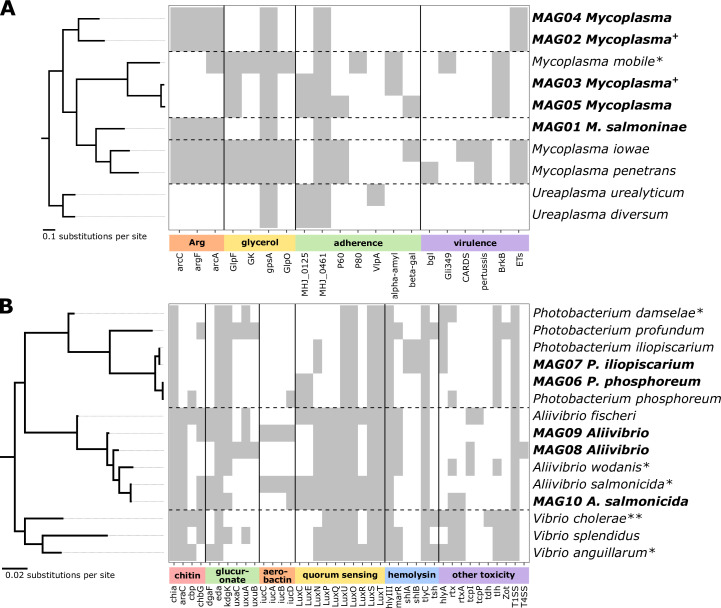
Phylogenetic and functional analysis of Mycoplasmataceae (**A**) and Vibrionaceae (**B**) MAGs compared to reference genomes. Genomes are ordered by their phylogenetic relationships, from a tree built from single-copy bacterial genes present in all genomes. Trees were rooted using *Ureaplasma* (**A**) and *Vibrio* (**B**) reference genomes as outgroups. Branch lengths are measured in substitutions per site. MAGs from this study are highlighted in bold. Heatmap presents the presence (gray) or absence (white) of genes involved in nutrient metabolism, colonization, and/or virulence functions. Genes are grouped by related functions. In panel A, these are genes in the arginine deiminase pathway (“arg”), glycerol uptake and metabolism (“glycerol”), adherins that bind to host cell molecules and enzymes which cleave host cell polysaccharides to aid adherence (“adherence”), and more specific virulence factors, including the *M. mobile* gliding mechanism Gli349 and bacterial toxin production (“virulence”). Panel B shows genes of known relevance to the Vibrionaceae clade, specifically genes involved in chitin degradation, involved in the colonization of invertebrates (“chitin”), the D-glucuronate degradation pathway, involved in adaptation to the gut (“glucuronate”), synthesis of aerobactin, a siderophore used to sequester iron (“aerobactin”), community communication via quorum sensing (“quorum sensing”), hemolysin toxin production (“hemolysin”), and other toxin-producing genes (“other toxicity”). A complete list of gene symbols with detailed names and functions is provided in Table S12. + after MAG names indicates *Mycoplasma* MAGs previously associated with the body cavity of the cestode; * indicates Mycoplasmataceae and Vibrionaceae reference species known to be pathogenic in fish; ** indicates Vibrionaceae reference species known to be pathogenic in humans.

To understand how the identified associations have been shaped through interactions between gut microbes and the salmon host, we compared the genomes of the cestode-associated mycoplasma MAGs (MAG02–MAG05) with MAG01 *M. salmoninae* and three related *Mycoplasma* references using two *Ureaplasma* genomes as outgroups (Tables S10 and S11). Average nucleotide identity (ANI) was <85% between all pairs of genomes (Table S10), supporting the conclusion that each *Mycoplasma* MAG forms a separate species. The *Mycoplasma* MAGs clustered into three clades (Fig. 5A): MAG01 (*M. salmoninae*) was most similar to *Mycoplasma iowae* and *Mycoplasma penetrans*, as previously reported (19, 33, 34); MAG03 and MAG05 were more similar to the known fish pathogen *Mycoplasma mobile* (hereafter referred to as the “*M. mobile* clade”); and MAG02 and MAG04 formed a novel, more divergent clade (the “MAG02/MAG04 clade”).

A number of virulence factors have been characterized in the *Mycoplasma* genus, although much of the focus in the literature is on pathogens of terrestrial animals (41). We focused on those factors involved in adaptations for the utilization of specific nutrients, production of hydrogen peroxide, colonization and adherence to host cells, and toxin production (Fig. 5A). As previously reported (19), genomes in the *M. mobile* clade lacked the three genes required to generate ATP from the metabolism of arginine, in contrast to *M. salmoninae* and the MAG02/MAG04 clade. All three *M. mobile* clade genomes contained genes required for the uptake and metabolism of glycerol, a potential alternative energy source (42). However, only *M. mobile* had glycerol-3-phosphate oxidase, which generates cytotoxic hydrogen peroxide through glycerol metabolism. One putative adhesin (MHJ_0461) was conserved across *Mycoplasma* genomes. The *M. mobile* clade genomes also contained additional genes reported to be involved in adherence (41). Focusing on other virulence factors, the MAG02/MAG04 clade genomes included genes with sequence similarity to secreted exfoliative toxins, which are produced by *Staphylococcus aureus* to disrupt the epithelial cell layer during skin infections (43). In contrast, the *M. mobile* clade genomes included genes similar to the virulence factor *BrkB*, which is part of an autotransporter of *Bordetella pertussis* that confers resistance to killing by the classical complement pathway (44). Despite the relatedness of the *M. mobile* clade, only the reference *M. mobile* contained the gliding protein *Gli349*, which provides *M. mobile* with its unique mechanism for gliding motility and is suggested to be important for its pathogenicity (45). As previously reported, *M. salmoninae* did not have genes with any similarity to these known *Mycoplasma* virulence factors (33).

We performed a similar analysis with the genome of the size-associated MAGs (MAG06 *P. phosphoreum* and MAG08 *Aliivibrio*), compared to the other Vibrionaceae MAGs (MAG07 *Photobacterium iliopiscarium* and MAG09 *Aliivibrio*) and seven related Vibrionaceae references (*Photobacterium damselae*, *Photobacterium profundum*, *P. iliopiscarium*, *Aliivibrio fischeri*, *Aliivibrio wodanis*, and *Aliivibrio salmonicida*), using three additional *Vibrio* genomes as outgroups (*Vibrio cholerae*, *Vibrio splendidus*, and *Vibrio anguillarum*). For all three genera, we aimed to include a range of reference species that included known fish pathogens and commensal or environmental marine species (Fig. 5B; Table S11).

In the *Photobacterium* genus, MAG06 and MAG07 shared >98% ANI to the reference *P. phosphoreum* and *P. iliopiscarium* genomes, respectively, supporting their classification as these species (Table S10). *P. iliopiscarium* has been previously isolated from the gut microbiota of various Norwegian marine fish species, including Atlantic salmon, and has not been associated with disease (46). *P. phosphoreum* has been found in association with the skin and gut of healthy salmonids and other fish (47, 48). Both species were more distantly related to the free-living seawater species *P. profundum* and the fish skin pathogen *P. damselae* (Fig. 5B). As previously reported (49), the luminescence genes forming structural parts of the luciferase operon (*luxCE*) were present in the *P. phosphoreum* genomes, while they were absent from the other *Photobacterium* genomes (Fig. 5B). The *P. iliopiscarium* genomes contained more known hemolysins than *P. phosphoreum*, but such toxins were also present in the non-pathogenic *P. profundum*.

In the *Aliivibrio* genus, MAG10 shared 97.6% ANI with the reference genome of the salmonid pathogen *A. salmonicida* (Table S10). The *A. salmonicida* genomes grouped with the salmonid skin pathogen *A. wodanis*, rather than the commensal cephalopod symbiont *A. fischeri* (50) (Fig. 5B). MAG08 and MAG09 shared approximately 85% ANI with each other and with *A. wodanis*, supporting their classification as separate species (Table S10). Both MAGs grouped with the putative pathogenic *Aliivibrio* reference species rather than the commensal *A. fischeri* (Fig. 5B). Previously, chitin degradation has been associated with *Aliivibrio* commensalism or symbiosis with marine invertebrates, such as copepods or cephalopods (51). Consistent with previous reports (52), *A. salmonicida* did not have a complete chitin degradation pathway (Fig. 5B). MAG08 and MAG09 were also missing key components of this pathway, unlike the cephalopod-associated *A. fischeri*, suggesting that these *Aliivibrio* spp. are not adapted to colonization of such invertebrates. The *luxCE* genes required for the bioluminescence associated with *A. fischeri*–cephalopod symbiosis were not observed in MAG08 and MAG09. However, all *Aliivibrio* genomes contained other common quorum-sensing autoinducers and regulators. MAG08 had a complete D-glucuronate degradation pathway, while this pathway was incomplete in other *Aliivibrio*. Gut microbes can use this pathway to utilize host glucuronate as a carbon source (53), suggesting a possible adaptation of MAG08 to the salmon gut environment. Some toxins and toxin-related systems, including hemolysin III, tlyC, and type I secretion system, were conserved across *Aliivibrio* genomes. However, there were no clear patterns between the presence of toxicity genes and known pathogens.

## DISCUSSION

The gut microbiota of the piscivorous Atlantic salmon is characterized by low biomass and low diversity (34, 36, 38, 54), and thus is potentially easily disrupted by factors like immune dysregulation, infections, therapeutics, and dietary changes (2). In our large cohort of farmed salmon, we observed clear differences in the gut metagenomes of large, non-parasitized fish compared to small, parasitized individuals, which correlated with variation in the salmon genome, transcriptome, and metabolome. A single *Mycoplasma*, MAG01, dominated large, non-parasitized fish, consistent with previous studies showing high levels of *Mycoplasma* in the gut microbiota of both farmed and wild Atlantic salmon (19, 33–39, 54). While small and/or parasitized salmon also had high abundance of MAG01, we observed increased alpha diversity in these individuals, driven by increased frequency of low-abundance Vibrionaceae and other *Mycoplasma* MAGs. Our multi-omic approach identified an increased abundance of genes and metabolites associated with the emerging MAGs. While increased microbial diversity is often assumed to indicate a “healthier” gut microbiome state based on studies in mammals, such microbial processes are not always applicable to other host taxa (55, 56). Thus, changes in alpha diversity should always be evaluated within the context of each study system (57, 58). Studies in salmonids suggest that gut microbial diversity decreases after the transition to the marine life stage and remains low in adults, with generally less than a dozen bacterial taxa dominating the microbiome composition (38, 54). A few studies in salmonids have also shown low or decreased gut microbial diversity in healthy fish compared to those with bacterial infections (35, 59). Together with this evidence from previous studies, our results suggest that the gut microbiota of the small and/or parasitized fish in our study is in a state of dysbiosis, which we discuss in more detail below.

### Cestode-associated *Mycoplasma* in parasitized fish

Parasitic helminth infections are frequently associated with gut dysbiosis in animals (13, 14, 60–62). However, it is often not clear whether prior dysbiosis promotes subsequent parasite colonization or whether the parasite triggers dysbiosis, e.g., by direct interaction with the host microbiota, introduction of new microbes to the host–gut or indirect dysregulation of the immune response (11). In our study, we identified four *Mycoplasma* MAGs that were strongly associated with cestode detection. Low-abundance *Mycoplasma* MAGs have been identified in the gut metagenomes of wild Norwegian Atlantic salmon colonized by cestodes (34). We have also previously identified two of these *Mycoplasma* MAGs closely associated with the body of cestodes from our farmed salmon cohort, specifically MAG02 and MAG03 (19) (Table S2). Comparing the genomes of the four cestode-associated *Mycoplasma* MAGs with the dominant MAG01, we found that the former contained more genes typically associated with virulence in *Mycoplasma*, particularly the *M. mobile* clade (MAG03 and MAG05). In contrast, MAG01 generally had a reduced genome with fewer genes associated with *Mycoplasma* virulence. Our results suggest that MAG01 is well adapted to non-pathogenic colonization of the salmon gut, as previously reported (33, 54), whereas we hypothesize that the cestode may introduce novel, potentially more virulent, *Mycoplasma* MAGs into the salmon gut.

In the GWAS, we identified one strong association between the detection of cestode-associated *Mycoplasma* MAG05 and a 1.8-Mbp region of the salmon genome that encoded several long non-coding RNAs (lncRNAs) and three annotated genes. While the direct effects of these variants are currently unclear, lncRNA expression profiles have been previously associated with specific gut microbiota compositions in mice (63), possibly through their involvement in inflammation and other immune responses (64–66). Thus, different lncRNA genetic variants may have altered expression profiles, suggesting a possible mechanism for host control of the microbiota. Given that MAG05 was strongly associated with cestode detection, we hypothesize that during alteration of the gut microbiota by cestode infection, the ability of MAG05 to establish in the salmon gut may be at least partially determined by the genetic background of the host. Our transcriptome data set only included mRNA transcripts; thus, we were unable to investigate lncRNA expression patterns. However, we did observe one gene in the region of interest on chromosome 5, ANKHD1-like, with weakly altered expression when MAG05 was present, depending on host genotype. A high level of RNA degradation in the transcriptome samples likely limited our ability to detect other changes in gene expression. Future studies using, e.g., whole transcriptome long-read RNA sequencing, would do well to further investigate both mRNA and lncRNA loci in order to shed more light on links between RNA expression patterns and microbiota composition. While the mechanisms may be unclear, our results suggest biological control of the microbiota by the host, explained here by host genotype. Similar associations between SNPs in specific host genes and abundance of specific gut microbes have been observed in recent, large-scale human studies (5, 6). Cestode alternation of the gut microbiota has also been shown to depend on host genotype in sticklebacks (67), further supporting our results.

The MOFA revealed a clear response in the gut environment during cestode infection, with increased frequency of cestode-associated *Mycoplasma* MAGs, increased abundance of bile acid metabolites and altered expression of two genes in taurine metabolism pathways. Bile acids are involved in a range of important functions and processes in fish, including the digestion of lipids, vitamins and carotenoids, cholesterol regulation, inflammation regulation, intestinal barrier function, and antimicrobial activities (68). Primary bile acids are produced from cholesterol in the liver, where they are conjugated with predominantly taurine to form bile salts, before transport and excretion into the intestine (68). Some members of the gut microbiota can deconjugate and further modify these bile salts into secondary bile acids, which can have additional effects on both the host and specific gut microbes (69, 70). Cestodes may use host-derived bile acids or salts as a nutrient source (71); however, bile acids may also limit core cestode metabolic pathways (72). In Atlantic salmon, lower levels of body cholesterol and bile acids have been associated with increased intestinal inflammation, suggested to be linked to changes in dietary taurine and cholesterol (73). As our results were consistent between feed types, we suggest that bile acids play an important role mediating host–microbiota–parasite interactions in our system regardless of diet.

### Size-associated Vibrionaceae in small fish

Variation in growth rates in salmon raised under identical conditions can be due to multiple biotic factors, including differences in growth hormone production, appetite, feed conversion efficiency, response to stress, behavior, and infectious disease burden (31, 74–81). Selective breeding programs have increased domesticated salmon growth rates (82); however, since growth traits are polygenic, understanding the genetic basis of growth in salmon remains a complicated challenge (29, 30). Links between the gut microbiota and salmon growth have mostly been investigated in relation to the effect of different diet compositions (54, 83–86). In contrast to these studies, we investigated how salmon genetic variation and gut microbiota composition may affect growth in an integrated manner, under standard farming conditions.

While we found no strong associations between size-associated MAGs and host genetic variability in the GWAS, we observed a correlated set of size-associated changes in the salmon metagenome, transcriptome, and metabolome after controlling for parasite presence. Some of the most important size-associated microbes were Vibrionaceae MAGs, including species of *Photobacterium* and *Aliivibrio*, which were more often detected in small fish. Both *P. phosphoreum* (MAG06) and *P. iliopiscarium* (MAG07) are known to colonize salmonid guts in the absence of disease (46–48), and the genomes of these MAGs were not enriched for known virulence factors. In contrast, an increased abundance of *Aliivibrio* species in the gut microbiota has been previously found during bacterial infections in farmed Atlantic salmon (35, 87), and *A. salmonicida* (MAG10) is a known salmonid pathogen (88). However, we observed that the most abundant *Aliivibrio* (MAG08) carried no additional virulence factors, although its glucuronate degradation pathway suggests adaptation to survive in the salmon gut. Thus, from our results, it is unclear whether these Vibrionaceae species are directly pathogenic or simply opportunistic colonizers during dysbiosis.

Our multi-omic data set enabled us to generate new connections between host size and immune response to specific microbes. In the MOFA, we identified a set of transcripts that were associated with size class and the detection of Vibrionaceae MAGs, although the genus/species of MAG varied with feed type. Many of the genes with increased expression in small fish colonized by Vibrionaceae encode proteins in immune pathways, including ladderlectin, cathelicidin, and apolipoproteins. Ladderlectin is an antimicrobial lectin in teleost fish that has been shown to bind lipopolysaccharides (LPS from Gram-negative bacteria, like Vibrionaceae species, in rainbow trout (89, 90). The Atlantic salmon ladderlectin genes share significant homology with rainbow trout, suggesting a similar function (90). Cathelicidins are antimicrobial peptides that have been shown to have an important role in the response to the Gram-negative salmonid pathogen *Yersinia ruckeri* (91). Apolipoproteins bind lipids and are therefore involved in lipid transportation (92); however, they are also involved in innate immunity in teleosts against both Gram-negative and Gram-positive bacteria, as well as parasites (93–96). Furthermore, an increased abundance of *Photobacterium* has been associated with decreased expression of gut barrier function genes in farmed Atlantic salmon (97). Our results are therefore consistent with an innate immune response in small fish, triggered by Gram-negative bacteria.

MOFA analysis also revealed that the majority of variation in the salmon gut transcriptome and metabolome (>20% in each) was explained by factors strongly correlated with size class, independent of microbiota composition. Metabolites consistently associated with large fish included acyl carnitines and fatty acids, which have important roles in energy production and storage, among other functions (98, 99). We identified a set of genes whose expression was associated only with small fish, with roles in cell division and cytoskeleton and extracellular matrix signaling, such as actin, tensin, and myosin. Changes in myosin gene expression have particularly been linked to changes in growth rate in farmed Atlantic salmon (100). The gene set also included thrombospondins, immune genes that interact with the extracellular matrix and inhibit angiogenesis, an important process in growth and development (101). Our results demonstrate that different growth-related metabolic and development processes occur in fish with different lifetime growth phenotypes.

### Future perspectives

Understanding the mechanisms driving host–microbe interactions and their impact on host phenotypes is an ongoing challenge. In this study, we use correlative methods to identify hitherto hidden associations among host and microbial factors in an applied, real-world setting. However, this study design does not allow the complete control of environmental variables or host genetics. Thus, we were unable to untangle the relative contribution of diet and pen environment (represented by the feed type variable) and host genotype to our observed host–microbe interactions, nor determine a causative relationship between host–microbe associations and phenotype. Future experimental studies where both environment (including diet) and host genetic background are controlled could further elucidate the mechanisms behind the associations identified here. For example, genetic crossing experiments coupled with common garden setups could be used to study the effect of broad genetic variation in a population on host–microbe interactions (e.g., see reference 67), while gene knockouts could be used to determine the effect of changing specific genes or alleles on host–microbe interactions (e.g., see reference 102). Such mechanistic investigations could complement explorative, large -omics studies like ours to provide a more detailed understanding of host–microbe interactions from the holobiont perspective.

### Conclusions

Our hologenomic approach to study gut health in farmed Atlantic salmon revealed a clear association between cestode infection, lifetime growth, and gut dysbiosis. This dysbiosis was associated with altered expression patterns in the salmon transcriptome and metabolome, including changes in immune, taurine synthesis and bile acid pathways. We suggest two non-exclusive hypotheses to explain these findings. First, the cestode may colonize the salmon gut, altering the gut microbiota and resulting in an altered host response, possibly with changes to host growth phenotypes. Second, small fish may be already stressed with a dysregulated gut environment, resulting in lowered host control of the intestinal microbiota, allowing parasites and/or putative opportunistic pathogens to establish or even outgrow commensals in the salmon gut. Furthermore, our results suggest that the genetic background of the salmon may in part determine how the gut microbiota changes during dysbiosis. These results highlight the value of the hologenomic approach for understanding how the response of an intestinal microbiota community to parasite infection may depend on the genetic background of the host organism.

## MATERIALS AND METHODS

### Experimental design and sampling

Samples used for this study were obtained as part of the HoloFish project (Norwegian Seafood Research Fund, project no. 901436). This cohort has been described previously (19). Briefly, we sampled 460 ready-to-harvest Atlantic salmon from a commercial production site close to Bergen, Norway, owned by Lerøy Seafood Group during April 2018. Samples were obtained from two groups reared in separate sea pens and fed two different standard commercial diets (“feed1” and “feed2”). The exact composition of these diets is proprietary and unavailable, but they were manufactured by BioMar and EWOS, respectively, in 2018. For feed1, salmon were fed BioMar’s “Energy X” from sea transfer until they were 1 kg in weight, “Power 1000” from 1.0 to 2.5 kg and “Power 2500” from 2.5 kg to slaughter. For feed2, salmon were fed “EWOS Rapid” from sea transfer until they were 1 kg in weight and “EWOS Extra” from 1 kg to slaughter. A number of relevant metadata variables describing various phenotypes of each fish were recorded (Data S5), including gutted weight (kg) and intestinal cestode infection status using a cestode index from 0 to 3, where 0 = no observed cestode; 1 = one to three visible cestodes; 2 = more than three cestodes, but digesta volume was higher than cestode volume in the gastrointestinal tract; and 3 = excessive numbers of cestodes dominating the gastrointestinal volume that were likely impeding the passage of feed along the gastrointestinal tract. Fish were further binned into size classes (small, medium, and large) based on gutted weight and cestode detection classes (present or absent) using the above-mentioned cestode index.

A record of all statistical analyses is included in (File S1) , created using the knitr package in R. Each statistical analysis is also described in the relevant sections below. We used a linear model to test for significant differences (*P* < 0.05) between gutted weight and cestode index, including feed type as a covariate. We used Fisher’s exact test to compare frequency of cestode detection between the two feed types.

Six biological samples were taken from each fish, including muscle tissue for fatty acid profiling, gill tissue for host genomics, gut epithelia for host transcriptomics, gut epithelial cell scrapes for 16S metabarcoding, and two gut content samples for metagenomics and metabolomics. We also sampled the current feed pellets for each feed type (feed1: 10 pellets of BioMar’s Power 2500 and feed2: 10 pellets of EWOS Extra) for profiling the feed microbiota using 16S metabarcoding to identify potential contaminant microbes sourced from the feed.

### Host genomics

Between 10 and 20 mg of salmon gill tissue was used for DNA extraction, using Qiagen DNeasy blood & Tissue Kit 96, following the manufacturer’s recommendations. Genomic DNA was fragmented using a Covaris LE220+, aiming for 350 bp. For library preparation, 100 ng of DNA per sample was used in a single-tube library preparation method (103). Libraries were quantified with quantitative PCR (qPCR) to determine the required number of cycles needed for indexing PCR. Purified libraries were indexed and amplified using customized index primers for MGISeq-2000 (104). Indexed libraries were purified using magnetic SPRI beads (105). Prepared libraries were shipped on dry ice to BGI-Shenzhen for sequencing on MGIseq-2000 using paired-end (PE) 150-bp chemistry.

Adapter and quality trimming was performed using AdapterRemoval v.2.2.4 (106), trimming consecutive bases with quality scores (QS) of <30 and removing reads with >5 ambiguous bases (Ns) or <100 bp after trimming. Duplicate reads were removed with SeqKit rmdup (107). Reads were aligned to the Atlantic salmon reference genome (GCA_905237065.2) with bwa mem v.0.7.16 (108). Paired reads mapping to the reference were retained with samtools v.1.9 (109). Multiple BAM files from the same individual (including host-mapping reads from the metagenomic sequencing) were merged with samtools merge, and mapping duplicates were removed with Picard MarkDuplicates v.2.25.0 (https://broadinstitute.github.io/picard/). To identify callable regions of the genome, 10 samples were randomly selected and combined into a single BAM file with samtools merge. Read groups were reformatted with Picard v.2.20.2. Coverage statistics were evaluated with samtools depth. Based on these values, callable loci were identified using GATK CallableLoci v.3.7 (110) using a minimum depth of 26 and a maximum depth of 158. Genotype likelihoods were then estimated for all samples with the SAMtools model in ANGSD v.0.933 (111) using the identified callable loci regions. From these, genotype probabilities were imputed with Beagle v.3.3.2 (112) and converted to genotype dosages. Linkage disequilibrium pruning was performed with PLINK v.2 (https://www.cog-genomics.org/plink/2.0/), removing all correlated SNPs with *R*^2^ of >0.5.

### Host–gut transcriptomics

Host transcriptomes were extracted using 20 mg of gut epithelia with Quick-RNA Miniprep Kit (Zymo Research), following the manufacturer’s recommendations. Prior to extraction, samples were washed with phosphate-buffered saline buffer to remove residual RNAlater buffer. Subsequently, samples were shipped on dry ice to Novogene for polyA enrichment and mRNA library preparation and sequencing. Briefly, messenger RNA was purified from total RNA using poly-T oligo-attached magnetic beads. Purified RNA was fragmented via sonication. The first strand of cDNA was synthesized using random hexamer primers, followed by a second cDNA synthesis. On this cDNA, a library was then prepared via A-tailing, adapter ligation, PCR amplification, size selection, and purification. The resulting purified library was quantified with a Qubit Fluorometer and real-time PCR, and an Agilent BioAnalyzer 2100 was used to determine the fragment size distribution and RNA integrity number (RIN). The overall quality of the libraries was low (RIN median: 2.5, range: 0–10; Data S1); thus, we decided to proceed with the sequencing of all libraries and rely on post-sequencing measures to remove low-quality sample (see below). All libraries were therefore pooled, and 150-bp paired-end reads were sequenced on an Illumina NovaSeq 6000 instrument.

Sequence quality of raw RNA-Seq data were assessed using FastQC v.0.11.3 (http://www.bioinformatics.babraham.ac.uk/projects/fastqc/). Quality trimming was performed using AdapterRemoval v.0.20.4 (106) to remove base pairs with a Phred score of <20 and trimming of poly-A tails of >8 bp. Sequences shorter than 55 bp and all unpaired reads were excluded from subsequent analyses. The quality of trimmed sequences was checked again using FastQC v.0.11.3. Reads were then aligned to the Atlantic salmon reference genome (GCA_905237065.2) using STAR aligner (113) and default parameters. As RNA degradation was present in most samples, aligned reads were used to generate a gene-specific count matrix across samples using DegNorm (114). DegNorm adjusts the read counts for transcript degradation heterogeneity while controlling for the sequencing depth. We used a post hoc analysis to generate coverage curves before and after DegNorm normalization for a subset of genes of interest, as identified in the analyses outlined below (Fig. S11). Likely due to the RNA degradation, many samples had low mapping statistics to the reference genome; thus, we excluded all samples with <50% of reads uniquely aligned to the reference. To reduce noise in the data set, we only included transcripts with at least 10 counts in at least 50% of the samples included in downstream analyses.

We used the R package DESeq2 (115) to estimate library size factors, using “shorth” as the locator function from the genefilter R package, which is the shortest interval that covers half of the adjusted count values in each sample. For unsupervised analyses (ordination and MOFA), we used the DESeq2 function “vst” to normalize adjusted counts by the above size factors, estimate gene dispersions and apply the variance-stabilizing transformation (VST) to make the data set approximately homoskedastic and bring the values into log2 space. This transformed data set was then used as input for the R function prcomp to generate a PCA. Permutational multivariate analysis of variance (PERMANOVA) was performed on Euclidean distances of the transformed data set using the adonis2 function in the R package vegan v.2.6–2 (https://github.com/vegandevs/vegan), testing for associations with size class, cestode infection index, and feed type (Table S1). The transformed data set was also used for feature selection in MOFA (see below).

For differential expression analysis, the DESeq function was applied to the adjusted count data, with negative binomial generalized linear models and Wald significance tests. DESeq automatically incorporates size factors and gene dispersions into the models. Significantly differentially expressed genes (adjusted *P* < 0.05) between various fish phenotype variables (e.g., size class and host genotype, detailed below) were extracted from the results.

### Gut metagenomics

DNA was extracted from approximately 100 mg of each gut content sample using the ZymoBIOMICS 96 MagBead DNA Kit (D4308) (Zymo Research), following the manufacturer’s recommendations. Before extraction, samples were resuspended in 1× Shield (Zymo Research). To minimize batch effects, all samples were randomized before any laboratory processing. We also included seven negative controls containing only Shield (Zymo Research) in the extractions to identify putative laboratory reagent contaminants in downstream microbial community analyses. Libraries were initially prepared for BGI sequencing, following the same single-tube library preparation method (103) as for the host genome. Prepared libraries were shipped on dry-ice to BGI-Shenzhen for sequencing on MGIseq-2000 using PE 150-bp chemistry. To expand the sample number and sequencing depth for the metagenome, aliquots of the DNA extracts described above were also shipped on dry ice to Novogene for Illumina library preparation and PE 150-bp sequencing.

Adapter and quality trimming was performed using AdapterRemoval v.2.2.4 (106), trimming consecutive bases with quality scores of <30 and removing reads with >5 Ns or <100 bp after trimming. Duplicate reads were removed with SeqKit rmdup (107). Reads were aligned to the Atlantic salmon reference genome (GCA_905237065.2) with bwa mem v.0.7.16 (108). Unmapped paired-end reads were extracted from the BAM files with samtools v.1.9 (109). Data from both BGI and Illumina were combined to generate the MAG catalog. Both co-assembly and single-assembly approaches were used. For co-assembly, samples were divided by feed type. Samples within each group were co-assembled with Megahit v.1.2.9 (116), using a minimum contig length of 1,000 bp in the metasensitive mode. Reads from all samples and feed types were aligned back to each co-assembly with bwa v.0.7.17. Binning was carried out with MetaBAT2 (117), MaxBin v.2.0 (118), and CONCOCT (119) within the wrapper metaWRAP v.20200226 (120). Bins were evaluated using single-copy marker genes as part of the classify workflow in checkM v.1.1.3 (121), which provided completion and redundancy percentages for each bin. The best bins for each assembly were identified with metawrap’s refinement tool, including only bins that were estimated to be >50% complete and <10% redundant. As a measure of overall bin quality, the QS was calculated using checkM statistics: bin QS = bin completeness – 5 × bin redundancy (122). Six bins with QS of <50% were targeted for further refinement via single assembly. One sample with high coverage for each bin and low coverage of all other bins was selected for single assembly with metaSPADES v.3.14.0 (123). Reads from 92 randomly selected samples (and the 6 used for single assembly) were aligned back to each single assembly with bwa v.0.7.17. Binning and refinement were carried out with metaWRAP as described above. We also inspected and manually refined bins using the Anvi’o platform (124). The co-assembled and single-assembled bins were then combined with bins generated in previous salmonid studies (19, 33). This list of 33 bins was then dereplicated at the approximate species level (ANI >95%), and a representative bin per “species” was identified with dRep v.3.2.0 (125). These 16 representative bins form the final MAG catalog used in all subsequent microbiota analyses. The MAG catalog was taxonomically annotated with GTDB Tk v.2.1.0 (126) using the GTDB r207 reference database (127). For each MAG, open reading frames were identified with Prodigal (128); single-copy core genes were identified with HMMER (129); and genes were annotated using the Pfam (130) and Kyoto Encyclopaedia of Genes and Genomes (KEGG) Orthologs databases (131), all within Anvi’o. The Illumina reads from each sample were then aligned back to the final MAG catalog with bwa mem v.0.7.17. Anvi’o v.7.0 was then used to extract coverage and detection statistics for each sample’s alignment to each MAG.

Negative controls had generally low numbers of reads aligned to the MAG catalog (median: 27,720; range: 3,818–106,380). Based on this comparison, we excluded gut samples with <100,000 reads aligned to the MAG catalog from further analysis to avoid samples potentially biased by too high a fraction of contaminant reads. For all downstream analyses (microbial community analyses, GWAS, and MOFA), we defined a MAG as detected in a sample if ≥30% of the nucleotides in a MAG were covered by at least one read in that sample (132). MAGs with lower detection values were defined as not detected in that sample, and their abundance was set to 0. One MAG (*Prevotella*) was assembled but was not detected in any sample after this filtering and is therefore not included in any analysis. We used Anvi’o “abundance” values to represent normalized MAG abundance, defined as the mean coverage of a MAG in a sample divided by the overall mean coverage of all MAGs in a sample. For scaling in some downstream analyses, we then applied a log10 transformation to the abundance values (with a pseudo-count of 0.001 for zero values).

We compared MAG abundances between samples and the negative controls. After filtering, no MAGs were detected in five of the seven negative controls. The remaining two negative controls had MAG01 present at detectable abundances after filtering (47–54 normalized mean coverage). No other MAGs were detected in these two controls. Since MAG01 was the most abundant MAG in our samples, this infrequent detection is likely due to cross-contamination during laboratory processing (133). Furthermore, MAG01 was not detected at all in the other five negative controls. Thus, we did not exclude MAG01 as a contaminant.

### 16S metabarcoding

Metabarcoding data of the 16S ribosomal RNA (rRNA gene) was generated for 140 gut epithelial scrapes. The sample collection was composed of gut content (60), gut tissue (60), and pellets of each feed type (10 extraction replicates for each feed type). First, DNA was extracted using the ZymoBIOMICS DNA/RNA MiniPrep kit (ZRC201962). For the gut content, 750-µL Shield (Zymo Research) was used as input for the extraction. For the gut tissue and feed types, approximately 150 mg was used as input for the extraction. Subsequently, the DNA sample extracts were screened for inhibition using qPCR (134) with bacterial 16S primers V3–V4 (135). Moreover, the Ct values, as well as amplification plots, generated from the qPCR were used to estimate the number of tagged PCR cycles. Next, the samples were amplified using tagged PCR (136) with the bacterial 16S primers V3–V4 designed with unique oligonucleotide tags. Thus, each amplified sample was tagged with a unique tag combination to identify potential tag-jumping errors (137). The gut content and feed types were given 25 cycles of tagged PCR, whereas the gut tissue were given 30 cycles of tagged PCR. The tagged PCR amplicons were then pooled according to gel band visibility and purified using SPRI beads (138). The purified amplicon pools were built into libraries using the PCR-free protocol TagSteady (139). Subsequently, the libraries were quantified using a 2100 BioAnalyzer Instrument (Agilent) and pooled equimolar. Ultimately, the library pool was sequenced on a Illumina MiSeq lane aiming for 107 Mb per sample of PE 350-bp data. Negative controls were included in all steps, from DNA extraction to library build, to detect potential microbial contaminants.

Sample demultiplexing and adapter removal were performed using AdapterRemoval v.2.2.4 (106). Using the pipeline BAMSE v.1.0 (https://github.com/anttonalberdi/bamse), primer clipping was performed by Cutadapt v.2.10 (140), followed by read orientation, read quality trimming and read filtering. DADA2 v1.17.3 (141) was used for error learning and correction, dereplication, paired-end read merging, and generation of amplicon sequence variants (ASVs). Chimeric ASVs were filtered by DADA2 and the remaining ASVs were assigned taxonomy at the genus level using the SILVA non-redundant SSU database v.138. ASVs assigned to eukaryotes (including mitochondria or chloroplast sequences) were removed, as were ASVs with a relative number of copies of <0.01% in each sample. Putative contaminant sequences from laboratory processing were identified and removed with decontam v.1.8.0 (142).

To compare the 16S community composition between gut content, gut mucosa, and feed pellet samples, we focused on ASVs present in at least five samples after quality control (Fig. S7). We generated a heatmap of ASV abundance using the R package pheatmap. ASV read counts per sample were normalized to abundance values using the centered log-ratio transformation. We also generated a Venn diagram using the R package ggVennDiagram, displaying the number of ASVs detected in and among each sample type.

### Gut metabolomics

Gut content samples were cryo-homogenized in 25% water, 25% methanol, and 50% dichloromethane in a 1:15 sample:solvent ratio (wt/vol). Homogenization was carried out using an OMNI Bead Ruptor 24, using liquid nitrogen to keep homogenized samples below 0°C to minimize degradation of metabolites during extraction. Homogenates were centrifuged at 20,000 × g (0°C), and the polar phase from all samples was concentrated using SpeedVac (ThermoFisher Scientific) and resuspended in 200-µL 5% methanol. Four procedural blanks were included in homogenization. A volume of 100 µL of all samples was collected into a quality control sample used for normalization to enhance the detection of metabolites. Samples were measured on a nano-flow ultra-high-pressure liquid chromatography–tandem high-resolution mass spectrometry analysis. Metabolites were detected using a Q Exactive HF Hybrid Quadrupole-Orbitrap mass spectrometer (ThermoFisher Scientific) operated in positive ion data-dependent acquisition mode (143).

ThermoFisher Scientific UHPLC-Orbitrap-MS/MS RAW files were converted into mzML files using Proteo Wizard (144). For an increased deciphering of molecular spectra, MZmine2 (145, 146) was applied for mass detection of MS1 and MS2 spectra, followed by chromatogram detection and deconvolution. Subsequently, detected isotopes and features were grouped according to a tolerance of mass charges (5 ppm for *m*/*z*) and retention time (6 s) and the features were further aligned according to retention time and *m*/*z*. Lastly, only features with a MS2 spectrum were kept for further substructural analysis and *in silico* analysis. A molecular network was created using the feature-based molecular networking workflow on the Global Natural Product Social Molecular Networking (GNPS) platform (147, 148) combined with SIRIUS4 and CSI:FingerID (149, 150), as previously described for fish intestinal metabolomics (59). In brief, to enhance identification of unknown metabolites, unsupervised substructures were discovered using MS2LDA (151, 152), and MS2 spectra were annotated *in silico* using Network Annotation Propagation (153). Furthermore, peptidic natural products were annotated *in silico* using DEREPLICATOR (154). Chemical classes were retrieved for all GNPS library hits and *in silico* structures using ClassyFire (155). Finally, all structural annotations were combined within one network using MolNetEnhancer (156).

Metabolites detected in <50% of all fish samples were filtered out to reduce zero inflation issues in the data set, and samples with >80% missing data were also excluded. Abundance data were then further processed in the R package MetaboDiff (157), where missing data were imputed for all metabolites detected in at least 60% of samples. Abundance data were normalized across samples to generate relative abundances. The PCA generated from these normalized abundances revealed strong batch effects, a common problem in metabolomic data (158). For the PCA and inclusion of metabolomics in the unsupervised MOFA analysis, we therefore used limma’s function removeBatchEffect (159) to regress out the metabolomic batch effect from the normalized data. PERMANOVA was performed on Euclidean distances of the normalized abundances, both before and after the removal of batch effects, using the adonis2 function and testing for associations with size class, cestode infection index, feed type, and batch (Table S1).

### Fatty acid profiling

The fatty acid composition was analyzed in both the feed pellets and fish muscle samples. Lipids from the samples were extracted by adding chloroform-methanol (2:1, vol/vol), and 19:0 methyl ester was added as an internal standard. After extraction of lipids, the samples were filtered, saponified, and esterified using 12% BF_3_ in methanol. Fatty acid methyl esters were separated on a gas chromatograph and detected using a flame ionization detector, as described earlier (160, 161). The fatty acids were identified by retention time using standard mixtures of methyl esters (Nu-Chek, Elyian, USA), and quantified by the internal standard method.

For downstream analyses, the relative abundance (%) of each fatty acid was log10 transformed. The transformed data set of fish samples was then used as input for the R function prcomp to generate a PCA. PERMANOVA was performed on Euclidean distances of the transformed data set using the adonis2 function, testing for associations with size class, cestode infection index, feed type, and fatty acid batch (Table S1).

### Microbial community analysis

Alpha diversity from MAG abundance values was calculated using the Hill numbers framework with the R package hillR, using *q* = 1 to estimate the Shannon index (162). We used a linear model to test for statistically significant differences in the Shannon index by cestode detection and size class while controlling for feed type and sequencing depth.

Non-metric multi-dimensional scaling (NMDS) was performed using log10-transformed abundance values and Euclidean distance with the ordinate function in the R package phyloseq v.1.40.0 (163). The NMDS stress value, which is a measure of the degree to which the distance between samples in the reduced dimensional space corresponds with the actual distance between samples (similar to a goodness-of-fit value), has been included in the figure legend of each NMDS plot. The *k* value (number of dimensions) is also given in each NMDS plot. PERMANOVAs were performed on the Euclidean distances using adonis2. NMDS using MAG presence/absence data and Jaccard distance was performed with the metaMDS function in vegan, and PERMANOVAs were performed on the Jaccard distances using adonis2. PERMANOVA models are provided in Table S1.

We used MaAsLin2 (164) to identify differences in MAG abundance and MAG detection between cestode detection and size class while controlling for feed type and sequencing depth as fixed effects and sampling date as a random effect. For MAG abundance, MaAsLin2 was run with “normalization = none” and “transformation = log” parameters, whereas for MAG detection, “transformation” was also set to “none.” We classed associations as statistically significant with a false discovery rate (also called a *q* value) of <0.05; however, we report all associations with FDR of <0.2 in Table S4.

For subsequent analyses, we used MAG abundance data (excluding MAGs detected in <50 samples) and MAG detection data (i.e., presence/absence, excluding MAGs detected in <10 samples). We also defined three additional “MAG detection” variables: detection of any low-abundant mycoplasma MAG (i.e., MAG02–MAG05), detection of any *Photobacterium* MAG (MAG06–MAG07), and detection of any *Aliivibrio* MAG (MAG08–MAG10). Since MAG01 (*M. salmoninae*) was detected in all but 3 of the 392 metagenomes, we also defined a fourth MAG detection binary variable, representing samples with high vs low abundance of MAG01 (> or ≤1.7 normalized abundance, respectively, based on the third quartile of the MAG01 abundance distribution).

### 16S profiles vs metagenomes

To confirm that our assembled MAGs captured most of the bacterial diversity in the gut content community, we compared our 16S ASV relative abundances to our MAG relative abundances. Since MAG and ASV taxonomy were assigned using different databases, we summed abundance across all gut content samples at the genus level (*Aliivibrio*, *Brevinema*, *Carnobacterium*, *Clostridium*, *Lactobacillus*, *Neilla*, *Photobacterium*, and *Psychromonas*), family level (Mycoplasmataceae and Vibrionaceae), and order level (Alteromonadales). Total sum scaling was used to bring all abundance values within each data set to a percentage (0%–100%). Relative abundances were then compared visually against 1:1 hypothetical perfect concordance (Fig. S6). All taxa from the MAG catalog are represented by at least one level in the plot, whereas all ASVs not in the above taxa lists were grouped as “other.”

### Genome-wide association study

To identify genetic variation underlying phenotype differences, a GWAS was performed with GEMMA (165), which uses linear mixed models to test for genome-wide associations. A centered genotype relatedness matrix was included in the model to account for the high relatedness among individuals in the population. We used a “leave-one-chromosome-out” strategy to prevent overcorrection of the model, where GEMMA was run on each chromosome separately and the current chromosome was not included in the relatedness matrix calculation. To account for wider population structure, principal components (PCs) were estimated with PCAngsd v20190125 (166), and the first five PCs were included as covariates in each GWAS run. Results were collated across chromosomes for the generation of QQ plots, inflation factor (λ), and Manhattan plots in R. SNPs with *P* values of <5 × 10^−8^ were classed as strongly associated with the phenotype of interest, while SNPs with *P* values of <1×1010^−5^ were classified as moderately associated.

For size investigations, gutted weight was transformed with the inverse normal transformation (INT) to approximate a normal distribution for linear regression in the model. Feed type (converted to a binary variable) was included as a covariate. GEMMA was also run with feed type as a phenotype, with gutted weight (untransformed) as a covariate. Detection of cestode (binary presence/absence) was also tested, including gutted weight (untransformed) and feed type as covariates. For microbiome (m)GWAS, microbiome phenotypes were included as binary variables (MAG detection) and INT-transformed (MAG abundance, alpha diversity) or untransformed (ordination dimensions) continuous variables. Feed type, gutted weight (untransformed), and cestode presence/absence were included as covariates along with PCs 1–5 for each mGWAS to account for wider population structure.

Only one microbiome phenotype was associated with host genetic variation in the mGWAS, specifically MAG05 presence or absence and 14 SNPs in a 1.8-Mbp region of chr5. The locations and annotations of candidate associated SNPs in the genome annotation file were identified with BEDtools v.2.30.0 (167), extracting gene and mRNA annotations overlapping (“bedtools intersect”) with the coordinates of each SNP. For SNPs located within a gene or mRNA transcript, further annotation information, e.g., Gene Ontology (GO) terms, was accessed using the R package AnnotationDBI v.1.58 to query the Atlantic salmon annotation stored in the R package AnnotationHub v.3.4 (organism AH100820, last updated on 21 April 2022). GO term descriptions were accessed using the R package GO.db v.3.15. The coordinate of each candidate SNP was also entered into the Ensembl Variant Effect Predictor tool (https://www.ensembl.org/Tools/VEP) to determine whether the SNP (relative to the reference genome) resulted in a synonymous or non-synonymous mutation in a coding region or if it fell in an intron or non-coding region.

To investigate the effect of these SNPs on gene expression, we used the genotype probabilities to define genotypes for each individual at each of the 14 significant positions, using a genotype probability of >0.8 for categorizing an individual as major/major, major/minor, or minor/minor. We excluded individuals with genotype probabilities of ≤0.8 for all three genotypes. For SNPs located in genes that were sequenced in our transcriptomic data set, we compared expression of those genes (post-VST normalization) between individuals with the major/major, major/minor and minor/minor genotypes, and between fish with MAG05 present or absent. Statistically significant differences in gene expression (*P* < 0.05) were identified using Wilcoxon rank-sum tests implemented in the R package ggsignif (168). To identify more widespread effects of these SNPs on gene expression, we performed differential expression analysis using DESeq, as described in Host–Gut Transcriptomics, using the subset of 36 genes that were located within 1 Mbp in either direction of the chr5 SNP peak. Two of the 14 SNPs had the same genotype in all individuals included in the transcriptomic analysis and were therefore excluded from this analysis. We ran three different models in DESeq1: (i) the 12 SNP genotypes, (ii) MAG05 presence/absence, and (iii) the 12 SNP genotypes and MAG05 presence/absence. In all three models, we also included feed type, size class, and cestode presence/absence as covariates.

### Multi-omics factor analysis

MOFA was used to integrate the metagenome, metabolome, and transcriptome data sets, using the R package MOFA2 (40). For the metagenome data set, we included presence/absence data for all MAGs detected in >10 and <388 samples (11 MAGs) as well as the four additional MAG detection variables (any low-abundant *Mycoplasma* MAG, any *Photobacterium* MAG, any *Aliivibrio* MAG, and high vs low detection of MAG01). For the metabolome data set, we included the 500 most variable metabolite abundances (normalized as described in Gut Metabolomics). For the host transcriptome data set, we included the 500 most variable gene expression abundances (following DegNorm, DESeq2, and VST processing steps, as described in Host–Gut Transcriptomics). We trained the MOFA model with 15 factors, using Gaussian (metabolome and transcriptome) and Bernoulli (MAG detection) distributions, scaling each data set to have similar variances and otherwise using default values. To identify associations that were independent of feed type, we separated samples into two groups by feed type and trained a MOFA model on each using the same parameters as described for the full model.

After each model was trained, we used the function correlate_factors_with_covariates in the MOFA2 package to identify factors that were significantly correlated (alpha = 0.05) with cestode detection, size class, and feed type, using Pearson correlation coefficients and associated *P* values (adjusted for multiple hypothesis testing). We focused on factors that significantly (adjusted *P* < 0.05) correlated with cestode detection and/or size class and those that explained >1% of the variation in at least two -omic data sets. However, the full results of the MOFA models can be found on the study’s GitHub (https://github.com/jcbrealey/Brealey_etal_salmon_multiomics). We report all features (MAG variables, metabolites, or genes) with absolute weights of >0.2 as significantly contributing to these factors. We compared the results of the full model to each model based on feed type and labeled a feature association as “robust” if it significantly contributed to a factor in both feed type models that was associated with the same fish variable (cestode detection or size class) in the full model. For visualization, MAG features were annotated with their assigned genus; metabolite features were annotated by metabolite class; and gene features were annotated by keywords in their associated GO terms or gene names (script available at https://github.com/jcbrealey/Brealey_etal_salmon_multiomics).

### MAG phylogenetics and comparative genomics

Phylogenetics and comparasite genomics for the *Mycoplasma* and Vibrionaceae MAGs were performed with the Anvi’o platform (124). Related Mycoplasmataceae and Vibrionaceae reference species genomes (Table S11) were annotated with Anvi’o using the Pfam (130) and KEGG Orthologs databases (131), as described for each MAG above. Pangenome profiles were generated in Anvi’o for the Mycoplasmataceae and Vibrionaceae genomes separately, including the identification of gene clusters by alignment with DIAMOND (169) in “fast” mode, elimination of weak alignments with the minbit heuristic (170), and clustering with MCL (171). Presence and completeness of KEGG metabolic pathways were estimated with the anvi-estimate-metabolism function. Amino acid sequences of single-copy bacterial ribosomal gene clusters (the Bacteria_71 collection in Anvi’o) were extracted, concatenated, and constructed into a phylogenomic tree using the Anvi’o implementation of FastTree (172). FigTree v.1.4.4 (http://tree.bio.ed.ac.uk/software/figtree/) was used to visualize the resulting tree and reroot it using the outgroup, *Ureaplasma* for Mycoplasmataceae and *Vibrio* for Vibrionaceae. Pairwise ANI was estimated using the Anvi’o implementation of PyANI (173).

For functional analysis, a literature search was performed to compile a list of genes known to be important for colonization, survival, and virulence in terrestrial and marine hosts of Mycoplasmataceae and Vibrionaceae (41, 45, 49, 52, 174–176). Where possible, KEGG pathways and KEGG ortholog and Pfam annotation of gene clusters were searched for these gene names or gene symbols. For genes that were less well characterized (e.g., adhesins lacking formal names), we downloaded reference sequences of these genes and compared them to the sequences of our pangenome gene clusters using blastp v.2.13 (177). A gene was defined as present in our pangenome if it had a corresponding KEGG or Pfam annotation, or if ≥50% of the reference sequence was aligned to a gene cluster with an *E* value of <0.01. A complete list of genes and accessions are reported in Table S12. Only those present in at least one MAG are shown in Fig. 5.

## Data Availability

Raw metagenomic, metabarcoding, genomic, and transcriptomic sequencing data are available at the European Nucleotide archive (ENA) under project PRJEB64334. Individual metadata are available as biosamples under the same project. All sample ENA accessions are provided in Data S6. Assembled metagenome-assembled genome sequences are available as analysis files under the same ENA project, with accession numberss provided in Table S2. Metabolomic data are available at MassIVE under accession number MSV000093872 and at MetaboLights under accession number MTBLS8575. A record of all statistical analyses is included in File S1. Original R scripts and feature abundance tables for each -omics data set are available in GitHub.

## SUPPLEMENTAL MATERIAL

Supplemental Figures msystems.01043-23-s0001.pdf Figures S1-S11.

Supplemental Data msystems.01043-23-s0002.xlsx Data S1-S6.

Supplemental Tables msystems.01043-23-s0003.xlsx Tables S1-S12.

File S1 msystems.01043-23-s0004.html Record of all statistical analyses.

ASM does not own the copyrights to Supplemental Material that may be linked to, or accessed through, an article. The authors have granted ASM a non-exclusive, world-wide license to publish the Supplemental Material files. Please contact the corresponding author directly for reuse.
