# Supplementary material for: Host–gut microbiota interactions shape parasite infections in farmed Atlantic salmon
Source: mSystems. 2024 Jan 31;9(2):e01043-23. doi: 10.1128/msystems.01043-23 (PMC10886447; doi:10.1128/msystems.01043-23)
Supplement: File S1 — Record of all statistical analyses. [file msystems.01043-23-s0004.html]

HoloFish multi-omics analysis


# HoloFish multi-omics analysis

#### Jaelle Brealey

#### December 01, 2023

- Analysis of HoloFish
  multi-omics project
  - Functions
  - Datasets
  - Explore
    metadata
    - Figure 1
  - Fatty
    acid profiles
    - Supp figure:
      PCA
  - 16S
    metabarcoding
    - Supp
      figure: heatmap
    - Supp figure:
      Venn
  - MetaG
    MAG evaluation
    - MAG
      catalogue
    - Extraction negatives
    - MAG vs 16S
      data
  - MetaG community analysis
    - Ordination
    - Microbime phenotypes
    - Supp figure ordination
    - Figure 2
    - Alpha
      diversity
    - Differential abundance
      testing
    - Differential detection
      testing
  - MAG functional analysis
    - Mycoplasmataceae
    - Vibrionaceae
  - HostG
    - PCA of population
      structure
    - GWAS results
  - HostRNA
    - Ordination
    - Figure 3D
    - Figure 3
      composite
    - Differential expression
    - QC
  - Metabolomics
    - Ordination
  - Multi-omics integration
    - Combined
      model
    - Feed1 model
    - Feed2 model
    - Figure 4
    - MOFA Supp
      data
- END

# Analysis of HoloFish multi-omics project

R analysis of main results from Brealey *et al.* Host-gut
microbiota interactions shape parasite infections in farmed Atlantic
salmon. Currently available as a preprint: https://doi.org/10.1101/2023.07.20.549827.

Note, cestode and tapeworm are used interchangeably throughout.

## Functions

```
set.seed(6854)

# Functions
run_maaslin2 <- function(ps, out_name, f_eff, r_eff = NA, reference = NA, n = "none", t = "none"){
  # run Maaslin2 for differential abundance testing
  if(file.exists(paste0(out_name,"/all_results.tsv"))){
    maas.out <- read.delim(paste0(out_name,"/all_results.tsv"))
  } else {
    if(is.na(r_eff) & is.na(reference)){
      maas.out <- Maaslin2(
        input_data = data.frame(otu_table(ps)),
        input_metadata = data.frame(sample_data(ps)),
        fixed_effects = f_eff,
        output = out_name,
        normalization = n, transform = t
      )
    } else if(!is.na(r_eff) & is.na(reference)) {
      maas.out <- Maaslin2(
        input_data = data.frame(otu_table(ps)),
        input_metadata = data.frame(sample_data(ps)),
        fixed_effects = f_eff,
        random_effects = r_eff,
        output = out_name,
        normalization = n, transform = t
      )
    } else if(is.na(r_eff) & !is.na(reference)){
      maas.out <- Maaslin2(
        input_data = data.frame(otu_table(ps)),
        input_metadata = data.frame(sample_data(ps)),
        fixed_effects = f_eff,
        reference = reference,
        output = out_name,
        normalization = n, transform = t
      )
    } else {
      maas.out <- Maaslin2(
        input_data = data.frame(otu_table(ps)),
        input_metadata = data.frame(sample_data(ps)),
        fixed_effects = f_eff,
        random_effects = r_eff,
        reference = reference,
        output = out_name,
        normalization = n, transform = t
      )
    }
    maas.out <- maas.out$results
  }
  maas.out <- maas.out[order(maas.out$metadata, maas.out$feature, maas.out$qval),]
  return(maas.out)
}


ps_ordination_f <- function(df, method, dist, k.range, rds.name){
  # v1 performs ordination using phyloseq command for given k range
  # v2 performs ordination using vegan command for given k range
  # returns the lowest k for which the model converged
  # only tested with NMDS so far
  # only run once - if file exists, save and import
  if(file.exists(rds.name)){
    ord.out <- readRDS(rds.name)
    return(ord.out)
  } else {
    for (k in k.range){
      if(class(df) == "dist"){
      # assume vegan
      ord <- metaMDS(df, k = k, try = 20, trymax = 100, maxit = 2000)
      } else if(class(df) == "phyloseq") {
        # phyloseq
        ord <- ordinate(df, method = method, distance = dist, k = k, try = 20, trymax = 100, maxit = 2000)
      } else {
        stop("Incorrect input format.")
      }
      if(ord$converged){
        ord.out <- list(ord = ord, k = k)
        saveRDS(ord.out, file = rds.name)
        return(ord.out)
      }
    }
    stop("No convergence for provided k values.")
  }
}

process_pheno_data_for_gwas_f <- function(df, sample_ids, sample_order_file, out_file, abund_thresh = 0, qtrans = TRUE){
  # prepare pheno data for parsing to GEMMA
  # 1 column per pheno variable, with samples in the same order as the angsd beagle file
  # df - all pheno variables to be included (no sample names)
  # sample_ids - sample names in same order as df (doesn't have to be in same order as sample_order_file)
  if(!file.exists(out_file)){
    # read in sample order
    sample.order <- read.delim(sample_order_file, header = F, col.names = "Sample")
    # save variable names
    df.names <- names(df)
    # set to NA if abundance <= threshold
    df[df <= abund_thresh] <- NA

    if(qtrans){
      # apply INT transformation
      df <- as.data.frame(apply(df, MARGIN = 2, FUN = qtrans))
    }
    df$Sample <- sample_ids
    out <- merge(sample.order, df, by = "Sample", all.x = T, all.y = F)
    out <- out[match(sample.order$Sample, out$Sample),]
    write.table(out[,df.names], out_file, sep = "\t", row.names = F, quote = F, col.names = T)
    # saving col names, will need to skip first line before parsing to GEMMA
    
    # print number of samples remaining after filtering
    print(colSums(!is.na(out[,df.names])))
  }
}

process_cov_data_for_gwas_f <- function(df, sample_ids, sample_order_file, out_file){
  # prepare covariate data for parsing to GEMMA
  # 1 column per cov variable, with samples in the same order as the angsd beagle file
  # first column is the intercept (all 1s)
  # df - all cov variables to be included (no sample names); all cov variables should be transformed already
  # sample_ids - sample names in same order as df (doesn't have to be in same order as sample_order_file)
  if(!file.exists(out_file)){
    # read in sample order
    sample.order <- read.delim(sample_order_file, header = F, col.names = "Sample")
    
    # add intercept for GEMMA
    df$intercept <- 1
    
    # save variable names, with intercept first
    df.names <-  names(df)[c(length(names(df)),2:length(names(df))-1)]
    
    df$Sample <- sample_ids
    out <- merge(sample.order, df, by = "Sample", all.x = T, all.y = F)
    
    out <- out[match(sample.order$Sample, out$Sample),]
    write.table(out[,df.names], out_file, sep = "\t", row.names = F, quote = F, col.names = T)
    # saving col names, will need to skip first line before parsing to GEMMA
  }
}

# transformation suggested by Anders: INT
qtrans <-
  function(x){
    k<-!is.na(x)
    ran<-rank(x[k])
    y<-qnorm((1:sum(k)-0.5)/sum(k))
    x[k]<-y[ran]
    x
  }

# Run DESeq and process results
run_deseq_f <- function(ct.df, meta.df, design.df, sig.level, output.ext){
  if(file.exists(paste0(output.ext,"_DEGs.csv"))){
    res.tab <- read.delim(paste0(output.ext,"_DEGs.csv"), sep = ",")
    res.dds <- readRDS(paste0(output.ext,"_DEdds.rds"))
  } else {
    if(!file.exists(paste0(output.ext,"_DEdds.rds"))){
      res.dds <- DESeqDataSetFromMatrix(countData = ct.df[,match(row.names(design.df),names(ct.df))], 
                                        colData = meta.df[match(row.names(design.df),row.names(meta.df)),], 
                                        design = design.df)
      res.dds <- estimateSizeFactors(res.dds)
      res.dds <- DESeq(res.dds)
      saveRDS(res.dds, paste0(output.ext,"_DEdds.rds"))
    }
    res.dds <- readRDS(paste0(output.ext,"_DEdds.rds"))
    writeLines("gene,baseMean,log2FoldChange,lfcSE,stat,pvalue,padj,phenotype", paste0(output.ext,"_DEGs.csv"))
    for(i in colnames(design.df)[-1]){
      res.tab <- results(res.dds, name = i, alpha = sig.level)
      res.tab <- as.data.frame(res.tab[which(res.tab$padj < sig.level),])
      if(nrow(res.tab) > 0){
        res.tab$phenotype <- i
        write.table(res.tab, paste0(output.ext,"_DEGs.csv"), row.names = TRUE, quote = FALSE, col.names = FALSE, sep = ",", append = TRUE)
      } else {
        next
      }
    }
    res.tab <- read.delim(paste0(output.ext,"_DEGs.csv"), sep = ",")
  }
  # get lists of DEGs at significance level
  degs <- list()
  print(paste("No. of genes significantly associated at p <",sig.level,"with each variable:"))
  for(i in unique(res.tab$phenotype)){
    t <- list(res.tab[which(res.tab$phenotype == i & res.tab$padj < sig.level),"gene"])
    names(t) <- i
    print(paste(i,"-", length(t[[1]])))
    degs <- append(degs, t)
  }
  return(list(results.tab=res.tab, deg.list=degs, results.dds=res.dds))
}

run_mofa_default <- function(df.input, outfile, metadata, covar, view.distrib, num_factors = 10){
  if(file.exists(paste0(outfile,".hdf5"))){
    # Load existing MOFA model
    mofa.obj.trained <- load_model(paste0(outfile,".hdf5"))
    mofa.corr <- read.csv(paste0(outfile,"_covariates_corr.csv"))
    mofa.weights <- read.csv(paste0(outfile,"_feature_weights.csv"))
    mofa.samples <- read.csv(paste0(outfile,"_sample_weights.csv"))
    
  } else {
    # run MOFA model
    # Set up
    mofa.obj <- create_mofa(df.input)
    
    # Define parameters
    opts.data <- get_default_data_options(mofa.obj)
    opts.data$scale_views <- TRUE # scale to make sure different views have similar variances
    opts.model <- get_default_model_options(mofa.obj)
    opts.model$num_factors <- num_factors
    # correct view model distributions
    # note views are sorted in alphabetic order by name, so distributions need to match
    if(length(view.distrib) != length(names(opts.model$likelihoods))){
      stop("No. of view distributions do not match number of views")
    }
    opts.model$likelihoods <- sapply(seq(1,length(view.distrib)), function(i) { 
      opts.model$likelihoods[i] <- view.distrib[i]
    })
    opts.train <- get_default_training_options(mofa.obj) #note for final model, switch convergence mode from 'fast' to 'medium' or 'slow'
    
    # Build and train model
    
    mofa.obj <- prepare_mofa(
      object = mofa.obj,
      data_options = opts.data,
      model_options = opts.model,
      training_options = opts.train
    )
    
    mofa.obj.trained <- run_mofa(mofa.obj, paste0(outfile,".hdf5"), use_basilisk = T)
    
    # Add metadata to MOFA object
    samples_metadata(mofa.obj.trained) <- metadata[samples_names(mofa.obj.trained)[[1]],]
    
    # save variances
    write.csv(get_variance_explained(mofa.obj.trained)$r2_total$single_group,
              paste0(outfile,"_variance_total.csv"),
              row.names = T, quote = F)
    write.csv(get_variance_explained(mofa.obj.trained)$r2_per_factor$single_group,
              paste0(outfile,"_variance_per_factor.csv"),
              row.names = T, quote = F)
    
    # save correlations with covariates
    mofa.corr <- merge(
      x = melt(correlate_factors_with_covariates(mofa.obj.trained,
                                                 covariates = covar,
                                                 plot="r", return_data = T), 
               varnames = c("Factor","Phenotype"), value.name = "cor.r"),
      y = melt(correlate_factors_with_covariates(mofa.obj.trained,
                                                 covariates = covar,
                                                 plot="log_pval", return_data = T), 
               varnames = c("Factor","Phenotype"), value.name = "cor.logp"),
      by = c("Factor","Phenotype")
    )
    mofa.corr$cor.p <- 10^(-mofa.corr$cor.logp)
    
    write.csv(mofa.corr, paste0(outfile,"_covariates_corr.csv"),
              row.names = F, quote = F)
    
    # save feature weights
    mofa.weights <- get_weights(
      mofa.obj.trained,
      views = "all",
      factors = "all",
      as.data.frame = TRUE,
      scale = F
    )
    write.csv(mofa.weights, paste0(outfile,"_feature_weights.csv"),
              row.names = F, quote = F)
    
    # save factor values for samples
    mofa.samples <- get_factors(mofa.obj.trained, as.data.frame = T)
    write.csv(mofa.samples, paste0(outfile,"_sample_weights.csv"),
              row.names = F, quote = F)    
  }
  
  # return list of output
  return(list(trained.obj=mofa.obj.trained, corr=mofa.corr, feature.weights=mofa.weights, sample.weights=mofa.samples))
}

get_feature_abundance_meta <- function(abund.mat, feature.ids, meta, sample.var = "Sample", sample.ids = NULL){
  # extract out abundance values for a specific set of genes/samples from an abundance matrix and return with supplied metadata in long format for plots, etc
  # abundance matrix should be features as rows, samples as columns
  # if sample.ids is NULL, use all
  if(is.null(sample.ids)){
    sample.ids <- colnames(abund.mat)
  }
  if(is.matrix(abund.mat)){
    if(length(feature.ids) == 1) {
      fid <- feature.ids
      df <- data.frame(Sample=names(abund.mat[fid,sample.ids]),
                       feature=fid,
                       abundance=abund.mat[fid,sample.ids])
      names(df)[1] <- sample.var
      df.long <- merge(df, meta, by = sample.var, all.x = T, all.y = F)
    } else {
      df <- abund.mat[feature.ids,sample.ids]
      df.long <- reshape2::melt(df, varnames = c("feature",sample.var), value.name = "abundance")
      df.long <- merge(df.long, meta, by = sample.var, all.x = T, all.y = F)
    }
    return(df.long)
  } else {
    stop("abund.mat is not a matrix")
  }
}

get_annotation_info_f <- function(ah.db, gene.symbols){
  # take a list of gene symbols from ref genome annotation, return the GO terms associated with those symbols
  # requires AnnotationDbi for organism
  anno <- AnnotationDbi::select(ah.db, keys=gene.symbols, columns = c("SYMBOL","ENTREZID","GENENAME","GO","ONTOLOGY"), keytype = "SYMBOL")
  anno$GO.term <- sapply(anno$GO, function(x){
    if(!is.na(x)){
      go.hit <- mget(x, GO.db::GOTERM, ifnotfound = NA)
      if(!is.na(go.hit)){
        go.hit2 <- grep(x,unlist(go.hit), value = T)
        go.hit <- grep("^ Term",strsplit(go.hit2[[x]], split = ",")[[1]], value = T)
      }
      go.hit
    } else {
      NA
    }
  })
  return(anno)
}

# annotate MOFA features with useful information (e.g. MAG taxonomy, gene names, metabolite category, etc)
annotate_mofa_f <- function(mofa.weights, view, factors, threshold = 0.2){
  # transcriptome
  if(view == "HostRNA"){
    df <- data.frame(
      feature=as.character(mofa.weights[which(mofa.weights$view == "HostRNA" & mofa.weights$factor %in% factors),"feature"]),
      value=as.numeric(mofa.weights[which(mofa.weights$view == "HostRNA" & mofa.weights$factor %in% factors),"value"]),
      factor=mofa.weights[which(mofa.weights$view == "HostRNA" & mofa.weights$factor %in% factors),"factor"]
    )
    
    # get gene names to help with classification
    df$gene.name <- sapply(seq(1,nrow(df)), function(i){
      x <- df[i,"feature"]
      f <- df[i,"factor"]
      sym <- strsplit(x, split = "\\+")[[1]][2]
      if(abs(df[i,"value"]) < threshold){
        return("")
      }
      if(grepl("rna",sym)){
        return("")
      }
      # get gene name from GO annotations
      tryCatch(
        {
          anno <- get_annotation_info_f(SsalOrg, sym)
        }, error=function(e){ 
          cat("ERROR")
          return("") 
        }
      )
      if(length(anno) == 0){
        return("")
      } else {
        return(paste(unique(anno[,"GENENAME"]),collapse = ";"))
      }
    })
    
    # gene annotation for all
    df$anno.figure <- sapply(seq(1,nrow(df)), function(i){
      x <- df[i,"feature"]
      f <- df[i,"factor"]
      sym <- strsplit(x, split = "\\+")[[1]][2]
      gname <- df[i,"gene.name"]
      
      if(abs(df[i,"value"]) < threshold){
        return("")
      }
      
      if(grepl("rna",sym)){
        return("")
      }
      # pre-define some genes of interest (taurine related and antimicrobial lectins that don't have GO terms)
      if(sym %in% c("LOC106576775","LOC106593002","LOC106580183","gadl1")){
        return("taurine")
      }
      if(sym %in% c("LOC106578378","LOC106563633","scdb","faxdc2")){
        return("lipid")
      }
      if(sym %in% c("fel","LOC106581127","LOC106602162","LOC106570159","LOC106592508","LOC100136446",
                    "LOC106580088","LOC106576106",
                    "LOC106601915","LOC106606429","LOC100196094","LOC106612711","LOC106611589","LOC106587204",
                    "LOC106568508","LOC106607932","LOC106602161","mpx")){
        return("immune")
      }
      if(sym %in% c("1433b","dusp1","LOC106610225","LOC106610223","LOC106609495","LOC106576427")){
        return("signaling")
      }
      if(sym %in% c("LOC106590176")){
        # unclear function as uncharacterised in new annotation
        return("")
      }  
      if(sym %in% c("agr2","LOC100136469","LOC106560454","muc3a")){
        #return("mucin") --> probably not that interesting
        return("")
      }
      # pre-define some gene names of interest
      if(grepl("hemoglobin", gname, ignore.case = T)){
        return("heme")
      }
      if(grepl("ice-structuring protein", gname, ignore.case = T)){
        #return("antifreeze") --> probably not that interesting
        return("")
      }
      if(grepl("thrombospondin", gname, ignore.case = T)){
        return("immune")
      }
      if(grepl("apolipoprotein", gname, ignore.case = T)){
        # mostly lipid-related functions, but has a major role interaction with endotoxins like LPS
        return("apolipoprotein")
      }
      if(grepl("tensin", gname, ignore.case = T)){
        return("signaling")
      }
      if(grepl("actin", gname, ignore.case = T)){
        return("ECM")
      }
      if(grepl("interferon", gname, ignore.case = T)){
        return("immune")
      }
      if(grepl("interleukin", gname, ignore.case = T)){
        return("immune")
      }
      
      # otherwise, text mine the GO annotations
      tryCatch(
        {
          anno <- get_annotation_info_f(SsalOrg, sym)
        }, error=function(e){ 
          cat("ERROR")
          return("") 
        }
      )
      if(length(anno) == 0){
        return("")
      } else {
        terms <- gsub('[\"]', '', anno$GO.term)
        terms <- gsub(" Term = ","",terms)
        terms <- paste(terms,collapse = " ")
        
        # immune-related
        i.keywords <- c("defense","bacteri","apopto","death","cytolysis","leuk","immun","complement","interspecies",
                        "ladderlectin","interferon","biotic","inflammatory")
        i.ct <- stringr::str_count(terms, paste(i.keywords,collapse = "|"))
        # lipid-related
        l.keywords <- c("lipoprotein","lipid","sterol","hydroxy","fatty acid")
        l.ct <- stringr::str_count(terms, paste(l.keywords,collapse = "|"))
        # heme/iron-related
        h.keywords <- c("heme","porphyrin","tetrapyrrole","iron")
        h.ct <- stringr::str_count(terms, paste(h.keywords,collapse = "|"))
        # ECM-related
        e.keywords <- c("collagen","actin","actomyosin","extracellular matrix","cell adhesion")
        e.ct <- stringr::str_count(terms, paste(e.keywords,collapse = "|"))
        # signal transduction
        s.keywords <- c("signal","integrin","Notch","JAK-STAT")
        s.ct <- stringr::str_count(terms, paste(s.keywords,collapse = "|"))
        # taurine related
        t.keywords <- c("taurine")
        t.ct <- stringr::str_count(terms, paste(t.keywords,collapse = "|"))
        res.cts <- data.frame(anno = c("immune","lipid","heme","ECM","signaling","taurine"), 
                              ct = c(i.ct,l.ct,h.ct,e.ct,s.ct,t.ct))
        if(sum(res.cts$ct) == 0){
          return("")
        } else {
          return(paste(res.cts[which(res.cts$ct == max(res.cts$ct)),"anno"],collapse = ";"))
        }
      }
    })
  }
  ## metabolome
  if(view == "mbol"){
    df <- data.frame(
      feature=as.character(mofa.weights[which(mofa.weights$view == "mbol" & mofa.weights$factor %in% factors),"feature"]),
      value=as.numeric(mofa.weights[which(mofa.weights$view == "mbol" & mofa.weights$factor %in% factors),"value"]),
      factor=mofa.weights[which(mofa.weights$view == "mbol" & mofa.weights$factor %in% factors),"factor"]
    )
    # use mbol annotation (mbol.chemdata)
    df <- merge(df, mbol.chemdata[,c(1:6,8)], by.x = "feature", by.y = "FT_ID", all.x = T, all.y = F)
    df$anno.figure <- sapply(seq(1,nrow(df)), function(i){
      x <- df[i,"feature"]
      f <- df[i,"factor"]
      if(abs(df[i,"value"]) < threshold){
        return("")
      }
      else {
        a <- mbol.chemdata[which(mbol.chemdata$FT_ID == x), "R4.level5"]
        if(a %in% c("Glycinated bile acids and derivatives","Hydroxy bile acids, alcohols and derivatives")){
          return("bile.acids")
        } else if(a %in% c("14-hydroxysteroids","3-hydroxysteroids")){
          return("hydroxysteroids")
        } else if(a %in% c("Amino acids and derivatives")){
          return("amino.acids")
        } else if(a %in% c("Acyl carnitines")){
          return("acyl.carnitines")
        } else {
          return("")
        }
      }
    })
  }
  ## metagenome
  if(view == "MetaG"){
    df <- data.frame(
      feature=as.character(mofa.weights[which(mofa.weights$view == "MetaG" & mofa.weights$factor %in% factors),"feature"]),
      value=as.numeric(mofa.weights[which(mofa.weights$view == "MetaG" & mofa.weights$factor %in% factors),"value"]),
      factor=mofa.weights[which(mofa.weights$view == "MetaG" & mofa.weights$factor %in% factors),"factor"]
    ) 
    df$anno.figure <- sapply(seq(1,nrow(df)), function(i){
      x <- df[i,"feature"]
      f <- df[i,"factor"]
      if(abs(df[i,"value"]) > threshold){
        if(grepl("ali", x, ignore.case = T)){
          "Aliivibrio"
        } else if(grepl("photo", x, ignore.case = T)){
          "Photobacterium"
        } else if(grepl("myco", x, ignore.case = T)){
          "mycoplasma"
        } else if(grepl("Msal", x, ignore.case = T)){
          "M.salmoninae"
        } else {
          ""
        }
      } else {
        ""
      }
    })
  }
  return(df)
}

gg_color_hue <- function(n) {
  # emulate ggplot2 default colors
  hues = seq(15, 375, length = n + 1)
  hcl(h = hues, l = 65, c = 100)[1:n]
}
```

## Datasets

- fish phenotypes (size class, tapeworm infection status, etc)
- fatty acid muscle profiles
- shotgun metagenomics from gut content (MetaG)
- 16S metabarcoding from gut content, gut mucosa and feed pellets
  (16S)
- whole genome resequencing from gill tissue (HostG)
- mRNA transcriptomics from gut epithelia (HostT)
- metabolomics from gut content (mbol)

## Explore metadata

```
# Load and process data
meta <- read.csv("data/Supp_table_fish_metadata_230531.csv")
row.names(meta) <- meta$Fish.ID
meta$Size.class <- factor(meta$Size.class,levels = c("Small","Medium","Large"), ordered = T)
meta$Tapeworm <- factor(meta$Tapeworm, levels = c(0,1,2,3), ordered = T)

metavar.fish <- c("Gutted.Weight.kg","Tapeworm","Tapeworm.bin","Feed.Type","Size.class","Salmofan.Score")
metavar.exp <- c("Sampling.Date","Team")

fish.ids <- meta$Fish.ID

metag.samples <- meta[which(meta$Metagenome),"Fish.ID"]
hostg.samples <- meta[which(meta$HostGenome),"Fish.ID"]
rna.samples <- meta[which(meta$HostTranscriptome),"Fish.ID"]
mbol.samples <- meta[which(meta$Metabolome),"Fish.ID"]


## plotting colours 
anno_colours <- list(
  Size.class=c(Small=gg_color_hue(3)[3], Medium=gg_color_hue(3)[2], Large=gg_color_hue(3)[1]),
  Feed.Type=c(Feed1="#ffa200", Feed2="#b700ff"),
  Tapeworm=c(`0`="#440154FF", `1`="#31688EFF", `2`="#35B779FF", `3`="#FDE725FF"),
  Tapeworm.bin=c(`FALSE`="#440154FF", `TRUE`="#FDE725FF"),
  MAG.det=c(`FALSE`="grey70",`TRUE`="grey20"),
  Mbol.batch=c(Batch_1="#F8766D", Batch_2="#7CAE00", Batch_3="#00BFC4", Batch_4="#C77CFF"),
  Size.tapeworm=c(Large.TW0="#a80c02", Large.TW23="#fabfbb", Small.TW0="#023e9e", Small.TW23="#b3cffc")
)
```

### Figure 1

```
# histogram of size distribution
fig1a <-
ggplot(meta, aes(Gutted.Weight.kg, fill = Size.class))+
  geom_histogram(binwidth = 0.4, color = "black")+
  scale_fill_manual(values = anno_colours$Size.class)+
  scale_x_continuous(limits = c(0,8))+
  labs(x = "Gutted weight (kg)", y = "No. of individuals", fill = "Size class")+
  theme_classic()+
  theme(axis.text = element_text(color = "black", size = 12), axis.title = element_text(size = 13),
        legend.position = "right", legend.text = element_text(size = 12), legend.title = element_text(size = 12))

# cestode index vs gutted weight
fig1b <-
ggplot(meta, aes(Tapeworm, Gutted.Weight.kg))+
  geom_violin(aes(fill = Tapeworm), alpha = 0.25)+
  geom_point(aes(fill = Tapeworm), shape = 21, color = "black", size = 3, position = position_jitterdodge(jitter.width = 0.25))+
  stat_summary(fun = "mean", geom = "point", color = "grey50", fill = "white", size = 3, shape = 21)+
  scale_color_manual(values = anno_colours$Tapeworm)+
  scale_fill_manual(values = anno_colours$Tapeworm)+
  scale_y_continuous(limits = c(0,8))+
  labs(x = "Cestode index", y = "Gutted weight (kg)")+
  theme_classic()+
  theme(axis.text = element_text(color = "black", size = 12), legend.position = "none",
        axis.title = element_text(size = 13))

# data overview
data.overview <- rbind.data.frame(
  cbind.data.frame(Sample=metag.samples, Dataset="Metagenome"),
  cbind.data.frame(Sample=hostg.samples, Dataset="Host\nGenome"),
  cbind.data.frame(Sample=rna.samples, Dataset="Host\nTranscriptome"),
  cbind.data.frame(Sample=mbol.samples, Dataset="Metabolome")
)
data.overview$value <- as.character("1")
data.overview$Dataset <- factor(data.overview$Dataset, levels = c("Host\nGenome","Host\nTranscriptome","Metabolome","Metagenome"))
data.overview$no.dataset.per.sample <- sapply(data.overview$Sample, function(x){
  nrow(data.overview[which(data.overview$Sample == x),])
})
data.overview$Sample <- factor(data.overview$Sample,
                                    levels = unique(data.overview[order(data.overview$no.dataset.per.sample, decreasing = T),"Sample"]))

fig1c <-
ggplot(data.overview, aes(Sample, Dataset, fill = Dataset))+
  geom_tile()+
  scale_fill_manual(values = c("#FDD0A2","#FDAE6B","#FD8D3C","#E6550D"))+
  labs(x = "Individual")+
  theme_bw()+
  theme(axis.text = element_text(size = 12, colour = "black"), axis.text.x = element_blank(),
        axis.title.y = element_blank(), axis.ticks.x = element_blank(),
        axis.title.x = element_text(size = 13),
        legend.position = "none")
```

```
plot_grid(fig1a + theme(legend.position = c(0.865,0.57)),fig1b,fig1c,
          align = "h", axis = "tblr", nrow = 1, rel_widths = c(1,1,1),
          labels = c("A","B","C"))
```

```
ggsave("figures/Figure_data_summary.png", units = "in", dpi = 800, width = 12, height = 4)
```

**Fig. 1. Overview of fish metadata and -omic
datasets.** (**A**) Distribution of lifetime growth
of the 460 sampled salmon, measured as gutted weight (kg) at harvest.
Individuals were classified into three discrete size classes as
indicated by the coloured bars, with the aim to have approximately equal
numbers of individuals in each class. (**B**) Gutted weight
at harvest decreases as cestode infection levels increase. Violin plots
show the distribution of salmon weights, white circles indicate the
mean. (**C**) Gut metagenomes, gut metabolomes, host
transcriptomes and host genomes were successfully generated for a subset
of individuals. Grey bars indicate that an -omic dataset is missing for
that individual. Individuals are ordered by the number of -omic datasets
with data available.

Association between gutted weight and tapeworm index:

```
summary(lm(Gutted.Weight.kg ~ Tapeworm + Feed.Type, meta))
```

```
## 
## Call:
## lm(formula = Gutted.Weight.kg ~ Tapeworm + Feed.Type, data = meta)
## 
## Residuals:
##     Min      1Q  Median      3Q     Max 
## -2.6041 -1.0463 -0.0151  0.9712  3.9390 
## 
## Coefficients:
##                Estimate Std. Error t value Pr(>|t|)    
## (Intercept)     3.30229    0.08793  37.554  < 2e-16 ***
## Tapeworm.L     -0.93364    0.17235  -5.417 9.83e-08 ***
## Tapeworm.Q     -0.15605    0.14349  -1.088  0.27737    
## Tapeworm.C     -0.31693    0.11067  -2.864  0.00438 ** 
## Feed.TypeFeed2 -0.03048    0.12354  -0.247  0.80524    
## ---
## Signif. codes:  0 '***' 0.001 '**' 0.01 '*' 0.05 '.' 0.1 ' ' 1
## 
## Residual standard error: 1.294 on 455 degrees of freedom
## Multiple R-squared:  0.06515,    Adjusted R-squared:  0.05693 
## F-statistic: 7.927 on 4 and 455 DF,  p-value: 3.492e-06
```

Association between tapeworm detection and feed type:

```
prop.table(table(meta[,c("Tapeworm.bin", "Feed.Type")]), margin = 2)
```

```
##             Feed.Type
## Tapeworm.bin     Feed1     Feed2
##        FALSE 0.1560000 0.2190476
##        TRUE  0.8440000 0.7809524
```

```
fisher.test(meta$Tapeworm.bin, meta$Feed.Type)
```

```
## 
##  Fisher's Exact Test for Count Data
## 
## data:  meta$Tapeworm.bin and meta$Feed.Type
## p-value = 0.09183
## alternative hypothesis: true odds ratio is not equal to 1
## 95 percent confidence interval:
##  0.3986806 1.0867805
## sample estimates:
## odds ratio 
##  0.6595911
```

## Fatty acid profiles

Load and process fatty acid profiles generated from salmon
muscle.

```
fa.fish.raw <- read.delim("data/FattyAcid/20190521-Fattyacidprofil_fish_filet-HI.csv", sep = ";", as.is = T)
fa.pellet.raw <- read.delim("data/FattyAcid/20190521-Fattyacidprofil_feed_pellets-HI.csv", sep = ",", as.is = T)

# clean up names etc
names(fa.fish.raw)[c(3,7,8,9,11,16)] <- c("Fish.nr","Analyte","Result.raw0","Result.num","Unit.type","Result.raw1")
fa.fish.raw$Run <- sapply(as.character(fa.fish.raw$Lims.number), function(s) {
  strsplit(s, split = "/")[[1]][1]
})
fa.fish.raw$Fish.ID <- sapply(fa.fish.raw$Fish.nr, function(i){
  if(i < 10){
    paste0("F00",i)
  } else if(i < 100){
    paste0("F0",i)
  } else {
    paste0("F",i)
  }
})

# Fish F453 was excluded from the study because we have no successful -omics data (just low-coverage RNA)
fa.fish.raw <- fa.fish.raw[which(fa.fish.raw$Fish.ID != "F453"),]

names(fa.pellet.raw)[1] <- "org.order"
fa.pellet.raw$Run <- sapply(as.character(fa.pellet.raw$Lims.number), function(s) {
  strsplit(s, split = "/")[[1]][1]
})
fa.pellet.raw$Fish.ID <- fa.pellet.raw$Fish.nr

fa.both.raw <- rbind.data.frame(fa.fish.raw, fa.pellet.raw)

fa.both.raw$Analyte <- gsub(pattern = "24.00.00", replacement = "24:0", fa.both.raw$Analyte)
fa.both.raw$Analyte <- gsub(pattern = "^", replacement = "a", fa.both.raw$Analyte)
fa.both.raw$Analyte <- gsub(pattern = "%", replacement = "per", fa.both.raw$Analyte)
fa.both.raw$Analyte <- gsub(pattern = "aSum", replacement = "sum", fa.both.raw$Analyte)
fa.both.raw$Analyte <- gsub(pattern = "\\(", replacement = "", fa.both.raw$Analyte)
fa.both.raw$Analyte <- gsub(pattern = ")", replacement = "", fa.both.raw$Analyte)
fa.both.raw$Analyte <- gsub(pattern = " ", replacement = "_", fa.both.raw$Analyte)
fa.both.raw$Analyte <- gsub(pattern = "\\:", replacement = ".", fa.both.raw$Analyte)
fa.both.raw$Analyte <- gsub(pattern = "\\/", replacement = "R", fa.both.raw$Analyte)
fa.both.raw$Analyte <- gsub(pattern = "_\\+", replacement = "", fa.both.raw$Analyte)
fa.both.raw$Analyte <- gsub(pattern = "en-umettet", replacement = "monounsat", fa.both.raw$Analyte)
fa.both.raw$Analyte <- gsub(pattern = "fettsyrer", replacement = "allFA", fa.both.raw$Analyte)
fa.both.raw$Analyte <- gsub(pattern = "flerumettet", replacement = "polyunsat", fa.both.raw$Analyte)
fa.both.raw$Analyte <- gsub(pattern = "uidentifiserte", replacement = "unidentified", fa.both.raw$Analyte)
fa.both.raw$Analyte <- gsub(pattern = "identifiserte", replacement = "identified", fa.both.raw$Analyte)
fa.both.raw$Analyte <- gsub(pattern = "mettet", replacement = "saturated", fa.both.raw$Analyte)
fa.both.raw$Analyte <- gsub(pattern = "-", replacement = "", fa.both.raw$Analyte)
fa.both.raw$Analyte <- gsub(pattern = "00", replacement = "0", fa.both.raw$Analyte)
# unique(fa.both.raw$Analyte)

fa.both.raw[which(fa.both.raw$Unit.type == "PROSENT"),"Unit.type"] <- "PERCENT"
fa.both.raw[which(fa.both.raw$Unit.type == "NONE"),"Unit.type"] <- "RATIO"


analytes.mg <- unique(grep("sum",fa.both.raw[which(fa.both.raw$Unit.type == "MG_G_WW"), "Analyte"], value = T, invert = T))
analytes.per <- unique(grep("sum",fa.both.raw[which(fa.both.raw$Unit.type == "PERCENT"), "Analyte"], value = T, invert = T))

analytes.sum.per <- unique(grep("sum",fa.both.raw[which(fa.both.raw$Unit.type == "PERCENT"), "Analyte"], value = T))

# 2018-230 a pilot study, exclude
fa.fish.pilot <- fa.both.raw[which(fa.both.raw$Run == "2018-230"),]

# resplit fish and pellet
fa.holo <- fa.both.raw[which(fa.both.raw$Run != "2018-230" & fa.both.raw$Organ == "Filet"),]
fa.feed <- fa.both.raw[which(fa.both.raw$Run != "2018-230" & fa.both.raw$Organ == "Fish.feed"),]

# add fatty acid batch info to meta
meta$Fatty_Acid_Batch <- sapply(meta$Fish.ID, function(x){
  if(x %in% unique(fa.holo$Fish.ID)){
    unique(fa.holo[which(fa.holo$Fish.ID == x),"Run"])
  } else {
    NA
  }
})

## get short formats
# %
fa.per <- dcast(formula = Fish.ID ~ Analyte, data = fa.holo[which(fa.holo$Unit.type == "PERCENT"),], 
                     value.var = "Result.num")
row.names(fa.per) <- fa.per$Fish.ID

# fatty acids not present in samples (remove from analysis)
# names(which(colSums(fa.per[,2:ncol(fa.per)], na.rm = TRUE) == 0))
fa.per <- fa.per[,c("Fish.ID", names(which(colSums(fa.per[,2:ncol(fa.per)], na.rm = TRUE) > 0)))]

# mg
fa.mg <- dcast(formula = Fish.ID ~ Analyte, data = fa.holo[which(fa.holo$Unit.type == "MG_G_WW"),], 
               value.var = "Result.num")
row.names(fa.mg) <- fa.mg$Fish.ID
# names(which(colSums(fa.mg[,2:ncol(fa.mg)], na.rm = TRUE) == 0))
# "a06.0"   "a08.0"   "a12.0"   "a20.3n3"
fa.mg <- fa.mg[,c("Fish.ID", names(which(colSums(fa.mg[,2:ncol(fa.mg)], na.rm = TRUE) > 0)))]

# ratios
fa.ratio <- dcast(formula = Fish.ID ~ Analyte, data = fa.holo[which(fa.holo$Unit.type == "RATIO"),], 
                     value.var = "Result.num")
row.names(fa.ratio) <- fa.ratio$Fish.ID
```

### Supp figure: PCA

```
# PCA on % analytes (not sums) including pellets
# change NAs to 0s (could use pseudozero of 0.01 if needed as original data lists NAs as <0.1 %)
per.pca.df <- fa.per[,which(names(fa.per) %in% analytes.per)]
per.pca.df[is.na(per.pca.df)] <- 0
per.pca.df$Fish.ID <- row.names(per.pca.df)
per.pca.df <- merge(per.pca.df, meta, by = "Fish.ID", all.x = T, all.y = F)
row.names(per.pca.df) <- per.pca.df$Fish.ID

per.pca1 <- prcomp(per.pca.df[,which(names(per.pca.df) %in% analytes.per)], scale. = T)

get_eigenvalue(per.pca1)[c("Dim.1","Dim.2","Dim.3"),]
```

```
##       eigenvalue variance.percent cumulative.variance.percent
## Dim.1  12.820954        31.270620                    31.27062
## Dim.2   4.646899        11.333901                    42.60452
## Dim.3   2.640271         6.439684                    49.04421
```

```
per.pca1.coord <- as.data.frame(get_pca_ind(per.pca1)$coord)
per.pca1.coord$Fish.ID <- row.names(per.pca1.coord)
per.pca1.coord <- merge(per.pca1.coord[,c("Fish.ID","Dim.1","Dim.2","Dim.3")], per.pca.df, by = "Fish.ID", all.x = TRUE)

fa.pc1 <- round(get_eigenvalue(per.pca1)["Dim.1","variance.percent"], digits = 1)
fa.pc2 <- round(get_eigenvalue(per.pca1)["Dim.2","variance.percent"], digits = 1)
fa.pc3 <- round(get_eigenvalue(per.pca1)["Dim.3","variance.percent"], digits = 1)

fa.p1 <-
ggplot()+
  geom_vline(xintercept = 0, linetype = 3, size = 1, color = "grey42") + geom_hline(yintercept = 0, linetype = 3, size = 1, color = "grey42")+
  geom_point(aes(x = Dim.1, y = Dim.2, shape = Feed.Type, color = Fatty_Acid_Batch), 
             data = per.pca1.coord, size = 3)+
  scale_x_continuous(limits = c(-9,9)) + scale_y_continuous(limits = c(-9,9))+
  coord_equal()+
  labs(x = paste0("PC1 (",fa.pc1,"%)"), y = paste0("PC2 (",fa.pc2,"%)"), color = "Fatty acid\nbatch", shape = "Feed type")+
  theme_bw() + theme(panel.grid = element_blank(), legend.position = "right",
                     axis.text = element_text(size = 12, colour = "black"), axis.title = element_text(size = 13), panel.border = element_rect(size = 1.2))

fa.p2 <-
ggplot()+
  geom_vline(xintercept = 0, linetype = 3, size = 1, color = "grey42") + geom_hline(yintercept = 0, linetype = 3, size = 1, color = "grey42")+
  geom_point(aes(x = Dim.1, y = Dim.3, shape = Feed.Type, color = Size.class),
             data = per.pca1.coord, size = 3)+
  scale_x_continuous(limits = c(-9,9)) + scale_y_continuous(limits = c(-9,9))+
  coord_equal()+
  labs(x = paste0("PC1 (",fa.pc1,"%)"), y = paste0("PC3 (",fa.pc3,"%)"), color = "Size class", shape = "Feed type")+
  theme_bw() + theme(panel.grid = element_blank(), legend.position = "right",
                     axis.text = element_text(size = 12, colour = "black"), axis.title = element_text(size = 13), panel.border = element_rect(size = 1.2))

cowplot::plot_grid(fa.p1, fa.p2, align = "hv", axis = "lrtb", nrow = 1)
```

```
ggsave("figures/Supp_figure_fatty_acid_PCAs.png", units = "in", dpi = 800, width = 10, height = 5)
```

**Fig. S4.** Ordination (PCA) of fatty acid profiles in
salmon muscle. Individuals show strong variation by feed type on PC1 and
some variation by fatty acid processing batch on PC2
(**A**) and some variation by size class on PC3
(**B**). PCAs were based on normalised and transformed
fatty acid abundances. Based on PERMANOVA results using Euclidean
distances, feed type contributed to 34.8% of the variation in fatty acid
abundances (F = 242.4, p = 0.001), size class contributed to 5.8% of the
variation (F = 20.33, p = 0.001) and fatty acid processing batch
contributed to 1.0% of the variation (F = 1.82, p = 0.086). Detailed
results from the PERMANOVAs are presented in Table S1.

```
per.dist.euc <- vegdist(per.pca.df[,which(names(per.pca.df) %in% analytes.per)], method = "euclidean")

fatty.acid.perm <- adonis2(per.dist.euc ~ per.pca.df$Feed.Type +  per.pca.df$Size.class + per.pca.df$Tapeworm + per.pca.df$Fatty_Acid_Batch, permutations = 999)
fatty.acid.perm
```

```
## Permutation test for adonis under reduced model
## Terms added sequentially (first to last)
## Permutation: free
## Number of permutations: 999
## 
## adonis2(formula = per.dist.euc ~ per.pca.df$Feed.Type + per.pca.df$Size.class + per.pca.df$Tapeworm + per.pca.df$Fatty_Acid_Batch, permutations = 999)
##                              Df SumOfSqs      R2        F Pr(>F)    
## per.pca.df$Feed.Type          1   1974.7 0.34792 242.4300  0.001 ***
## per.pca.df$Size.class         2    331.1 0.05834  20.3260  0.001 ***
## per.pca.df$Tapeworm           3     44.2 0.00780   1.8108  0.108    
## per.pca.df$Fatty_Acid_Batch   4     59.3 0.01044   1.8186  0.086 .  
## Residual                    401   3266.3 0.57550                    
## Total                       411   5675.5 1.00000                    
## ---
## Signif. codes:  0 '***' 0.001 '**' 0.01 '*' 0.05 '.' 0.1 ' ' 1
```

```
# format to print as table
fatty.acid.perm.df <- as.data.frame(fatty.acid.perm)
fatty.acid.perm.df$Variable <- row.names(fatty.acid.perm.df)
fatty.acid.perm.df$Dataset <- "fatty.acid"
row.names(fatty.acid.perm.df) <- NULL
```

PERMANOVA shows that feed type explains ~ 35% of the variation
between samples, size class ~ 5.5% and batch effects ~ 1.5%. Note that
some of the observed batch effects are driven by one group that were
sampled on the last day and had been starved by 2.5 days.

**Differential abundance testing**

Note this analysis was not included in the final version of the
manuscript.

Abundance associations with Maaslin2.
Testing for individual fatty acids associated with feed type, size class
and tapeworm detection, while controlling for sampling date as a random
effect.

```
## individual fatty acids and fatty acid sums
fa.per.ind <- merge(per.pca.df, fa.per[,c(1,grep("sum",names(fa.per)))], by = "Fish.ID", all.x = T)
fa.per.ind <- merge(fa.per.ind, fa.ratio, by = "Fish.ID", all.x = T)
row.names(fa.per.ind) <- fa.per.ind$Fish.ID
#names(fa.per.ind)
fa.per.long <- melt(fa.per.ind,
                    id.vars = names(fa.per.ind)[which(!names(fa.per.ind) %in% c(analytes.per, 
                                                                                grep("sum",names(fa.per), value = T),
                                                                                "a06.0_per","a08.0_per","a12.0_per","a20.3n3_per"))],
                    measure.vars = names(fa.per.ind)[which(names(fa.per.ind) %in% c(analytes.per, grep("sum",names(fa.per), value = T)))], 
                    value.name = "Measurement", variable.name = "Analyte")
fa.per.long$Analyte <- as.character(fa.per.long$Analyte)

# Maaslin2

if(file.exists("output_maaslin2/FA_per/all_results.tsv")){
  maas.fa.per <- read.delim("output_maaslin2/FA_per/all_results.tsv")
} else {
  maas.fa.per <- Maaslin2(
    input_data = fa.per.ind[,c(analytes.per[which(!analytes.per %in% setdiff(analytes.per, names(fa.per.ind)))],analytes.sum.per[c(1:6,8,10:12)])],
    input_metadata = fa.per.ind[,c("Feed.Type","Size.class","Tapeworm.bin","Sampling.Date")],
    fixed_effects = c("Feed.Type","Size.class","Tapeworm.bin"),
    reference = c("Size.class,Small"),
    random_effects = c("Sampling.Date"),
    output = "output_maaslin2/FA_per",
    normalization = "none", transform = "log",
  )
}
```

Associations (FDR < 0.2) between fish phenotypes and fatty acid
relative abundances in muscle.

```
maas.fa.per <- maas.fa.per[order(maas.fa.per$metadata, maas.fa.per$value, maas.fa.per$coef),]

maas.fa.per[which(maas.fa.per$qval < 0.2 & maas.fa.per$value != ".Q"),c(1:5,9)] %>%
  kbl(row.names = FALSE) %>%
  kable_styling(bootstrap_options = c("striped","hover")) %>%
  column_spec(4, color = "black", 
              background = spec_color(maas.fa.per[which(maas.fa.per$qval < 0.2 & maas.fa.per$value != ".Q"),"coef"], 
                                      option = "viridis", scale_from = c(-1.6,1.5), alpha = 0.5))
```

| feature | metadata | value | coef | stderr | qval |
| --- | --- | --- | --- | --- | --- |
| a22.5n6\_per | Feed.Type | Feed2 | -1.5368029 | 0.2964605 | 0.0374928 |
| a18.3n3\_per | Feed.Type | Feed2 | -0.5646422 | 0.0187982 | 0.0014842 |
| a20.4n6\_ARA\_per | Feed.Type | Feed2 | -0.4692570 | 0.0262385 | 0.0030640 |
| a22.1n9\_per | Feed.Type | Feed2 | -0.4600432 | 0.0829897 | 0.0374928 |
| a20.2n6\_per | Feed.Type | Feed2 | -0.3596159 | 0.0472536 | 0.0138748 |
| a20.1n9\_per | Feed.Type | Feed2 | -0.2545026 | 0.0236752 | 0.0069060 |
| sum\_20.1\_per | Feed.Type | Feed2 | -0.2473796 | 0.0521657 | 0.0466442 |
| sum\_n3\_per | Feed.Type | Feed2 | -0.2048220 | 0.0177687 | 0.0064261 |
| sum\_polyunsat\_per | Feed.Type | Feed2 | -0.1884549 | 0.0099872 | 0.0021802 |
| sum\_n6\_per | Feed.Type | Feed2 | -0.1743076 | 0.0114585 | 0.0031873 |
| a22.6n3\_DHA\_per | Feed.Type | Feed2 | -0.1533495 | 0.0344468 | 0.0583674 |
| a18.2n6\_per | Feed.Type | Feed2 | -0.1276982 | 0.0063516 | 0.0044491 |
| a18.0\_per | Feed.Type | Feed2 | -0.0711541 | 0.0119295 | 0.0374928 |
| sum\_saturated\_per | Feed.Type | Feed2 | 0.0658629 | 0.0076772 | 0.0362939 |
| a18.1n9\_per | Feed.Type | Feed2 | 0.1070806 | 0.0069716 | 0.0042796 |
| sum\_monounsat\_per | Feed.Type | Feed2 | 0.1088109 | 0.0080783 | 0.0071082 |
| a20.5n3\_EPA\_per | Feed.Type | Feed2 | 0.1202846 | 0.0311234 | 0.0754963 |
| sum\_18.1\_per | Feed.Type | Feed2 | 0.1226383 | 0.0065407 | 0.0100494 |
| sum\_22.1\_per | Feed.Type | Feed2 | 0.1668159 | 0.0122985 | 0.0844600 |
| a20.4n3\_per | Feed.Type | Feed2 | 0.1745421 | 0.0324120 | 0.0374928 |
| a18.1n7\_per | Feed.Type | Feed2 | 0.1939246 | 0.0255376 | 0.0236835 |
| a22.5n3\_DPA\_per | Feed.Type | Feed2 | 0.2576407 | 0.0189238 | 0.0028825 |
| a22.1n11\_per | Feed.Type | Feed2 | 0.3734078 | 0.0611454 | 0.0350749 |
| sum\_16.1\_per | Feed.Type | Feed2 | 0.3929733 | 0.0275146 | 0.0000000 |
| a14.0\_per | Feed.Type | Feed2 | 0.4330163 | 0.0102679 | 0.0001030 |
| a17.0\_per | Feed.Type | Feed2 | 0.4518188 | 0.0936806 | 0.0458396 |
| a16.1n7\_per | Feed.Type | Feed2 | 0.4519054 | 0.0133546 | 0.0000000 |
| a15.0\_per | Feed.Type | Feed2 | 0.9397879 | 0.0305669 | 0.0011673 |
| a18.1n11\_per | Size.class | .L | -0.1983012 | 0.0767583 | 0.0318392 |
| a16.1n9\_per | Size.class | .L | -0.1102401 | 0.0304513 | 0.0017925 |
| a22.6n3\_DHA\_per | Size.class | .L | -0.0695579 | 0.0076504 | 0.0000000 |
| a18.1n9\_per | Size.class | .L | -0.0495835 | 0.0059903 | 0.0000000 |
| sum\_18.1\_per | Size.class | .L | -0.0421488 | 0.0029855 | 0.0000000 |
| a22.1n11\_per | Size.class | .L | -0.0378807 | 0.0126581 | 0.0100494 |
| sum\_monounsat\_per | Size.class | .L | -0.0323895 | 0.0023950 | 0.0000000 |
| a20.4n3\_per | Size.class | .L | -0.0322577 | 0.0089195 | 0.0017925 |
| sum\_22.1\_per | Size.class | .L | -0.0283844 | 0.0092523 | 0.0083350 |
| a20.2n6\_per | Size.class | .L | -0.0234661 | 0.0121909 | 0.1193777 |
| a18.2n6\_per | Size.class | .L | -0.0211964 | 0.0025052 | 0.0000000 |
| sum\_EPA\_DHA\_per | Size.class | .L | -0.0199225 | 0.0060334 | 0.0044491 |
| sum\_n6\_per | Size.class | .L | -0.0142167 | 0.0024393 | 0.0000001 |
| sum\_polyunsat\_per | Size.class | .L | 0.0141266 | 0.0029373 | 0.0000179 |
| sum\_n3\_per | Size.class | .L | 0.0425998 | 0.0047915 | 0.0000000 |
| a16.1n7\_per | Size.class | .L | 0.0430291 | 0.0116826 | 0.0014842 |
| a24.1n9\_per | Size.class | .L | 0.0437439 | 0.0255809 | 0.1780832 |
| sum\_16.1\_per | Size.class | .L | 0.0466809 | 0.0240698 | 0.1169169 |
| a14.0\_per | Size.class | .L | 0.0527367 | 0.0062757 | 0.0000000 |
| a18.0\_per | Size.class | .L | 0.0589926 | 0.0062890 | 0.0000000 |
| a20.5n3\_EPA\_per | Size.class | .L | 0.0616722 | 0.0079132 | 0.0000000 |
| a22.5n6\_per | Size.class | .L | 0.0637421 | 0.0369154 | 0.1739173 |
| a20.4n6\_ARA\_per | Size.class | .L | 0.0662364 | 0.0109068 | 0.0000000 |
| sum\_saturated\_per | Size.class | .L | 0.0716423 | 0.0041983 | 0.0000000 |
| a16.0\_per | Size.class | .L | 0.0751475 | 0.0042420 | 0.0000000 |
| a20.0\_per | Size.class | .L | 0.0981928 | 0.0259963 | 0.0011673 |
| a18.3n3\_per | Size.class | .L | 0.1207795 | 0.0095853 | 0.0000000 |
| a24.5n3\_per | Size.class | .L | 0.3001716 | 0.0578774 | 0.0000030 |
| a22.0\_per | Size.class | .L | 0.3780860 | 0.0565396 | 0.0000000 |
| a17.0\_per | Tapeworm.bin | TRUE | -0.1005517 | 0.0607657 | 0.1974998 |
| a22.6n3\_DHA\_per | Tapeworm.bin | TRUE | -0.0473953 | 0.0112184 | 0.0002169 |
| a18.4n3\_per | Tapeworm.bin | TRUE | -0.0455933 | 0.0250375 | 0.1488383 |
| sum\_EPA\_DHA\_per | Tapeworm.bin | TRUE | -0.0311143 | 0.0088411 | 0.0024232 |
| a18.2n6\_per | Tapeworm.bin | TRUE | -0.0136150 | 0.0036893 | 0.0014842 |
| a16.0\_per | Tapeworm.bin | TRUE | -0.0109478 | 0.0062428 | 0.1661512 |
| a20.1n9\_per | Tapeworm.bin | TRUE | 0.0164817 | 0.0083314 | 0.1082204 |
| sum\_20.1\_per | Tapeworm.bin | TRUE | 0.0222467 | 0.0111274 | 0.1071013 |
| a22.5n3\_DPA\_per | Tapeworm.bin | TRUE | 0.0374020 | 0.0113785 | 0.0045043 |
| a20.4n6\_ARA\_per | Tapeworm.bin | TRUE | 0.0681899 | 0.0160718 | 0.0002095 |
| a20.4n3\_per | Tapeworm.bin | TRUE | 0.0831721 | 0.0130942 | 0.0000000 |
| a20.2n6\_per | Tapeworm.bin | TRUE | 0.1072019 | 0.0178899 | 0.0000000 |
| a22.0\_per | Tapeworm.bin | TRUE | 0.1869144 | 0.0828931 | 0.0611678 |

```
# write out for supp tables

if(!file.exists("tables/Supp_table_fattyacid_maaslin_res_230314.csv")){
  paper.supp.tab.maas.fa <- maas.fa.per[which(maas.fa.per$qval < 0.2 & maas.fa.per$value != ".Q"),]
  paper.supp.tab.maas.fa$Fatty.acid <- sapply(paper.supp.tab.maas.fa$feature, function(x){
    if(!grepl("^sum_",x)){
      x <- gsub("^a","",x)
      x <- gsub("\\.",":",x)
      x <- gsub("n","(n-",x)
      x <- gsub("_per",")",x)
    }
    return(x)
  })
  paper.supp.tab.maas.fa$metadata <- gsub("Tapeworm.bin","Cestode.present",paper.supp.tab.maas.fa$metadata)
  write.csv(paper.supp.tab.maas.fa[,c("Fatty.acid","metadata","value","coef","stderr","N","N.not.0","pval","qval")],
            "tables/Supp_table_fattyacid_maaslin_res_230314.csv",
            row.names = F, quote = F)
}
```

## 16S metabarcoding

Data processed using BAMSE, including filtering of Eukaryotes,
chimera filtering and low copy number filtering. Also removing ASVs
flagged as contaminants by decontam frequency (using raw sequence count)
and prevalence (using extraction blanks) and ASVs assigned to
Chloroplast or Mitochondria.

```
# load & process data

asv.cts <- read.csv("data/Microbiota_16S/HoloFish_16S_ASV_counts.csv")
row.names(asv.cts) <- asv.cts$ASV.ID

asv.taxa <- read.csv("data/Microbiota_16S/ASV_taxa.txt")
names(asv.taxa)[1] <- "ASV.ID"
row.names(asv.taxa) <- asv.taxa$ASV.ID

m16S.info <- read.csv("data/Microbiota_16S/HoloFish_16S_sample_type_info_and_processing_stats.csv")

asv.tab <- t(asv.cts[,-1])

m16S.info <- merge(m16S.info, meta[,c("Fish.ID",metavar.fish[c(1:3,5:6)],metavar.exp)], by = "Fish.ID",
                   all.x = T, all.y = F)
row.names(m16S.info) <- m16S.info$Sample

m16S.ps <- phyloseq(otu_table(asv.tab, taxa_are_rows=FALSE),
                    tax_table(as.matrix(asv.taxa[,-1])),
                    sample_data(m16S.info)
)
```

**QC**: contaminant identification

```
m16S.contam.freq <- isContaminant(m16S.ps, method="frequency", conc="preprocessed")
table(m16S.contam.freq$contaminant)
```

```
## 
## FALSE  TRUE 
##   376     8
```

```
asv.taxa[row.names(m16S.contam.freq[which(m16S.contam.freq$contaminant),]), c("Phylum","Genus")]
```

```
##                 Phylum            Genus
## ASV_72  Proteobacteria   Photobacterium
## ASV_86      Firmicutes       Mycoplasma
## ASV_123  Cyanobacteria             <NA>
## ASV_139     Firmicutes   Staphylococcus
## ASV_143 Proteobacteria             <NA>
## ASV_196 Proteobacteria Phenylobacterium
## ASV_245     Firmicutes       Mycoplasma
## ASV_402     Firmicutes       Mycoplasma
```

```
m16S.contam.freq.ids <- row.names(m16S.contam.freq[which(m16S.contam.freq$contaminant),])

sample_data(m16S.ps)$Lab.neg <- sample_data(m16S.ps)$Sample.Type == "blank"
m16S.contam.prev <- isContaminant(m16S.ps, method="prevalence", neg="Lab.neg")
table(m16S.contam.prev$contaminant)
```

```
## 
## FALSE 
##   384
```

```
# none

## filter out Mitochondria and Chloroplast sequences
excluded.seqs <- unique(c(
  asv.taxa[which(asv.taxa$ASV.ID %in% colnames(asv.tab) & 
                   asv.taxa$Family == "Mitochondria"),"ASV.ID"],
  asv.taxa[which(asv.taxa$ASV.ID %in% colnames(asv.tab) & 
                   asv.taxa$Order == "Chloroplast"),"ASV.ID"]
))

asv.tab.filt <- asv.tab[,which(!colnames(asv.tab) %in% c(excluded.seqs,m16S.contam.freq.ids))]
asv.tab.filt <- asv.tab.filt[which(rowSums(asv.tab.filt) > 0),]
# dim(asv.tab.filt) #141 x 311
# which samples have been excluded due to too many contaminants?
# setdiff(rownames(asv.tab),rownames(asv.tab.filt))

## Phyloseq of filtered data ##
m16S.ps.filt <- phyloseq(otu_table(asv.tab.filt, taxa_are_rows=FALSE),
                    tax_table(as.matrix(asv.taxa[colnames(asv.tab.filt),-1])),
                    sample_data(m16S.info[which(m16S.info$Sample %in% rownames(asv.tab.filt)),])
)

## normalisation and transformations
m16S.ps.relab <- transform_sample_counts(m16S.ps.filt, function(OTU) OTU/sum(OTU))

m16S.ps.clr <- transform_sample_counts(m16S.ps.filt, function(OTU) OTU+1) # pseudo-count
m16S.ps.clr <- microbiome::transform(m16S.ps.clr, 'clr')
```

**QC**: Blanks

Check remaining taxa in blanks.

```
m16S.ps.blanks <- subset_samples(m16S.ps.filt, Sample.Type == "blank")
m16S.ps.blanks <- prune_taxa(taxa_sums(m16S.ps.blanks)>0, m16S.ps.blanks)
m16S.ps.blanks.relab <- transform_sample_counts(m16S.ps.blanks, function(OTU) OTU/sum(OTU))

plot_bar(m16S.ps.blanks.relab, x = "Sample", fill = "Family")+
  theme_classic()+
  theme(legend.position = "right")
```

```
## Warning in psmelt(physeq): The sample variables: 
## Sample
##  have been renamed to: 
## sample_Sample
## to avoid conflicts with special phyloseq plot attribute names.
```

**Taxa remaining in blank samples after decontam**. All
are low-abundant taxa, some likely water contaminants
(*Rhizobiaceae*, *Xanthomonadaceae*,
*Sphingobacteriaceae*), some likely sample cross-contamination
(*Mycoplasmataceae*).

### Supp figure: heatmap

Excluding blanks and only showing taxa detected in > 5
samples.

```
m16S.df.clr <- as.data.frame(otu_table(m16S.ps.clr))
# exclude extraction blanks
m16S.df.clr <- m16S.df.clr[grep("Ext_blank",row.names(m16S.df.clr), invert = T),]
#change clr mins to NA values for heatmap
m16S.clr.pseudozeros <- sapply(row.names(m16S.df.clr), function(x){min(m16S.df.clr[x,])})
m16S.df.clr.nas <- as.matrix(t(m16S.df.clr))
for (s in colnames(m16S.df.clr.nas)) {
  m16S.df.clr.nas[which(m16S.df.clr.nas[,s] == m16S.clr.pseudozeros[s]),s] <- as.double("NA")
}

# filter out low-abundant ASVs
m16S.df.clr.nas <- m16S.df.clr.nas[which(rowSums(!is.na(m16S.df.clr.nas)) > 5),]

# which genera are represented?
sort(table(asv.taxa[which(asv.taxa$ASV.ID %in% rownames(m16S.df.clr.nas)),"Genus"]), decreasing = T)
```

```
## 
##          Mycoplasma Mycoplasmataceae_sp      Photobacterium       Lactobacillus 
##                  17                  13                   9                   8 
##             Neiella          Aliivibrio           Brevinema 
##                   6                   3                   1
```

```
asv.list.hm <- asv.taxa[order(asv.taxa$Order, asv.taxa$Family, asv.taxa$Genus),"ASV.ID"]
asv.list.hm <- asv.list.hm[which(asv.list.hm %in% rownames(m16S.df.clr.nas))]

m16S.sample.list.hm <- m16S.info[order(m16S.info$Sample.Type, m16S.info$Feed.Type),"Sample"]
m16S.sample.list.hm <- m16S.sample.list.hm[which(m16S.sample.list.hm %in% colnames(m16S.df.clr.nas))]

asv.anno.hm <- data.frame(
  Clade=factor(sapply(rownames(m16S.df.clr.nas), function(x){
    if(is.na(asv.taxa[which(asv.taxa$ASV.ID == x),"Order"])){
      "Other"
    } else if(asv.taxa[which(asv.taxa$ASV.ID == x),"Order"] == "Alteromonadales"){
      "Alteromonadales"
    } else if(is.na(asv.taxa[which(asv.taxa$ASV.ID == x),"Family"])){
      "Other"
    } else if(asv.taxa[which(asv.taxa$ASV.ID == x),"Family"] == "Mycoplasmataceae"){
      "Mycoplasmataceae"
    } else if(is.na(asv.taxa[which(asv.taxa$ASV.ID == x),"Genus"])){
      "Other"
    } else {
      asv.taxa[which(asv.taxa$ASV.ID == x),"Genus"]
    }
  }), levels = c("Alteromonadales","Brevinema","Lactobacillus","Mycoplasmataceae","Aliivibrio","Photobacterium","Other")),
  row.names = rownames(m16S.df.clr.nas)
)

m16S.samples.anno.hm <- data.frame(
  Sample.Type=sapply(colnames(m16S.df.clr.nas), function(x){
    m16S.info[which(m16S.info$Sample == x),"Sample.Type"]
  }),
  Feed.Type=sapply(colnames(m16S.df.clr.nas), function(x){
    m16S.info[which(m16S.info$Sample == x),"Feed.Type"]
    })
)

# table(asv.anno.hm$Clade)
# table(m16S.samples.anno.hm$Sample.Type)
# table(m16S.samples.anno.hm$Feed.Type)

m16S.hm.colors <- list(
  Clade=c(brewer.pal(6, "Set1"),"grey50"),
  Sample.Type=c(FeedType=brewer.pal(6,"Greens")[2], GutContent=brewer.pal(6,"Greens")[4], GutTissue=brewer.pal(6,"Greens")[6]),
  Feed.Type=c(Feed1="#ffa200", Feed2="#b700ff")
  
)
names(m16S.hm.colors$Clade) <- levels(asv.anno.hm$Clade)

# add to pheatmap to save: filename = "figures/Supp_figure_16S_heatmap.png", width = 7, height = 7
pheatmap(m16S.df.clr.nas[asv.list.hm,m16S.sample.list.hm], cluster_cols = F, cluster_rows = F,
         annotation_row = asv.anno.hm, annotation_col = m16S.samples.anno.hm, annotation_colors = m16S.hm.colors,
         show_rownames = F, show_colnames = F)
```

**Fig. S7**. Microbiota community composition of salmon
gut content compared to salmon gut 1529 mucosa scrapes and feed pellet,
determined by 16S metabarcoding. (**A**) Heatmap of the
abundance (centre-log-ratio normalised) of the 64 amplicon sequence
variants (ASVs) present in at least 5 samples after quality control.
ASVs are annotated by their taxonomic clade, where “other” indicates
ASVs that could not be assigned taxonomy at the level of order. ASVs
within the order *Alteromonadales* were classified as the genus
*Neiella*. ASVs within the family *Mycoplasmataceae* were
classified as the genus *Mycoplasma* (17 ASVs) or an unknown
genus within the *Mycoplasmataceae* family (13 ASVs). Other
taxonomic clades are provided at the genus level. Samples are annotated
by sample type and commercial feed type.

### Supp figure: Venn

```
# Venn - include ASVs detected in at least 1 sample of sample.type

m16S.pellet.samples <- m16S.info[which(m16S.info$Sample.Type == "FeedType"),"Sample"]
m16S.gut.samples <- m16S.info[which(m16S.info$Sample.Type == "GutContent"),"Sample"]
m16S.mucosa.samples <- m16S.info[which(m16S.info$Sample.Type == "GutTissue"),"Sample"]

m16S.venn.df <- list(
  Pellet=rownames(m16S.df.clr.nas[which(rowSums(is.na(m16S.df.clr.nas[,which(colnames(m16S.df.clr.nas) %in% m16S.pellet.samples)])) < length(which(colnames(m16S.df.clr.nas) %in% m16S.pellet.samples))),]),
  GutContent=rownames(m16S.df.clr.nas[which(rowSums(is.na(m16S.df.clr.nas[,which(colnames(m16S.df.clr.nas) %in% m16S.gut.samples)])) < length(which(colnames(m16S.df.clr.nas) %in% m16S.gut.samples))),]),
  GutMucosa=rownames(m16S.df.clr.nas[which(rowSums(is.na(m16S.df.clr.nas[,which(colnames(m16S.df.clr.nas) %in% m16S.mucosa.samples)])) < length(which(colnames(m16S.df.clr.nas) %in% m16S.mucosa.samples))),])
)

ggVennDiagram(m16S.venn.df,
              category.names = c("Pellet","Gut\ncontent","Gut\nmucosa"),
              label = "count", label_alpha = 0)+
  scale_fill_gradient(low = NA, high = NA)+
  scale_color_manual(values = brewer.pal(6,"Greens")[c(2,4,6)])+
  theme(legend.position = "none")
```

```
ggsave("figures/Supp_figure_16S_venn.png", units = "in", dpi = 800, width = 5, height = 5)
```

**Fig. S7**. Microbiota community composition of salmon
gut content compared to salmon gut 1529 mucosa scrapes and feed pellet,
determined by 16S metabarcoding. (**B**) Venn diagram of
the 64 ASVs in **Fig. S7A** detected at least once within
each sample type. The 16 ASVs unique to gut content samples included 14
low-abundant *Mycoplasmataceae* taxa and 2
*Alteromonadales* taxa.

```
## what are unique to feed pellet and gut content?
# asv.taxa[which(asv.taxa$ASV.ID %in% setdiff(m16S.venn.df$Pellet,unique(c(m16S.venn.df$GutContent,m16S.venn.df$GutMucosa)))),]
## 5 x Lactobacillus
# asv.taxa[which(asv.taxa$ASV.ID %in% setdiff(m16S.venn.df$GutContent,unique(c(m16S.venn.df$Pellet,m16S.venn.df$GutMucosa)))),]
## 14 x low-abundant mycoplasma + 2 x Alteromonadales/Neiella
```

```
# set up gut content samples for comparison with MAGs
m16S.ps.gut <- subset_samples(m16S.ps.filt, Sample.Type == "GutContent")
m16S.ps.gut <- prune_taxa(taxa_sums(m16S.ps.gut)>0, m16S.ps.gut)

m16S.df.gut <- as.data.frame(otu_table(m16S.ps.gut))
m16S.gut.taxa <- as.data.frame(tax_table(m16S.ps.gut))
m16S.gut.taxa$ASV.ID <- row.names(m16S.gut.taxa)
```

## MetaG MAG evaluation

Processing steps:

- Adapter removal, duplicate removal, mapping to reference genome
- Merging of BGI and Novogene unmapped fastq files
- Coassembly (`megahit`) by feed type
- Automatic binning on each assembly
  (`metabin2, maxbin2, concoct`)
- Automatic refinement of bins (`metaWRAP`)
- Manual refinement of bins (`Anvi'o`)
- Single assembly (`metaspades`) of a subset of selected
  samples to improve some low-quality (< 70% complete) bins
  - Manual binning (`Anvi'o`)
- Dereplication of coassembled (> 50% complete) and
  single-assembled (> 70% complete) bins at the species level
  (including salmonid + tapeworm bins from other studies)
- Taxonomy assignment with `gtdbtk`
- Abundance calculated by mapping only Novogene unmapped reads back to
  bins, then taking Anvi’o abundance values (normalised mean coverage) per
  sample per bin.

```
# metag specific data
# mapping of Novogene Illumina reads to bins
metag.stats <- read.csv("data/MetaG/log_final_bins_sample_mapped_stats.csv")

meta$metag.sequencing.depth <- sapply(meta$Fish.ID, function(x){
  if(x %in% metag.stats$Fish.ID){
    metag.stats[which(metag.stats$Fish.ID == x), "total"]
  } else {
    NA
  }
})
metavar.exp <- c(metavar.exp, "metag.sequencing.depth")

metag.stats$Neg.control <- grepl("ExtNeg",metag.stats$Fish.ID)

# bin values: using estimates from Anvio
bin.abund <- read.csv("data/MetaG/final_MAGv6_bin_abundance.csv")
bin.det <- read.csv("data/MetaG/final_MAGv6_bin_detection.csv")
# samples = columns, bins = rows

row.names(bin.abund) <- bin.abund$MAG.ID
row.names(bin.det) <- bin.det$MAG.ID

# bin info
bin.taxonomy <- read.csv("data/MetaG/final_bins_taxonomy_formatted.csv")
bin.stats <- read.csv("data/MetaG/final_bins_checkm_quality_info.csv")

bin.abund.long <- merge(
  melt(bin.abund, id.vars = "MAG.ID", variable.name = "Fish.ID", value.name = "Abundance"),
  melt(bin.det, id.vars = "MAG.ID", variable.name = "Fish.ID", value.name = "Detection"),
  by = c("MAG.ID","Fish.ID")
)

# Filter abundance based on detection: only class a bin as present in a sample if > 30% of nucleotides in bin are at least 1X
cutoff.bin.det <- 0.3
bin.abund.long$Filtered.abundance <- sapply(seq(1,nrow(bin.abund.long),1), function(i){
  if(bin.abund.long[i,"Detection"] > cutoff.bin.det){
    bin.abund.long[i,"Abundance"]
  } else {
    # set to zero
    0
  }
})

# Presence/absence
bin.abund.long$Filtered.detected <- bin.abund.long$Filtered.abundance > 0

bin.abund.long <- merge(bin.abund.long, meta[,c("Fish.ID",metavar.fish,metavar.exp)], all.x = T, all.y = F)
bin.abund.long$Neg.control <- grepl("ExtNeg",bin.abund.long$Fish.ID)
bin.abund.long$metag.sequencing.depth <- sapply(bin.abund.long$Fish.ID, function(x){
  if(x %in% metag.stats$Fish.ID){
    metag.stats[which(metag.stats$Fish.ID == x), "total"]
  } else {
    NA
  }
})
```

**MAG catalogue details**

For bin abundance per sample calculations, only bins with > 30% of
their genome covered by at least 1 read are classed as “detected” (all
others have abundance values set to 0).

```
# Samples bin detected in
bin.stats$Samples.detected <- sapply(bin.stats$MAG.ID, function(x){
  nrow(bin.abund.long[which(bin.abund.long$MAG.ID == x & !bin.abund.long$Neg.control & bin.abund.long$Filtered.abundance > 0),])
})

# Negative controls bin detected in
bin.stats$Neg.controls.detected <- sapply(bin.stats$MAG.ID, function(x){
  nrow(bin.abund.long[which(bin.abund.long$MAG.ID == x & bin.abund.long$Neg.control & bin.abund.long$Filtered.abundance > 0),])
})

# Mean abundance of bin in samples (in which it was detected in)
bin.stats$Samples.mean.abundance <- sapply(bin.stats$MAG.ID, function(x){
  round(mean(bin.abund.long[which(bin.abund.long$MAG.ID == x & !bin.abund.long$Neg.control & bin.abund.long$Filtered.abundance > 0),"Filtered.abundance"]), digits = 3)
})

# check QS bins: should be > 50
bin.stats$qs50 <- bin.stats$completeness - (5 * bin.stats$contamination)
```

### MAG catalogue

```
bin.stats <- bin.stats[order(bin.stats$MAG.ID),c("MAG.ID","qs50","completeness","contamination","Samples.detected","Neg.controls.detected","Samples.mean.abundance")]
bin.stats %>%
  kbl(row.names = FALSE) %>%
  kable_styling(bootstrap_options = c("striped","hover")) %>%
  column_spec(2, background = spec_color(bin.stats$qs50, option = "magma", begin = 0.5, alpha = 0.5, scale_from = c(50,100)))
```

| MAG.ID | qs50 | completeness | contamination | Samples.detected | Neg.controls.detected | Samples.mean.abundance |
| --- | --- | --- | --- | --- | --- | --- |
| MAG01\_Mycoplasma\_salmoninae | 77.44 | 86.24 | 1.76 | 393 | 2 | 51.181 |
| MAG02\_Mycoplasma\_sp | 92.30 | 96.15 | 0.77 | 215 | 0 | 0.792 |
| MAG03\_Mycoplasma\_sp | 92.34 | 98.59 | 1.25 | 86 | 0 | 0.438 |
| MAG04\_Mycoplasma\_sp | 89.61 | 97.31 | 1.54 | 73 | 0 | 0.302 |
| MAG05\_Mycoplasma\_sp | 89.34 | 90.59 | 0.25 | 50 | 0 | 0.702 |
| MAG06\_Photobacterium\_phosphoreum | 96.05 | 97.40 | 0.27 | 183 | 0 | 1.711 |
| MAG07\_Photobacterium\_iliopiscarium | 94.95 | 97.35 | 0.48 | 20 | 0 | 1.450 |
| MAG08\_Aliivibrio\_sp | 61.51 | 98.81 | 7.46 | 72 | 0 | 1.743 |
| MAG09\_Aliivibrio\_sp | 89.61 | 95.26 | 1.13 | 27 | 0 | 2.041 |
| MAG10\_Aliivibrio\_salmonicida | 96.75 | 97.40 | 0.13 | 7 | 0 | 1.109 |
| MAG11\_Brevinema\_sp | 93.26 | 93.26 | 0.00 | 105 | 0 | 0.494 |
| MAG12\_Carnobacterium\_maltaromaticum | 77.96 | 98.91 | 4.19 | 57 | 0 | 0.143 |
| MAG13\_Lactobacillus\_johnsonii | 80.34 | 93.89 | 2.71 | 32 | 0 | 0.065 |
| MAG14\_Clostridium\_ljungdahlii | 75.91 | 84.21 | 1.66 | 2 | 0 | 0.347 |
| MAG15\_Psychromonas\_sp | 94.60 | 100.00 | 1.08 | 2 | 0 | 1.464 |
| MAG16\_Prevotella\_sp | 79.48 | 94.18 | 2.94 | 0 | 0 | NaN |

### Extraction negatives

```
read.depth.cutoff <- 1e5
exclude.sids <- metag.stats[which(metag.stats$primary < read.depth.cutoff & !metag.stats$Neg.control),"Fish.ID"]

ggplot(metag.stats, aes(total, primary, color = Neg.control, shape = Neg.control))+
  geom_abline(slope = 1, intercept = 0, color = "grey50", linetype = 3)+
  geom_hline(yintercept = read.depth.cutoff, color = "blue", linetype = 2)+
  geom_point(size = 2)+
  scale_x_continuous(trans = "log10", limits = c(1e3,5e8))+
  scale_y_continuous(trans = "log10", limits = c(1e3,5e8))+
  labs(x = "No. of reads post-processing (Novo)", y = "No. of reads aligned to bins")+
  theme_bw()+ 
  theme(axis.text = element_text(size = 13, colour = "black"), axis.title = element_text(size = 12))
```

**Reads mapping to bins vs sampe sequencing depth for samples
and extraction negatives**. Majority of samples have at least
1,000,000 reads remaining post-processing and at least 100,000 reads
(/alignments) to the bins. Negative controls tend to have lower read
numbers as well. However, since it’s likely there’s been some
cross-contamination from high abundant samples into low biomass samples,
have excluded samples with similar levels of read as aligned to bins as
most negative controls (i.e. < 100,000). Number of excluded samples:
6.

MAGs detected in extraction blanks afer filtering:

```
bin.abund.long[which(bin.abund.long$Neg.control & bin.abund.long$Filtered.abundance > 0),c("MAG.ID","Fish.ID","Detection","Filtered.abundance")]
```

```
##                           MAG.ID Fish.ID Detection Filtered.abundance
## 6385 MAG01_Mycoplasma_salmoninae ExtNeg2 0.8944247           47.41043
## 6401 MAG01_Mycoplasma_salmoninae ExtNeg3 0.6142383           54.28809
```

Only 2 of the 7 negative controls have bins detected after filtering
- both only registering the most abundant bin, *Mycoplasma
salmoninae*. This is likely cross-contamination from neighbouring
high-abundance samples.

```
# exclude neg controls from rest of data analysis
# exclude low depth samples from rest of data analysis
# Note Prevotella is also zero in all samples after filtering, can remove too
bin.samples.abund <- subset(bin.abund.long, !Neg.control & MAG.ID != "MAG16_Prevotella_sp" & !Fish.ID %in% exclude.sids)
```

### MAG vs 16S data

Compare MAG relative abundance and 16S ASV relative abundance, at the
genus or higher level, using the taxa most frequently detected in both
datasets.

```
m16S.gut.taxa$sum.gut <- sapply(m16S.gut.taxa$ASV.ID, function(x){
  sum(m16S.df.gut[,x]) / sum(m16S.df.gut)
})

comparison <- data.frame(
  Genus=c("Mycoplasma_related","Photobacterium","Aliivibrio","Psychromonas","Carnobacterium",
          "Brevinema","Clostridium","Vibrionaceae","Alteromonadales","Lactobacillus","Neiella","z.Other"),
  bin.abund=c(
    sum(bin.samples.abund[grep("mycoplasm",bin.samples.abund$MAG.ID, ignore.case = T),"Filtered.abundance"]) / sum(bin.samples.abund[,"Filtered.abundance"]),
    sum(bin.samples.abund[grep("Photobacterium_",bin.samples.abund$MAG.ID),"Filtered.abundance"]) / sum(bin.samples.abund[,"Filtered.abundance"]),
    sum(bin.samples.abund[grep("Aliivibrio_",bin.samples.abund$MAG.ID),"Filtered.abundance"]) / sum(bin.samples.abund[,"Filtered.abundance"]),
    sum(bin.samples.abund[grep("Psychromonas_",bin.samples.abund$MAG.ID),"Filtered.abundance"]) / sum(bin.samples.abund[,"Filtered.abundance"]),
    sum(bin.samples.abund[grep("Carnobacterium_",bin.samples.abund$MAG.ID),"Filtered.abundance"]) / sum(bin.samples.abund[,"Filtered.abundance"]),
    sum(bin.samples.abund[grep("Brevinema_",bin.samples.abund$MAG.ID),"Filtered.abundance"]) / sum(bin.samples.abund[,"Filtered.abundance"]),
    sum(bin.samples.abund[grep("Clostridium_",bin.samples.abund$MAG.ID),"Filtered.abundance"]) / sum(bin.samples.abund[,"Filtered.abundance"]),
    sum(bin.samples.abund[c(grep("Aliivibrio_",bin.samples.abund$MAG.ID),grep("Photobacterium_",bin.samples.abund$MAG.ID)),"Filtered.abundance"]) / sum(bin.samples.abund[,"Filtered.abundance"]),
    sum(bin.samples.abund[grep("Psychromonas_",bin.samples.abund$MAG.ID),"Filtered.abundance"]) / sum(bin.samples.abund[,"Filtered.abundance"]),
    sum(bin.samples.abund[grep("Lactobacillus_",bin.samples.abund$MAG.ID),"Filtered.abundance"]) / sum(bin.samples.abund[,"Filtered.abundance"]),
    sum(bin.samples.abund[grep("Neiella-",bin.samples.abund$MAG.ID),"Filtered.abundance"]) / sum(bin.samples.abund[,"Filtered.abundance"]),
    0
  ),
  ASV.abund=c(
    sum(m16S.gut.taxa[which(m16S.gut.taxa$Family == "Mycoplasmataceae"),"sum.gut"]),
    sum(m16S.gut.taxa[which(m16S.gut.taxa$Genus == "Photobacterium"),"sum.gut"]),
    sum(m16S.gut.taxa[which(m16S.gut.taxa$Genus == "Aliivibrio"),"sum.gut"]),
    sum(m16S.gut.taxa[which(m16S.gut.taxa$Genus == "Psychromonas"),"sum.gut"]),
    sum(m16S.gut.taxa[which(m16S.gut.taxa$Genus == "Carnobacterium"),"sum.gut"]),
    sum(m16S.gut.taxa[which(m16S.gut.taxa$Genus == "Brevinema"),"sum.gut"]),
    sum(m16S.gut.taxa[which(m16S.gut.taxa$Genus == "Clostridium"),"sum.gut"]),
    sum(m16S.gut.taxa[which(m16S.gut.taxa$Family == "Vibrionaceae"),"sum.gut"]),
    sum(m16S.gut.taxa[which(m16S.gut.taxa$Order == "Alteromonadales"),"sum.gut"]),
    sum(m16S.gut.taxa[which(m16S.gut.taxa$Genus == "Lactobacillus"),"sum.gut"]),
    sum(m16S.gut.taxa[which(m16S.gut.taxa$Genus == "Neiella"),"sum.gut"]),
    sum(m16S.gut.taxa[which(!m16S.gut.taxa$Genus %in% c("Psychromonas","Carnobacterium","Brevinema","Clostridium","Prevotella") &
                     !m16S.gut.taxa$Family %in% c("Mycoplasmataceae","Vibrionaceae") &
                       !m16S.gut.taxa$Order %in% c("Alteromonadales")),"sum.gut"])
  )
)

m16s.top.gen <- unique(m16S.gut.taxa[1:50,"Genus"])
m16s.top.gen <- m16s.top.gen[!is.na(m16s.top.gen)]
# print(paste0(m16s.top.gen, collapse = ", "))
```

The top 50 most abundant ASVs fall into 8 genera, namely: Mycoplasma,
Photobacterium, Brevinema, Aliivibrio, Lactobacillus,
Mycoplasmataceae\_sp, Carnobacterium, Neiella. The majority of these
represent the major genera of the MAGs assembled from the metagenomic
data. Comparing abundances, there is generally good agreement between
most abundance bin taxonomies and most abundant 16S ASVs. Note there are
some disagreements in bacterial taxonomy between databases (GTDB used
for MAGs, SILVA used for 16S), which may also contribute to
inconsistencies.

MAGs missing from 16S data:

- Psychromonas (order Alteromonadales)
- Clostridium
- can’t easily compare “other” due to the way MAG vs 16S abundance is
  calculated

16S ASVs missing from MAGs:

- Neilla (order Alteromonadales) - could be a mismatch in taxonomy
  between Psychromonas and Neilla?
- “other” (other taxa) = 0.088%

Also note that based on the 16S analysis of gut content vs mucosa vs
feed pellet, *Lactobacillus* is likely introduced by the feed and
is just a transient member of the gut microbiota. This matches with the
taxonomy of the bin, *Lactobacillus johnsonii*, which is a
commonly used probiotic (at least in human foods).

#### Supp figure

```
# Supplementary figure 16S vs MAG results

ggplot(comparison, aes(ASV.abund, bin.abund, color = Genus))+
  geom_abline(slope = 1, intercept = 0, color = "grey50", linetype = 2, size = 1)+
  geom_point(size = 4)+
#  geom_text(aes(label = Genus), color = "black")+ #nudge_x = 0.2, nudge_y = -0.1
  scale_color_manual(values = c(gg_color_hue(11),"grey50"),
                     labels = c("Aliivibrio", "Alteromonadales", "Brevinema", "Carnobacterium",
                                "Clostridium", "Lactobacillus", "Mycoplasmataceae", "Neiella",
                                "Photobacterium", "Psychromonas", "Vibrionaceae", "Other"))+
  scale_x_continuous(limits = c(1e-5,1), trans = "log10", 
                     breaks = c(0.00001,0.0001,0.001,0.01,0.1,1), 
                     labels = c("0.001%","0.01%","0.1%","1%","10%","100%"))+
  scale_y_continuous(limits = c(1e-5,1), trans = "log10", 
                     breaks = c(0.00001,0.0001,0.001,0.01,0.1,1), 
                     labels = c("0.001%","0.01%","0.1%","1%","10%","100%"))+
  labs(x = "16S abundance", y = "MAG abundance")+
  theme_classic()+
  theme(panel.grid.major = element_blank(), panel.grid.minor = element_blank(), panel.background = element_blank(), 
        axis.line = element_line(colour = "black"), axis.title = element_text(size = 13), axis.text = element_text(color = "black", size = 12),
        legend.position = "right", legend.text = element_text(color = "black", size = 11), legend.title = element_text(color = "black", size = 13))
```

```
ggsave("figures/Supp_figure_16S_vs_MAG_abundance.png", units = "in", dpi = 600, width = 7, height = 5)
```

**Fig. S6.** Comparison of microbial relative abundances
in salmon gut content samples captured by MAG assembly versus 16S
profiling. The colours represent microbial genera, families, or orders
in the case of Alteromonadales, as defined in the legend. The dashed
line indicates hypothetical perfect concordance in relative abundances
between the MAG and 16S profiling methods. Relative abundances are shown
on a log10 scale. Generally, good agreement was observed for the most
common microbial taxa. However, Psychromonas and Clostridium were only
detected by MAG assembly (with relative abundances < 0.1%), while
Neiella was only detected by 16S profiling. Psychromonas and Neiella are
both of the order Alteromonadales and have somewhat similar relative
abundances in the MAG and 16S datasets, respectively, suggesting that
they may partly represent the same cluster of microbes that were
assigned different taxonomies at lower levels due to differences in the
databases used. ‘Other’ represents all 16S amplicon sequencing variants
(ASVs) that are not represented by the MAG catalogue and accounts for
approximately 0.1% of the total number of reads assigned to ASVs.

## MetaG community analysis

```
# Phyloseq object on filtered abundance
bin.filt <- dcast(bin.samples.abund, Fish.ID ~ MAG.ID, value.var = "Filtered.abundance")
row.names(bin.filt) <- bin.filt$Fish.ID
bin.filt <- bin.filt[rowSums(bin.filt[,-1]) > 0,]
bin.filt <- bin.filt[colSums(bin.filt[,-1]) > 0,]

row.names(bin.taxonomy) <- bin.taxonomy$MAG.ID
bin.taxa.ps <- tax_table(as.matrix(bin.taxonomy[,c(2:8)]))
bin.ps <- phyloseq(
  otu_table(bin.filt[,2:ncol(bin.filt)], taxa_are_rows = FALSE),
  sample_data(meta),
  bin.taxa.ps
)

# Log10 transformation
# min(subset(bin.samples.abund, Filtered.detected)$Filtered.abundance)
## 0.003079978 --> use 0.001 as pseudo-count
bin.ps.log <- transform_sample_counts(bin.ps, function(OTU) log10(OTU+0.001)) #log10 after pseudo-count
bin.df.log <- as.data.frame(otu_table(bin.ps.log))

bin.samples.abund$Log.filtered.abundance <- sapply(bin.samples.abund$Filtered.abundance, function(x) { log10(x + 0.001)})

# Phyloseq object on filtered detection (presence/absence)
bin.PA <- dcast(bin.samples.abund, Fish.ID ~ MAG.ID, value.var = "Filtered.detected")
row.names(bin.PA) <- bin.PA$Fish.ID
bin.PA <- bin.PA[rowSums(bin.PA[,-1]) > 0,]

bin.PA.ps <- microbiome::transform(bin.ps, transform = "pa")
```

### Ordination

**Ordination 1**: MAG abundances

```
# Using log10 of Filtered.abundance values

ord.euc <- ps_ordination_f(bin.ps.log, "NMDS", "euclidean", c(2:4), "output_ordinations/bin_filt_abund_log10_ps_nmds_euc.rds")

# stats
dist.euc <- phyloseq::distance(bin.ps.log, method = "euclidean")
perm.euc <- adonis2(dist.euc ~ Size.class + Tapeworm + Feed.Type, data = data.frame(sample_data(bin.ps.log)), method = "euclidean")
perm.euc
```

```
## Permutation test for adonis under reduced model
## Terms added sequentially (first to last)
## Permutation: free
## Number of permutations: 999
## 
## adonis2(formula = dist.euc ~ Size.class + Tapeworm + Feed.Type, data = data.frame(sample_data(bin.ps.log)), method = "euclidean")
##             Df SumOfSqs      R2      F Pr(>F)    
## Size.class   2    136.1 0.03564 7.7034  0.001 ***
## Tapeworm     3    217.7 0.05698 8.2109  0.001 ***
## Feed.Type    1     64.3 0.01684 7.2795  0.001 ***
## Residual   385   3402.2 0.89055                  
## Total      391   3820.4 1.00000                  
## ---
## Signif. codes:  0 '***' 0.001 '**' 0.01 '*' 0.05 '.' 0.1 ' ' 1
```

```
# format to print as table
perm.euc.df <- as.data.frame(perm.euc)
perm.euc.df$Variable <- row.names(perm.euc.df)
perm.euc.df$Dataset <- "MAG.abund"
row.names(perm.euc.df) <- NULL
```

Using *log10-transformed* filtered abundances and NMDS on
euclidean distances. NMDS stress: 0.2, k = 2. Overall size, cestode
index and feed type explain 10.9% of sample variation. Some tendency for
small, tapewormy fish to be on one side of D1 and large, tapeworm-less
fish to be on the other but not a strong association - however, wider
variation between samples within small, tapeworm-y fish, compared to
larger, healthier fish.

```
# ordination plots
p11 <-
plot_ordination(bin.ps.log, ord.euc$ord, type="samples", axes=c(1,2), color="Feed.Type")+
  geom_point(size=2)+
  stat_ellipse(type = "norm", size = 1, alpha = 0.5)+
  scale_color_manual(values = anno_colours$Feed.Type)+
  labs(color = "Feed type")+
  theme_bw()+
  theme(axis.text = element_text(color = "black"))

p12 <-
plot_ordination(bin.ps.log, ord.euc$ord, type="samples", axes=c(1,2), color="Size.class")+
  geom_point(size=2)+
  stat_ellipse(type = "norm", size = 1, alpha = 0.5)+
  scale_color_manual(values = anno_colours$Size.class)+
  labs(color = "Size class")+
  theme_bw()+
  theme(axis.text = element_text(color = "black"))

p13 <-
plot_ordination(bin.ps.log, ord.euc$ord, type="samples", axes=c(1,2), color="Tapeworm")+
  geom_point(size=2)+
  stat_ellipse(type = "norm", size = 1, alpha = 0.5)+
  scale_color_manual(values = anno_colours$Tapeworm)+
  labs(color = "Cestode index")+
  theme_bw()+
  theme(axis.text = element_text(color = "black"))
  
cowplot::plot_grid(p12, p13, nrow = 1)
```

**NMDS of MAG abundances** coloured by size class and
cestode index.

**Explore NMDS by bin taxonomy:**

```
bin.ps.log <- merge_phyloseq(bin.ps.log, sample_data(bin.df.log))

# names(bin.df.log)

p14 <-
plot_ordination(bin.ps.log, ord.euc$ord, type="samples", axes=c(1,2), color="MAG01_Mycoplasma_salmoninae")+
  geom_point(size=3)+
  scale_color_gradientn(colors = viridis::plasma(10))+
  labs(color = "Bin Abundance", title = "MAG01_Mycoplasma_salmoninae")+
  theme_bw()+
  theme(axis.text = element_text(color = "black"))

p15 <-
plot_ordination(bin.ps.log, ord.euc$ord, type="samples", axes=c(1,2), color="MAG06_Photobacterium_phosphoreum")+
  geom_point(size=3)+
  scale_color_gradientn(colors = viridis::plasma(10))+
  labs(color = "Bin Abundance", title = "MAG06_Photobacterium_phosphoreum")+
  theme_bw()+
  theme(axis.text = element_text(color = "black"))

p16 <-
plot_ordination(bin.ps.log, ord.euc$ord, type="samples", axes=c(1,2), color="MAG08_Aliivibrio_sp")+
  geom_point(size=3)+
  scale_color_gradientn(colors = viridis::plasma(10))+
  labs(color = "Bin Abundance", title = "MAG08_Aliivibrio_sp")+
  theme_bw()+
  theme(axis.text = element_text(color = "black"))

p17 <-
plot_ordination(bin.ps.log, ord.euc$ord, type="samples", axes=c(1,2), color="MAG02_Mycoplasma_sp")+
  geom_point(size=3)+
  scale_color_gradientn(colors = viridis::plasma(10))+
  labs(color = "Bin Abundance", title = "MAG02_Mycoplasma_sp")+
  theme_bw()+
  theme(axis.text = element_text(color = "black"))


cowplot::plot_grid(p15, p16, p14, p17, nrow = 2)
```

**NMDS of MAG abundances** coloured by bin abundances.
It’s clear that D2 separates highly abundant *M. salmoninae*
samples from vibrio-related samples, while D1 separates
*Photobacterium* dominated samples from *Aliivibrio*
dominated samples (in the upper part of D2). Some of the other
mycoplasmas seem more abundant on one side of D1 (in the lower part of
D2).

**Ordination 2**: MAG detection

```
# ordination on jaccard binary distances

distPA.jac <- vegdist(bin.PA[,-1], method = "jaccard", binary = T)
ordPA.jac <- ps_ordination_f(distPA.jac, "NMDS", "jaccard", c(2:4), "output_ordinations/bin_PA_NMDS_jac.rds")

ordPA.jac.scores <- as.data.frame(scores(ordPA.jac$ord, display = "site"))
ordPA.jac.scores$Fish.ID <- bin.PA$Fish.ID
ordPA.jac.scores <- merge(ordPA.jac.scores, meta[,c("Fish.ID",metavar.fish)], by = "Fish.ID")

perm.PAjac <- adonis2(distPA.jac ~ Tapeworm + Size.class + Feed.Type, data = ordPA.jac.scores, method = "jaccard")
perm.PAjac
```

```
## Permutation test for adonis under reduced model
## Terms added sequentially (first to last)
## Permutation: free
## Number of permutations: 999
## 
## adonis2(formula = distPA.jac ~ Tapeworm + Size.class + Feed.Type, data = ordPA.jac.scores, method = "jaccard")
##             Df SumOfSqs      R2      F Pr(>F)  
## Tapeworm     3    0.410 0.00488 0.6376  0.862  
## Size.class   2    0.594 0.00707 1.3836  0.179  
## Feed.Type    1    0.415 0.00494 1.9340  0.073 .
## Residual   385   82.608 0.98311                
## Total      391   84.027 1.00000                
## ---
## Signif. codes:  0 '***' 0.001 '**' 0.01 '*' 0.05 '.' 0.1 ' ' 1
```

```
# format to print as table
perm.PAjac.df <- as.data.frame(perm.PAjac)
perm.PAjac.df$Variable <- row.names(perm.PAjac.df)
perm.PAjac.df$Dataset <- "MAG.det"
row.names(perm.PAjac.df) <- NULL
```

Using **presence/absence** values (after filtering) and
NMDS on Jaccard distances. NMDS stress: 0.135, k = 3. Overall size,
cestode index and feed type explain 1.7% of sample variation. D1 seems
to separate low (0, 1) and high (2, 3) levels of tapeworm. No strong
trends by size or feed.

```
p21 <-
ggplot(ordPA.jac.scores,aes(NMDS1, NMDS2, color=Feed.Type))+
  geom_vline(xintercept = 0, linetype = 3, size = 1, color = "grey42") + geom_hline(yintercept = 0, linetype = 3, size = 1, color = "grey42")+
  geom_point(size = 3)+
  scale_color_manual(values = anno_colours$Feed.Type)+
  stat_ellipse(type = "norm", size = 1, alpha = 0.5)+
  theme_bw()+ 
  theme(panel.grid = element_blank(), legend.position = "right",
        axis.text = element_text(size = 12, colour = "black"), axis.title = element_text(size = 13), panel.border = element_rect(size = 1.2))

p22 <-
ggplot(ordPA.jac.scores,aes(NMDS1, NMDS2, color=Size.class))+
  geom_vline(xintercept = 0, linetype = 3, size = 1, color = "grey42") + geom_hline(yintercept = 0, linetype = 3, size = 1, color = "grey42")+
  geom_point(size = 3)+
  scale_color_manual(values = anno_colours$Size.class)+
  stat_ellipse(type = "norm", size = 1, alpha = 0.5)+
  theme_bw()+ 
  theme(panel.grid = element_blank(), legend.position = "right",
        axis.text = element_text(size = 12, colour = "black"), axis.title = element_text(size = 13), panel.border = element_rect(size = 1.2))

p23 <-
ggplot(ordPA.jac.scores,aes(NMDS1, NMDS2, color=Tapeworm))+
  geom_vline(xintercept = 0, linetype = 3, size = 1, color = "grey42") + geom_hline(yintercept = 0, linetype = 3, size = 1, color = "grey42")+
  geom_point(size = 3)+
  scale_color_manual(values = anno_colours$Tapeworm)+
  stat_ellipse(type = "norm", size = 1, alpha = 0.5)+
  theme_bw()+ 
  theme(panel.grid = element_blank(), legend.position = "right",
        axis.text = element_text(size = 12, colour = "black"), axis.title = element_text(size = 13), panel.border = element_rect(size = 1.2))

cowplot::plot_grid(p21, p22, p23, nrow = 3)
```

**NMDS of MAG presence/absence** coloured by feed type,
size class and cestode index.

**Explore NMDS by bin taxonomy:**

```
ordPA.jac.scores <- merge(ordPA.jac.scores, bin.PA, by = "Fish.ID")

p24 <-
ggplot(ordPA.jac.scores,aes(NMDS1, NMDS2, color=MAG02_Mycoplasma_sp))+
  geom_vline(xintercept = 0, linetype = 3, size = 1, color = "grey42") + geom_hline(yintercept = 0, linetype = 3, size = 1, color = "grey42")+
  geom_point(size = 3)+
  scale_color_manual(values = anno_colours$MAG.det)+
  labs(color = "Bin detected", title = "MAG02_Mycoplasma_sp")+
  stat_ellipse(type = "norm", size = 1, alpha = 0.5)+
  theme_bw()+ 
  theme(panel.grid = element_blank(), legend.position = "right",
        axis.text = element_text(size = 12, colour = "black"), axis.title = element_text(size = 13), panel.border = element_rect(size = 1.2))

p25 <-
ggplot(ordPA.jac.scores,aes(NMDS1, NMDS2, color=MAG06_Photobacterium_phosphoreum))+
  geom_vline(xintercept = 0, linetype = 3, size = 1, color = "grey42") + geom_hline(yintercept = 0, linetype = 3, size = 1, color = "grey42")+
  geom_point(size = 3)+
  scale_color_manual(values = anno_colours$MAG.det)+
  labs(color = "Bin detected", title = "MAG06_Photobacterium_phosphoreum")+
  stat_ellipse(type = "norm", size = 1, alpha = 0.5)+
  theme_bw()+ 
  theme(panel.grid = element_blank(), legend.position = "right",
        axis.text = element_text(size = 12, colour = "black"), axis.title = element_text(size = 13), panel.border = element_rect(size = 1.2))

p26 <-
ggplot(ordPA.jac.scores,aes(NMDS1, NMDS2, color=MAG08_Aliivibrio_sp))+
  geom_vline(xintercept = 0, linetype = 3, size = 1, color = "grey42") + geom_hline(yintercept = 0, linetype = 3, size = 1, color = "grey42")+
  geom_point(size = 3)+
  scale_color_manual(values = anno_colours$MAG.det)+
  labs(color = "Bin detected", title = "MAG08_Aliivibrio_sp")+
  stat_ellipse(type = "norm", size = 1, alpha = 0.5)+
  theme_bw()+ 
  theme(panel.grid = element_blank(), legend.position = "right",
        axis.text = element_text(size = 12, colour = "black"), axis.title = element_text(size = 13), panel.border = element_rect(size = 1.2))

p27 <-
ggplot(ordPA.jac.scores,aes(NMDS1, NMDS3, color=MAG11_Brevinema_sp))+
  geom_vline(xintercept = 0, linetype = 3, size = 1, color = "grey42") + geom_hline(yintercept = 0, linetype = 3, size = 1, color = "grey42")+
  geom_point(size = 3)+
  scale_color_manual(values = anno_colours$MAG.det)+
  labs(color = "Bin detected", title = "MAG11_Brevinema_sp")+
  stat_ellipse(type = "norm", size = 1, alpha = 0.5)+
  theme_bw()+ 
  theme(panel.grid = element_blank(), legend.position = "right",
        axis.text = element_text(size = 12, colour = "black"), axis.title = element_text(size = 13), panel.border = element_rect(size = 1.2))

cowplot::plot_grid(p25, p26, p24, p27, nrow = 2)
```

### Microbime phenotypes

Based on above patterns, can define a number of microbiome
phenotypes:

- *M. salmoninae* abundance (no point doing presence/absence as
  detected in most samples)
- *Mycoplasma* bins abundance (bins detected in > 50
  samples)
- *Photobacterium* bins abundances (bins detected in > 50
  samples)
- *Aliivirbio* bins abundances (bins detected in > 50
  samples)
- *Mycoplasma* spp. presence/absence (excluding *M.
  salmoninae*)
- *Photobacterium* spp. presence/absence
- *Aliivibrio* spp. presence/absence
- *Brevinema* bin presence/absence
- *M. salmoninae* high abundance (binary), based on 3rd
  quartile (> 1.7 (log10(50X mean coverage)))
- also include all MAGs with detection > 10

```
# Phenotype definitions
temp.bin.log <- bin.df.log[,c("MAG01_Mycoplasma_salmoninae","MAG02_Mycoplasma_sp",
                              "MAG03_Mycoplasma_sp","MAG04_Mycoplasma_sp","MAG05_Mycoplasma_sp",
                              "MAG06_Photobacterium_phosphoreum","MAG08_Aliivibrio_sp")]
names(temp.bin.log) <- c("Bin.ab.MAG01","Bin.ab.MAG02","Bin.ab.MAG03","Bin.ab.MAG04",
                         "Bin.ab.MAG05","Bin.ab.MAG06","Bin.ab.MAG08")
temp.bin.log$Fish.ID <- row.names(bin.df.log)

meta <- merge(meta, temp.bin.log, by = "Fish.ID", all = T)
rm(temp.bin.log)

bins.included.pa <- names(bin.PA[,-1])[which(colSums(bin.PA[,-1]) > 10 & colSums(bin.PA[,-1]) < (nrow(bin.PA) - 10))]

temp.bin.pa <- bin.PA[,c("Fish.ID",bins.included.pa)]
names(temp.bin.pa) <- c("Fish.ID","Bin.pa.MAG02","Bin.pa.MAG03","Bin.pa.MAG04",
                        "Bin.pa.MAG05","Bin.pa.MAG06","Bin.pa.MAG07","Bin.pa.MAG08",
                        "Bin.pa.MAG09","Bin.pa.MAG11","Bin.pa.MAG12","Bin.pa.MAG13")
temp.bin.pa$Bin.pa.Myco <- rowSums(bin.PA[,c("MAG02_Mycoplasma_sp","MAG03_Mycoplasma_sp",
                                             "MAG04_Mycoplasma_sp","MAG05_Mycoplasma_sp" )]) > 0
temp.bin.pa$Bin.pa.Photo <- rowSums(bin.PA[,c("MAG06_Photobacterium_phosphoreum","MAG07_Photobacterium_iliopiscarium")]) > 0
temp.bin.pa$Bin.pa.Ali <- rowSums(bin.PA[,c("MAG08_Aliivibrio_sp","MAG09_Aliivibrio_sp","MAG10_Aliivibrio_salmonicida")]) > 0

meta <- merge(meta, temp.bin.pa, by = "Fish.ID", all = T)
rm(temp.bin.pa)

summary(meta$Bin.ab.MAG01)
```

```
##    Min. 1st Qu.  Median    Mean 3rd Qu.    Max.    NA's 
##  -3.000   1.688   1.778   1.612   1.787   1.789      68
```

```
meta$Bin.pa.Msal.high <- meta$Bin.ab.MAG01 > 1.7

# add first 2 ordination dimensions (abundance-based) to meta for downstream use, e.g. GWAS
meta$Bin.ab.NMDS1 <- sapply(meta$Fish.ID, function(x){
  nmds <- as.data.frame(scores(ord.euc$ord, display = "site"))
  if(x %in% row.names(nmds)){
    nmds[x,"NMDS1"]
  } else {
    NA
  }
})
meta$Bin.ab.NMDS2 <- sapply(meta$Fish.ID, function(x){
  nmds <- as.data.frame(scores(ord.euc$ord, display = "site"))
  if(x %in% row.names(nmds)){
    nmds[x,"NMDS2"]
  } else {
    NA
  }
})
```

### Supp figure ordination

```
paper.supp.1 <-
plot_ordination(bin.ps.log, ord.euc$ord, type="samples", axes=c(1,2), color="Feed.Type")+
  geom_vline(xintercept = 0, linetype = 3, size = 1, color = "grey42") + geom_hline(yintercept = 0, linetype = 3, size = 1, color = "grey42")+
  geom_point(size = 3)+
  stat_ellipse(type = "norm", size = 1, alpha = 0.5)+
  scale_color_manual(values = anno_colours$Feed.Type)+
  labs(color = "Feed type")+
  theme_bw()+
  theme(axis.text = element_text(size = 12, color = "black"), axis.title = element_text(size = 13), 
        legend.text = element_text(size = 12, color = "black"), legend.title = element_text(size = 12))

paper.supp.2 <-
plot_ordination(bin.ps.log, ord.euc$ord, type="samples", axes=c(1,2), color="Size.class")+
  geom_vline(xintercept = 0, linetype = 3, size = 1, color = "grey42") + geom_hline(yintercept = 0, linetype = 3, size = 1, color = "grey42")+
  geom_point(size = 3)+
  stat_ellipse(type = "norm", size = 1, alpha = 0.5)+
  scale_color_manual(values = anno_colours$Size.class)+
  labs(color = "Size class")+
  theme_bw()+
  theme(axis.text = element_text(size = 12, color = "black"), axis.title = element_text(size = 13), 
        legend.text = element_text(size = 12, color = "black"), legend.title = element_text(size = 12))

paper.supp.3 <-
plot_ordination(bin.ps.log, ord.euc$ord, type="samples", axes=c(1,2), color="Tapeworm")+
geom_vline(xintercept = 0, linetype = 3, size = 1, color = "grey42") + geom_hline(yintercept = 0, linetype = 3, size = 1, color = "grey42")+
  geom_point(size = 3)+
  stat_ellipse(type = "norm", size = 1, alpha = 0.5)+
  scale_color_manual(values = anno_colours$Tapeworm)+
  labs(color = "Cestode index", title = "MAG abundance")+
  theme_bw()+
  theme(axis.text = element_text(size = 12, color = "black"), axis.title = element_text(size = 13), 
        legend.text = element_text(size = 12, color = "black"), legend.title = element_text(size = 12),
        plot.title = element_text(hjust = 0.5))
  

paper.supp.4 <-
ggplot(ordPA.jac.scores,aes(NMDS1, NMDS2, color=Feed.Type))+
  geom_vline(xintercept = 0, linetype = 3, size = 1, color = "grey42") + geom_hline(yintercept = 0, linetype = 3, size = 1, color = "grey42")+
  geom_point(size = 3)+
  scale_color_manual(values = anno_colours$Feed.Type)+
  stat_ellipse(type = "norm", size = 1, alpha = 0.5)+
  labs(color = "Feed type")+
  theme_bw()+ 
  theme(axis.text = element_text(size = 12, colour = "black"), axis.title = element_text(size = 13), 
        legend.text = element_text(size = 12, color = "black"), legend.title = element_text(size = 12))

paper.supp.5 <-
ggplot(ordPA.jac.scores,aes(NMDS1, NMDS2, color=Size.class))+
  geom_vline(xintercept = 0, linetype = 3, size = 1, color = "grey42") + geom_hline(yintercept = 0, linetype = 3, size = 1, color = "grey42")+
  geom_point(size = 3)+
  scale_color_manual(values = anno_colours$Size.class)+
  stat_ellipse(type = "norm", size = 1, alpha = 0.5)+
  labs(color = "Size class")+
  theme_bw()+ 
  theme(axis.text = element_text(size = 12, colour = "black"), axis.title = element_text(size = 13), 
        legend.text = element_text(size = 12, color = "black"), legend.title = element_text(size = 12))

paper.supp.6 <-
ggplot(ordPA.jac.scores,aes(NMDS1, NMDS2, color=Tapeworm))+
  geom_vline(xintercept = 0, linetype = 3, size = 1, color = "grey42") + geom_hline(yintercept = 0, linetype = 3, size = 1, color = "grey42")+
  geom_point(size = 3)+
  scale_color_manual(values = anno_colours$Tapeworm)+
  stat_ellipse(type = "norm", size = 1, alpha = 0.5)+
  labs(color = "Cestode index", title = "MAG presence/absence")+
  theme_bw()+ 
  theme(axis.text = element_text(size = 12, colour = "black"), axis.title = element_text(size = 13), 
        legend.text = element_text(size = 12, color = "black"), legend.title = element_text(size = 12),
        plot.title = element_text(hjust = 0.5))

cowplot::plot_grid(paper.supp.3, paper.supp.6, paper.supp.2, paper.supp.5, paper.supp.1, paper.supp.4, 
                   nrow = 3, align = "hv", axis = "tblr", labels = c("A","B","C","D","E","F"))
```

```
ggsave("figures/Supp_figure_metag_ordinations.png", units = "in", dpi = 600, width = 10, height = 12)
```

**Fig. S1.** Ordination (NMDS) of metagenomes, based on
MAG abundance (**A, C, E**) and MAG presence/absence
(**B, D, F**). Individuals are coloured by cestode index
(A, B), size class (C, D) or feed type (E, F). For MAG abundance (A, C,
E), the NMDS was generated using Euclidean distances (NMDS stress:
0.200, k = 2). Based on PERMANOVA results, cestode index contributed to
5.70% of the variation in abundance (F = 8.21, p = 0.001), size class to
3.56% (F = 7.70, p = 0.001), and feed type to 1.68% (F = 7.28, p =
0.001). For presence/absence (B, D, F), the NMDS was generated from
Jaccard distances (NMDS stress: 0.135, k = 3). In the PERMANOVA, all
three phenotypes explained very little variation in MAG detection.
Detailed results from the PERMANOVAs are presented in Table S1.

### Figure 2

Figure 2A: Heatmap of abundances.

```
sample.anno <- data.frame(
  Size.class=sapply(row.names(bin.df.log), function(x){
    meta[which(meta$Fish.ID == x),"Size.class"]
  }),
  Tapeworm=sapply(row.names(bin.df.log), function(x){
    meta[which(meta$Fish.ID == x),"Tapeworm"]
  })
)

hm.magorder <- names(bin.df.log)

#change log mins to NA values for heatmap
log.pseudozeros <- sapply(row.names(bin.df.log), function(x){min(bin.df.log[x,])})
bin.log.nas <- as.matrix(t(bin.df.log))
for (s in colnames(bin.log.nas)) {
  bin.log.nas[which(bin.log.nas[,s] == log.pseudozeros[s]),s] <- as.double("NA")
}


## order by size and tapeworm

hm.bin.log.sampleordered.by.meta <- meta[order(meta$Tapeworm.bin, 
                                               factor(meta$Size.class,levels = rev(c("Small","Medium","Large")), ordered = T),
                                               meta$Bin.pa.Myco, 
                                               meta$Bin.pa.Photo),"Fish.ID"]
hm.bin.log.sampleordered.by.meta <- hm.bin.log.sampleordered.by.meta[which(hm.bin.log.sampleordered.by.meta %in% colnames(bin.log.nas))]

sample.anno2 <- data.frame(
  Size.class=sapply(row.names(bin.df.log), function(x){
    meta[which(meta$Fish.ID == x),"Size.class"]
  }),
  Tapeworm.bin=sapply(row.names(bin.df.log), function(x){
    meta[which(meta$Fish.ID == x),"Tapeworm.bin"]
  })
)
sample.anno2$Cestode <- ifelse(sample.anno2$Tapeworm.bin, "Yes", "No")
anno_colours_hm2 <- list(
  Size.class=c(Small=gg_color_hue(3)[3], Medium=gg_color_hue(3)[2], Large=gg_color_hue(3)[1]),
  Cestode=c(No="#440154FF", Yes="#FDE725FF")
)
```

```
## In order to save png, run in terminal, not in markdown!
# setwd("C:/Users/jaelleb/WORK/R_analyses_Cdrive/Brealey_etal_salmon_multiomics/Brealey_etal_salmon_multiomics/")
# png("figures/Figure_metag_heatmap_230110.png", units = "in", res = 600, width = 7, height = 4)
# pheatmap call
# dev.off()
pheatmap(bin.log.nas[hm.magorder,hm.bin.log.sampleordered.by.meta],
         annotation_col = sample.anno2[,c("Size.class","Cestode")], annotation_colors = anno_colours_hm2,
         cluster_rows = F, cluster_cols = F, show_colnames = F, na_col = "#b3b3b3")
```

**Fig. 2**. MAG abundance and frequency of detection in
the Atlantic salmon gut metagenome. (**A**) Normalised MAG
abundances per sample (log-transformed). Samples are ordered and
annotated by cestode detection and size class, while MAGs are ordered by
taxonomy and frequency of detection. MAGs that were not detected in a
sample are shown in grey. (**B**) MAG detection in all
samples (see below table), separated by size class or cestode detection.
MAGs with more frequent detection (% of samples) are coloured in yellow,
MAGs with lower detection are coloured in red.

```
bin.table <- data.frame(
  Bin=names(bin.PA[,-1]),
  Bin.detection=colSums(bin.PA[,-1]),
  Bin.det.Small=colSums(bin.PA[which(bin.PA$Fish.ID %in% meta[which(meta$Size.class=="Small"),"Fish.ID"]),-1]),
  Bin.det.Medium=colSums(bin.PA[which(bin.PA$Fish.ID %in% meta[which(meta$Size.class=="Medium"),"Fish.ID"]),-1]),
  Bin.det.Large=colSums(bin.PA[which(bin.PA$Fish.ID %in% meta[which(meta$Size.class=="Large"),"Fish.ID"]),-1]),
  Bin.det.Tapeworm=colSums(bin.PA[which(bin.PA$Fish.ID %in% meta[which(meta$Tapeworm.bin),"Fish.ID"]),-1]),
  Bin.det.NoTapeworm=colSums(bin.PA[which(bin.PA$Fish.ID %in% meta[which(!meta$Tapeworm.bin),"Fish.ID"]),-1])
)
bin.table <- cbind.data.frame(bin.table, data.frame(
  Bin.percent=round(bin.table$Bin.detection / nrow(bin.PA) * 100, digits = 1),
  Bin.per.Small=round(bin.table$Bin.det.Small / nrow(bin.PA[which(bin.PA$Fish.ID %in% meta[which(meta$Size.class=="Small"),"Fish.ID"]),]) * 100, digits = 1),
  Bin.per.Medium=round(bin.table$Bin.det.Medium / nrow(bin.PA[which(bin.PA$Fish.ID %in% meta[which(meta$Size.class=="Medium"),"Fish.ID"]),]) * 100, digits = 1),
  Bin.per.Large=round(bin.table$Bin.det.Large / nrow(bin.PA[which(bin.PA$Fish.ID %in% meta[which(meta$Size.class=="Large"),"Fish.ID"]),]) * 100, digits = 1),
  Bin.per.Tapeworm=round(bin.table$Bin.det.Tapeworm / nrow(bin.PA[which(bin.PA$Fish.ID %in% meta[which(meta$Tapeworm.bin),"Fish.ID"]),]) * 100, digits = 1),
  Bin.per.NoTapeworm=round(bin.table$Bin.det.NoTapeworm / nrow(bin.PA[which(bin.PA$Fish.ID %in% meta[which(!meta$Tapeworm.bin),"Fish.ID"]),]) * 100, digits = 1)
))

bin.table[,c(1,2,8:13)] %>%
  kbl(row.names = FALSE) %>%
  kable_styling(bootstrap_options = c("striped","hover")) %>%
  column_spec(3, background = spec_color(bin.table[hm.magorder,"Bin.percent"], option = "magma", begin = 0.5, alpha = 0.5, scale_from = c(0,100)))
```

| Bin | Bin.detection | Bin.percent | Bin.per.Small | Bin.per.Medium | Bin.per.Large | Bin.per.Tapeworm | Bin.per.NoTapeworm |
| --- | --- | --- | --- | --- | --- | --- | --- |
| MAG01\_Mycoplasma\_salmoninae | 389 | 99.2 | 97.3 | 100.0 | 100.0 | 99.1 | 100.0 |
| MAG02\_Mycoplasma\_sp | 215 | 54.8 | 60.4 | 53.7 | 51.3 | 63.1 | 18.1 |
| MAG03\_Mycoplasma\_sp | 86 | 21.9 | 22.5 | 22.8 | 20.2 | 25.9 | 4.2 |
| MAG04\_Mycoplasma\_sp | 73 | 18.6 | 15.3 | 21.0 | 18.5 | 22.2 | 2.8 |
| MAG05\_Mycoplasma\_sp | 49 | 12.5 | 18.0 | 12.3 | 7.6 | 15.3 | 0.0 |
| MAG06\_Photobacterium\_phosphoreum | 183 | 46.7 | 69.4 | 42.6 | 31.1 | 47.8 | 41.7 |
| MAG07\_Photobacterium\_iliopiscarium | 20 | 5.1 | 3.6 | 6.2 | 5.0 | 4.1 | 9.7 |
| MAG08\_Aliivibrio\_sp | 72 | 18.4 | 16.2 | 19.8 | 18.5 | 16.6 | 26.4 |
| MAG09\_Aliivibrio\_sp | 27 | 6.9 | 9.9 | 8.0 | 2.5 | 7.5 | 4.2 |
| MAG10\_Aliivibrio\_salmonicida | 7 | 1.8 | 2.7 | 1.9 | 0.8 | 2.2 | 0.0 |
| MAG11\_Brevinema\_sp | 104 | 26.5 | 20.7 | 25.3 | 33.6 | 24.7 | 34.7 |
| MAG12\_Carnobacterium\_maltaromaticum | 57 | 14.5 | 9.0 | 14.8 | 19.3 | 17.5 | 1.4 |
| MAG13\_Lactobacillus\_johnsonii | 32 | 8.2 | 6.3 | 8.6 | 9.2 | 8.4 | 6.9 |
| MAG14\_Clostridium\_ljungdahlii | 2 | 0.5 | 0.9 | 0.6 | 0.0 | 0.3 | 1.4 |
| MAG15\_Psychromonas\_sp | 2 | 0.5 | 0.0 | 1.2 | 0.0 | 0.6 | 0.0 |

### Alpha diversity

```
# Hill Numbers (package hillR)
# really great primer on Hill's numbers framework: https://onlinelibrary.wiley.com/doi/full/10.1111/1755-0998.13014
sample_data(bin.ps)$Bin.alpha.hill1 <- hill_taxa(otu_table(bin.ps), q = 1)
meta$Bin.alpha.hill1 <- sapply(meta$Fish.ID, function(x) {
  if(x %in% sample_data(bin.ps)$Fish.ID) {
    as.numeric(sample_data(bin.ps)[which(sample_data(bin.ps)$Fish.ID == x),"Bin.alpha.hill1"])
  } else {
    NA
  }
})
```

```
ggplot(meta, aes(Size.class, Bin.alpha.hill1))+
  geom_boxplot(aes(fill = Tapeworm.bin), alpha = 0.25, outlier.colour = NA)+
  geom_point(aes(fill = Tapeworm.bin), shape = 21, color = "black", size = 3, position = position_jitterdodge(jitter.width = 0.2))+
  scale_color_manual(values = as.character(anno_colours$Tapeworm.bin))+
  scale_fill_manual(values = anno_colours$Tapeworm.bin)+
  labs(x = "Size class", y = "Hill's shannon index", color = "Cestode detected", fill = "Cestode detected")+
  theme_classic()+
  theme(axis.text = element_text(color = "black", size = 12), axis.title = element_text(size = 13),
        legend.text = element_text(size = 12, color = "black"), legend.title = element_text(size = 12))
```

```
ggsave("figures/Supp_figure_metag_alphadiv.png", units = "in", dpi = 600, width = 7, height = 4)
```

**Fig. S5**. Alpha diversity (Hill’s Shannon index) of
metagenomes, based on MAG abundance, stratified by cestode detection and
size class.

**Table S3**. Linear model of metagenome alpha diversity
differences among fish phenotypes.

```
# Statistics for figure:
summary(lm(Bin.alpha.hill1 ~ Tapeworm.bin + Size.class + Feed.Type + metag.sequencing.depth, data = meta[which(meta$Metagenome),]))
```

```
## 
## Call:
## lm(formula = Bin.alpha.hill1 ~ Tapeworm.bin + Size.class + Feed.Type + 
##     metag.sequencing.depth, data = meta[which(meta$Metagenome), 
##     ])
## 
## Residuals:
##      Min       1Q   Median       3Q      Max 
## -0.43836 -0.22368 -0.10931  0.04793  3.02542 
## 
## Coefficients:
##                          Estimate Std. Error t value Pr(>|t|)    
## (Intercept)             1.129e+00  5.644e-02  20.000  < 2e-16 ***
## Tapeworm.binTRUE        1.263e-01  5.130e-02   2.461   0.0143 *  
## Size.class.L           -1.518e-01  3.778e-02  -4.019 7.02e-05 ***
## Size.class.Q           -1.893e-02  3.291e-02  -0.575   0.5655    
## Feed.TypeFeed2          8.401e-02  4.026e-02   2.087   0.0376 *  
## metag.sequencing.depth -1.229e-09  1.176e-09  -1.045   0.2966    
## ---
## Signif. codes:  0 '***' 0.001 '**' 0.01 '*' 0.05 '.' 0.1 ' ' 1
## 
## Residual standard error: 0.3884 on 386 degrees of freedom
## Multiple R-squared:  0.08109,    Adjusted R-squared:  0.06918 
## F-statistic: 6.812 on 5 and 386 DF,  p-value: 4.186e-06
```

### Differential abundance testing

**MAG abundance associations** with Maaslin2. Size,
tapeworm and feed type comparisons (while controlling for sequencing
depth and sampling date). `Filtered.abundance` is
log-transformed by Maaslin2 before GLMs.

```
# size class & tapeworm presence/absence
maas.mag.abund <- run_maaslin2(bin.ps, "output_maaslin2/MAGv6_filt_abund",
                               c("Size.class","Feed.Type","Tapeworm.bin","metag.sequencing.depth"),
                               r_eff = c("Sampling.Date"), reference = c("Size.class,Small"),
                               n = "none", t = "LOG")

maas.mag.abund <- maas.mag.abund[order(maas.mag.abund$metadata,maas.mag.abund$value,maas.mag.abund$feature),]
```

**Table S4A.** Associations (FDR < 0.05) between fish
phenotypes and metagenomes, based on MAG abundance (n = 392).

```
maas.mag.abund[which(maas.mag.abund$qval < 0.05 & !maas.mag.abund$value %in% c(".C",".Q")),c(1:5,9)] %>%
  kbl(row.names = FALSE) %>%
  kable_styling(bootstrap_options = c("striped","hover")) %>%
  column_spec(4, color = "black", 
              background = spec_color(maas.mag.abund[which(maas.mag.abund$qval < 0.05 & 
                                                             !maas.mag.abund$value %in% c(".C",".Q")),"coef"], 
                                      option = "viridis", scale_from = c(-3.2,3.2), alpha = 0.5))
```

| feature | metadata | value | coef | stderr | qval |
| --- | --- | --- | --- | --- | --- |
| MAG08\_Aliivibrio\_sp | Feed.Type | Feed2 | 1.8296022 | 0.3075887 | 0.0000001 |
| MAG01\_Mycoplasma\_salmoninae | Size.class | .L | 0.4958303 | 0.1224675 | 0.0007005 |
| MAG06\_Photobacterium\_phosphoreum | Size.class | .L | -2.9734282 | 0.4003972 | 0.0000000 |
| MAG02\_Mycoplasma\_sp | Tapeworm.bin | TRUE | 2.3186408 | 0.3823823 | 0.0000001 |
| MAG03\_Mycoplasma\_sp | Tapeworm.bin | TRUE | 0.8924124 | 0.2419988 | 0.0023288 |
| MAG04\_Mycoplasma\_sp | Tapeworm.bin | TRUE | 0.5416822 | 0.1696652 | 0.0114495 |
| MAG05\_Mycoplasma\_sp | Tapeworm.bin | TRUE | 0.6654650 | 0.2233917 | 0.0197782 |
| MAG12\_Carnobacterium\_maltaromaticum | Tapeworm.bin | TRUE | 0.5906507 | 0.2034125 | 0.0219353 |

Feed type does have some effect (especially with abundance of
*Aliivibrio* MAG08) so should be controlled for in all analyses.
Larger fish are associated with higher *M. salmoninae* abundance
and lower *Photobacterium phosphoreum* abundances. Five bins
(four *Mycoplasma* related + one *Carnobacterium*) show
increased abundance when tapeworm is present. It is interesting to note
that two of these *Mycoplasma* bins are in the same species
cluster as MAGs recovered from the cestode endomicrobiome (CEseq1 =
MAG02 & CEseq7 = MAG03).

### Differential detection testing

**MAG detection (presence/absence) associations** with
Maaslin2:
Size, tapeworm and feed type comparisons (while controlling for
sequencing depth and sampling date).

```
# size class & tapeworm presence/absence
maas.mag.pa <- run_maaslin2(bin.PA.ps, "output_maaslin2/MAGv6_filt_PA",
                            c("Size.class","Feed.Type","Tapeworm.bin","metag.sequencing.depth"),
                            r_eff = c("Sampling.Date"), reference = c("Size.class,Small"), 
                            n = "none", t = "none")

maas.mag.pa <- maas.mag.pa[order(maas.mag.pa$metadata,maas.mag.pa$value,maas.mag.pa$feature),]
```

**Table S4B.** Associations (FDR < 0.05) between fish
phenotypes and metagenomes, based on MAG presence/absence (n = 392).

```
maas.mag.pa[which(maas.mag.pa$qval < 0.05 & !maas.mag.pa$value %in% c(".C",".Q")),c(1:5,9)] %>%
  kbl(row.names = FALSE) %>%
  kable_styling(bootstrap_options = c("striped","hover")) %>%
  column_spec(4, color = "black", 
              background = spec_color(maas.mag.pa[which(maas.mag.pa$qval < 0.05 & 
                                                          !maas.mag.pa$value %in% c(".C",".Q")),"coef"], 
                                      option = "viridis", scale_from = c(-3.2,1), alpha = 0.5))
```

| feature | metadata | value | coef | stderr | qval |
| --- | --- | --- | --- | --- | --- |
| MAG08\_Aliivibrio\_sp | Feed.Type | Feed2 | 0.2305864 | 0.0384560 | 0.0000001 |
| MAG02\_Mycoplasma\_sp | metag.sequencing.depth | metag.sequencing.depth | 0.0690382 | 0.0246628 | 0.0245251 |
| MAG04\_Mycoplasma\_sp | metag.sequencing.depth | metag.sequencing.depth | 0.0702866 | 0.0201973 | 0.0035922 |
| MAG11\_Brevinema\_sp | metag.sequencing.depth | metag.sequencing.depth | 0.0735418 | 0.0233355 | 0.0088011 |
| MAG06\_Photobacterium\_phosphoreum | Size.class | .L | -0.2910733 | 0.0463049 | 0.0000000 |
| MAG02\_Mycoplasma\_sp | Tapeworm.bin | TRUE | 0.4559046 | 0.0609881 | 0.0000000 |
| MAG03\_Mycoplasma\_sp | Tapeworm.bin | TRUE | 0.2229428 | 0.0538163 | 0.0004748 |
| MAG04\_Mycoplasma\_sp | Tapeworm.bin | TRUE | 0.1893006 | 0.0488197 | 0.0011175 |
| MAG05\_Mycoplasma\_sp | Tapeworm.bin | TRUE | 0.1420535 | 0.0427968 | 0.0055629 |
| MAG12\_Carnobacterium\_maltaromaticum | Tapeworm.bin | TRUE | 0.1718165 | 0.0453788 | 0.0013295 |

Save supplementary Maaslin2 results as a table.

```
# MAG abundances: cestode presence + size class
if(!file.exists("tables/Supp_table_metag_maaslin_abund_res_230109.csv")){
  paper.supp.tab.maas.mag <- maas.mag.abund[which(maas.mag.abund$qval < 0.2),]
  names(paper.supp.tab.maas.mag)[1] <- "MAG.ID"
  paper.supp.tab.maas.mag$metadata <- gsub("Tapeworm.bin","Cestode.present",paper.supp.tab.maas.mag$metadata)
  paper.supp.tab.maas.mag$metadata <- gsub("metag.sequencing.depth","sequencing.depth",paper.supp.tab.maas.mag$metadata)
  write.csv(paper.supp.tab.maas.mag[,c("MAG.ID","metadata","value","coef","stderr","N","N.not.0","pval","qval")],
            "tables/Supp_table_metag_maaslin_abund_res_230109.csv",
            row.names = F, quote = F)
}
# MAG frequency: cestode presence + size class
if(!file.exists("tables/Supp_table_metag_maaslin_freq_res_230109.csv")){
  paper.supp.tab.maas.mag <- maas.mag.pa[which(maas.mag.pa$qval < 0.2),]
  names(paper.supp.tab.maas.mag)[1] <- "MAG.ID"
  paper.supp.tab.maas.mag$metadata <- gsub("Tapeworm.bin","Cestode.present",paper.supp.tab.maas.mag$metadata)
  paper.supp.tab.maas.mag$metadata <- gsub("metag.sequencing.depth","sequencing.depth",paper.supp.tab.maas.mag$metadata)
  write.csv(paper.supp.tab.maas.mag[,c("MAG.ID","metadata","value","coef","stderr","N","N.not.0","pval","qval")],
            "tables/Supp_table_metag_maaslin_freq_res_230109.csv",
            row.names = F, quote = F)
}
```

## MAG functional analysis

### Mycoplasmataceae

Identify genes of interest involved in colonisation and virulence in
*Mycoplasmataceae* genomes. Comparative pangenome database was
created with Anvio, using the 5 *Mycoplasma* MAGs from this
study, 3 reference *Mycoplasma* genomes and 2 *Ureaplasma*
genomes as outgroups. Gene clusters were annotated with KEGG orthologues
and Pfam information.

Genes of interest: **adhesins** and related (mostly
identified from Yiwen et
al. 2021 review)

- *M. pneumoniae*:
  - **P1**
  - **OppA** (substrate-binding domain of the oligopeptide
    permease, important adhesin in human pathogenic mycoplasma)
  - **P30** (cytadherence-associated protein)
- *M. hyopneumoniae*:
  - **P97**
  - **MHJ\_0125** (a glutamyl aminopeptidase)
  - **MHJ\_0461** (a leucine aminopeptidase)
- *M. bovis*
  - ~~P27~~ (couldn’t find reliable sequence on NCBI
    Protein)
- *M. gallisepticum*:
  - **PlpA** - surface-exposed, fibronectin-binding protein
    important for virulence (homologue of M. pneumoniae
    cytadherence-associated protein P65)
- *M. conjunctivae*:
  - **LppT** - lipoprotein T involved in adhesion
- *M. hominis*
  - **Vaa** (variable adherence-associated antigen) aka P50
    (truncated)
  - **P60/P80**
- *M. hyorhinis*:
  - **Vlp** (variable lipoprotein) A-G
- *M. agalactiae* (Barbosa
  et al. 2022)
  - **MAG\_6130**
  - **P40**
- *M. mobile*:
  - **Gli349** (adhesion and gliding protein)

Genes of interest: putative **virulence factors**

Based on literature searches of known *Mycoplasma* pathogens
(mosty of mammals or birds)

- hydrogen peroxide production
  - linked to glycerol production
  - H2O2 tolerated by catalase & dismutase –> *some hits for
    both in Anvio annotations*
  - specifically:
    - **GlpF:** Glycerol uptake facilitator or related
      aquaporin
    - GlpK: Glycerol kinase –> *some hits in Anvio
      annotations*
    - gpsA: Glycerol-3-phosphate dehydrogenase –> *some hits for
      both in Anvio annotations*
    - **GlpO**: alpha-glycerophosphate oxidase /
      glycerol-3-phosphate oxidase, main generator of H2O2
- toxins: CARDS in *M. pneumoniae*
  - part of the pertussin, diphtheria and cholera superfamily of
    toxins
  - **CARDS**
- sialic acid catabolism - sialidase/neuraminidase activity
- other glycosidases
  - N-acetyl-β-hexosaminidase, α-mannosidase, hyaluronidase
  - beta-galactosidase - possibly role in bacterial adherence and
    deglycosylation of host extracellular matrix –> *some hits in
    Anvio annotations*
  - beta-glucosidase - unclear how, but is only known associated with
    virulence in M. mycoides –> *some hits in Anvio
    annotations*
  - alpha-amylase - possible roles in increased invasiveness, loss of
    extracellular matrix integrity, prolonged survivability by increasing
    nutritional fitness –> *some hits for domain in Anvio
    annotations*
  - chitinase - injure arthropod hosts while scavenging chitin from
    exoskeleton
- cytotoxic nucleases, e.g. P40 in *M. penetrans* –> *see
  adhesins list above*
- ammonia
  - by-product of ATP synthesis via arginine hydrolysis
  - carbamate kinase –> *some hits in Anvio annotations*
  - ornithine carbamoyltransferase –> *some hits in Anvio
    annotations*
  - arginine deiminase –> *some hits in Anvio
    annotations*
- Glycophorin A proteinase
  - interact with erythrocyte membranes containing glycophorin
- Gliding, occurs through different mechanisms (M. mobile vs M. pneumo
  etc) BUT doesn’t involve pili, flagellae, etc –> *see Gli in
  adhesins list above*

None of these adhesins and only some of the putative virulence
factors appear to be annotated in the Anvio pangenome (based on grep of
gene names) - used blast search instead. Used all amino acid sequences
of the pangenomics gene clusters from Anvio as the database, and
mycoplasma genes from the above literature search (in
**bold**) as query sequences, based on downloaded refs from
NCBI Protein.

```
# Anvio output
myco.genes <- read.delim("data/MetaG/Mycoplasmataceae_pangenomics_gene_clusters_summary.txt", sep = '\t')

# Anvio annotations
## no Mycoplasma adhesin genes annotated in Anvio, focos on virulence factors instead
## curated with exact terms to avoid multiple hits
myco.vf.terms <- c(
  "^glycerol kinase","^glycerol-3-phosphate dehydrogenase",
  "glycerol-3-phosphate dehydrogenase \\(",
  "NAD-dependent glycerol-3-phosphate dehydrogenase C-terminus",
  "NAD-dependent glycerol-3-phosphate dehydrogenase N-terminus",
  "^beta-galactosidase","beta-glucosidase","alpha amylase",
  "carbamate kinase","^ornithine carbamoyltransferase","arginine deiminase",
  "pertussis toxin","virulence","exfoliative toxin"
)

# now search for these terms
myco.vf.terms <- data.frame(
  gene=myco.vf.terms,
  KOfam=sapply(myco.vf.terms, function(x){
    h <- unique(grep(x,myco.genes$KOfam, value = T, ignore.case = T))
    if(length(h) == 1){ h } 
    else if(length(h) > 1) { paste(h, collapse = ";") }
    else { NA }
  }),
  Pfam=sapply(myco.vf.terms, function(x){
    h <- unique(grep(x,myco.genes$Pfam, value = T, ignore.case = T))
    if(length(h) == 1){ h } 
    else if(length(h) > 1) { paste(h, collapse = ";") }
    else { NA }
  }),
  gene.short=c("GK","gpsA","gpsA","gpsA","gpsA",
               "beta-gal","bgl","alpha-amyl","arcC","argF","arcA","pertussis","BrkB","ETs")
)

myco.vf.anvio <- rbind.data.frame(
  myco.genes[
    which(myco.genes$KOfam %in% myco.vf.terms$KOfam),
    c("KOfam","Pfam","genome_name")],
  myco.genes[
    which(myco.genes$Pfam %in% myco.vf.terms$Pfam),
    c("KOfam","Pfam","genome_name")]
)
myco.vf.anvio$gene.short <- sapply(seq(1,nrow(myco.vf.anvio),1), function(i){
  k <- myco.vf.anvio[i,"KOfam"]
  p <- myco.vf.anvio[i,"Pfam"]
  r <- c()
  if(k %in% myco.vf.terms$KOfam){
    r <- append(r,myco.vf.terms[which(myco.vf.terms$KOfam == k),"gene.short"])
  }
  if(p %in% myco.vf.terms$Pfam){
    r <- append(r,myco.vf.terms[which(myco.vf.terms$Pfam == p),"gene.short"])
  }
  if(length(r) == 0){
    NA
  } else {
    unique(r)
  }
})

myco.vf.anvio <- myco.vf.anvio[
  !duplicated(myco.vf.anvio[,c("genome_name","gene.short")]),
  c("genome_name","gene.short")]
names(myco.vf.anvio) <- c("subj.name","protein")
```

Blast search results

```
myco.adhesins.blast <- read.delim("data/MetaG/Mycoplasmataceae_pangenomics_blast_cytoadhesins.txt", sep="\t")
# exclude adhesins involved in essential cell processes: 
myco.adhesins.blast <- myco.adhesins.blast[which(!myco.adhesins.blast$protein %in% c("EF-Tu","PDHB")),]
myco.vfs.blast <- read.delim("data/MetaG/Mycoplasmataceae_pangenomics_blast_virulence.txt", sep='\t')

myco.blast.all <- rbind.data.frame(myco.adhesins.blast, myco.vfs.blast)

# for hits with at least 50% of query covered by at least one gene cluster of a MAG, display %ID range/mean in a heatmap. With the annotated ones too (in a different colour to show more certainty)

# calculate overlap (for some, alignment was too fragmented to calculate programmatically, it was faster to inspect overlaps manually)
myco.blast.overlap.manual <- read.delim("data/MetaG/Mycoplasmataceae_pangenomics_blast_alignment_percentage.txt")

myco.blast.all$cat.query.subj <- paste(myco.blast.all$query, myco.blast.all$subj.name, sep = ".")

myco.blast.summary <- data.frame(
  cat.query.subj=unique(myco.blast.all$cat.query.subj),
  aln.per=sapply(unique(myco.blast.all$cat.query.subj), function(x){
    df.sub <- myco.blast.all[which(myco.blast.all$cat.query.subj == x & myco.blast.all$e.value < 0.01),]
    q <- unique(df.sub$query)
    s <- unique(df.sub$subj.name)
    # percent alignment
    if(nrow(df.sub) == 0){
      # not significant so no alignment overlap calc necessary
      return(0)
    }  else if(nrow(df.sub) == 1){
      # just one hit, no fancy calcs needed
       return( (df.sub[1,"query.end"] - df.sub[1,"query.start"]) / df.sub[1,"length"] )
    } else if(nrow(df.sub) == 2) {
      # check for overlap
      df.sub <- df.sub[order(df.sub$query.start),]
      if(df.sub[1,"query.end"] < df.sub[2,"query.start"]){
        # no overlap, add alignments together
        a1 <- df.sub[1,"query.end"] - df.sub[1,"query.start"]
        a2 <- df.sub[2,"query.end"] - df.sub[2,"query.start"]
        return( (a1 + a2) / df.sub[1,"length"] )
      } else {
        # overlap, take whole interval
        return( (df.sub[2,"query.end"] - df.sub[1,"query.start"]) / df.sub[1,"length"] )
      }
      
    } else {
      return(myco.blast.overlap.manual[which(myco.blast.overlap.manual$cat.query.subj == x),"aln.per"])
    }
  }),
  pid.min=sapply(unique(myco.blast.all$cat.query.subj), function(x){
    # minimum PID for significant hits
    df.sub <- myco.blast.all[which(myco.blast.all$cat.query.subj == x & myco.blast.all$e.value < 0.01),]
    if(nrow(df.sub) == 0){
      # not significant so PID is 0
      return(0)
    }  else {
      return(min(df.sub$pid))
    }
  })
)

myco.blast.summary <- merge(myco.blast.summary, myco.blast.all[,c("query","protein","species","length","subj.name","cat.query.subj")],
                               by = "cat.query.subj", all.x = T, all.y = F)
myco.blast.summary$query.name <- paste(myco.blast.summary$species, myco.blast.summary$protein, sep = ".")
```

```
ggplot(myco.blast.summary[which(myco.blast.summary$aln.per > 0.5),], aes(protein, subj.name, fill = pid.min))+
  geom_tile()+
  scale_fill_gradient(low="white", high="blue", limits = c(0,100))+
  theme_bw()+
  theme(title = element_text(size = 16), 
        axis.text = element_text(size = 14, colour = "black"),
        axis.text.x = element_text(angle = 90),
        legend.text = element_text(size = 14, colour = "black"))
```

**Heatmap of blast search results** for genes with >
50% of bp aligned in each MAG or refererence genome. Gradient shows the
minimum percent identity recorded for an alignment. Genes included in
the blast search with no hits are not shown.

```
# combine Anvio and blast results
myco.vf.anvio$pid.min <- 100
myco.vf.anvio$aln.per <- 1

myco.pangenes.heatmap <- rbind.data.frame(
  myco.blast.summary[,c("subj.name","protein","pid.min","aln.per")],
  myco.vf.anvio
)

myco.pangenes.heatmap$subj.name <- factor(
  myco.pangenes.heatmap$subj.name,
  levels = c("REF_Ureaplasma_diversum","REF_Ureaplasma_urealyticum",
             "REF_Mycoplasma_penetrans","REF_Mycoplasma_iowae","MAG01_Mycoplasma_salmoninae",
             "MAG05_Mycoplasma_sp","MAG03_Mycoplasma_sp","REF_Mycoplasma_mobile",
             "MAG02_Mycoplasma_sp","MAG04_Mycoplasma_sp")
)
# ordered to match phylo tree

# table(myco.pangenes.heatmap$protein)
myco.pangenes.heatmap$protein <- factor(
  myco.pangenes.heatmap$protein,
  levels = c("arcC","argF","arcA", #             arginine metabolism
             "GlpF","GK","gpsA","GlpO", #        glycerol metabolism
             "MHJ_0125","MHJ_0461","PlpA", #     adhesins
             "P1","P30","P40","P50","P60","P80","P97",
             "MAG_6130","LppQ","LppT","OppA","Vaa","VlpA", 
             "alpha-amyl","beta-gal", #          adherence by cleaving ECM
             "bgl", #                            vf M. mycoides, mechanism unknown
             "Gli349", #                         vf M. mobile (gliding motion)
             "CARDS","pertussis","BrkB", #       toxins produced by M. pneumoniae / vf related to B. pertussis (BrkB)
             "ETs" #                             secreted exfoliative toxin related to Staph (not known in Mycoplasma?)
  )
)

myco.pangenes.heatmap$present <- ifelse(myco.pangenes.heatmap$aln.per > 0.5,1,0)
```

#### Figure 5A

```
ggplot(myco.pangenes.heatmap[which(myco.pangenes.heatmap$aln.per > 0.5),], aes(protein, subj.name, fill = present))+
  geom_tile()+
  scale_fill_gradient(low="white", high="grey70", limits = c(0,1))+
  geom_vline(xintercept = 3.5, size = 0.5, linetype = 1, color = "black")+
  geom_vline(xintercept = 7.5, size = 0.5, linetype = 1, color = "black")+
  geom_vline(xintercept = 14.5, size = 0.5, linetype = 1, color = "black")+
  geom_hline(yintercept = 2.5, size = 0.5, linetype = 2, color = "black")+
  geom_hline(yintercept = 4.5, size = 0.5, linetype = 2, color = "black")+
  geom_hline(yintercept = 5.5, size = 0.5, linetype = 2, color = "black")+
  geom_hline(yintercept = 8.5, size = 0.5, linetype = 2, color = "black")+
  theme_bw()+
  theme(axis.title = element_blank(), 
        axis.text.x = element_text(size = 12, colour = "black", angle = 90), 
        axis.text.y = element_text(size = 12, colour = "black"),
        # axis.text.x = element_blank(),
        # axis.text.y = element_blank(), axis.ticks.y = element_blank(),
        legend.position = "none",
        panel.grid = element_blank())
```

```
# write and edit in Inkscape, to annotate more nicely, fix MAG names, etc
# ggsave("figures/Figure_metag_myco_pangenomics_230314_nolabels.png", units = "in", dpi = 800, width = 5.5, height = 3.5)
```

**Fig. 5A**. Functional analysis of
*Mycoplasmataceae* MAGs compared to reference genomes. Genomes
are ordered by their phylogenetic relationships, from a tree built from
single-copy bacterial genes present in all genomes (tree not shown in
Rmarkdown). Trees were rooted using *Ureaplasma* reference
genomes as outgroups. Heatmap presents the presence (grey) or absence
(white) of genes involved in nutrient metabolism, colonisation and/or
virulence functions. Genes are grouped by related functions, including
genes in the arginine deiminase pathway, glycerol uptake and metabolism,
adherins that bind to host cell molecules and enzymes which cleave host
cell polysaccharides to aid adherence and more specific virulence
factors, including the *M. mobile* gliding mechanism Gli349 and
bacterial toxin production. A complete list of gene symbols with
detailed names and functions is provided in Table S12.

### Vibrionaceae

Possible virulence and colonisation factors:

- All vibrio have most of the required MSHA pili genes, which are
  thought to be involved in mutalistic interactions with host
  surfaces
- Most of the *Vibrio* spp. have a component of the pathogenic
  cholera “toxin” pili system, which is thought to be involved in *V.
  cholerae* colonisation of the gut. All *Aliivibrio* and
  *Photobacterium* spp. also have at least one gene from this
  system too, however - could assist with (pathogenic or commensal)
  colonisation of the gut?
- Not shown here, but majority of vibrio seem to have multiple
  pili/flagellin genes, so do appear to have the ability to be
  motile.
- *A. salmonicida* may have a disrupted chitin degradation
  pathway (point mutations) but some of the genes involved in chitin
  degradation are present in all *Aliivibrio* spp. Chitin usage
  might be associated with the bacteria’s ability to colonise marine
  invertebrates –> so we may or may not expect it to find in
  salmon-adapted bacteria.
- Most vibrio spp. have hemolysins and/or cytolysins present.
- The Lux operon has been implicated in virulence of *A.
  salmonicida* (as well as bioluminescence), and all
  *Aliivibrio* spp. seem to have most Lux genes.
  - structural luciferase operon:
    lux**C**DAB**E**GH
  - autoinducer sensers (to control light emission):
    **LuxN**, **LuxPQ**, AI-2
  - products that trigger autoinducers: LuxL, LuxM
  - this information is relayed by: **LuxU**
  - lux operon expresion modified (in response to autoinducers):
    **LuxO** (repressor), **LuxR**
    (transcriptional activator protein)
    - note, LuxR is also a family of related transcriptional activator
      proteins (including actual LuxR), that regulate other genes/operons
  - other quorum sensing regulators: **LuxS**,
    **LuxT**
- For iron-aquisition, all vibrio spp. have genes involved in iron
  uptake (fur), though there are few with other siderophobes (at least,
  which are named as such). *Aliivibrio* MAG09 and *A.
  salmonicida* MAG10 also have complete aerobactin pathways, which is
  also involved in iron acquisition from poor environments.
- All vibrio spp. have at least some genes involved in O-antigen
  biosynthesis, which may be requirement for *A. salmonicida*
  virulence in Atlantic salmon (https://pubmed.ncbi.nlm.nih.gov/30165113/).
  *Aliivibrio* spp. tend to have more complete LPS metabolism
  pathways as well.
- Other toxins are hard to evaluate, as also used by bacteria as
  defence against phage.
- pyocyanin produced by *Pseudomonas* has been suggested as a
  defense **AGAINST** pathogenic vibrios in aquaculture, yet
  many of my genomes/refs seem to have the ability to produce it? At least
  by one gene.

```
vibrio.genes <- read.delim("data/MetaG/Vibrionaceae_pangenomics_gene_clusters_summary.txt", sep = '\t')

# search KEGG modules for key terms:
# chitin-active polysaccharide monooxygenases
# toxin co-regulated pili
# MSHA pili
# hemolysins & cytolysins
# secretory proteases --> really broad...
# litR --> or HapR, can't find either
# lux operon
# siderophores e.g. bisucaberin
# fur family for iron aquisition
# spot 42 (RNA regulator) --> pirin-like protein
# O-antigenic oligosaccharide
# bile acid related

vibrio.vf.kegg <- unique(c(
  vibrio.genes[grep("pathogenicity",vibrio.genes$KEGG_Module, ignore.case = T),"KOfam_ACC"],
  vibrio.genes[grep("aerobactin",vibrio.genes$KEGG_Module, ignore.case = T),"KOfam_ACC"],
  vibrio.genes[grep("pyocyanine",vibrio.genes$KEGG_Module, ignore.case = T),"KOfam_ACC"],
  vibrio.genes[grep("D-glucuronate",vibrio.genes$KEGG_Module, ignore.case = T),"KOfam_ACC"],
  vibrio.genes[grep("Lipopolysaccharide metabolism",vibrio.genes$KEGG_Module, ignore.case = T),"KOfam_ACC"]
))

# search KO fam for key terms:
vibrio.vf.terms <- c("chitin","MSHA","hemolysin","cytolysin","Lux","siderophore","bisucaberin","catechol","Fur","spot 42","pirin","O-antigen","bile","mucin")

for(x in vibrio.vf.terms){
  vibrio.vf.kegg <- unique(append(
    vibrio.vf.kegg,vibrio.genes[grep(x,vibrio.genes$KOfam),"KOfam_ACC"]
  ))
}

vibrio.vf.pfam <- c()
for(x in vibrio.vf.terms){
  vibrio.vf.pfam <- unique(append(
    vibrio.vf.pfam,
    vibrio.genes[grep(x,vibrio.genes$Pfam),"Pfam_ACC"]
  ))
}

vibrio.vf.df <- rbind.data.frame(
  vibrio.genes[
    which(vibrio.genes$KOfam_ACC %in% vibrio.vf.kegg),
    c("KOfam","KOfam_ACC","Pfam","Pfam_ACC","KEGG_Module_ACC","KEGG_Class","genome_name")],
  vibrio.genes[
    which(vibrio.genes$Pfam_ACC %in% vibrio.vf.pfam),
    c("KOfam","KOfam_ACC","Pfam","Pfam_ACC","KEGG_Module_ACC","KEGG_Class","genome_name")]
)
vibrio.vf.df <- vibrio.vf.df[!duplicated(vibrio.vf.df),]

# annotation info on these genes
vibrio.vf.anno <- read.delim("data/MetaG/Vibrionaceae_pangenomics_virulence_gene_annotation.txt")

vibrio.vf.df$gene.short <- sapply(seq(1,nrow(vibrio.vf.df),1), function(i){
  k <- vibrio.vf.df[i,"KOfam_ACC"]
  p <- vibrio.vf.df[i,"Pfam_ACC"]
  if(k != ""){
    if(k %in% vibrio.vf.anno$KOfam_ACC){
      unique(vibrio.vf.anno[which(vibrio.vf.anno$KOfam_ACC == k),"gene.short"])
    } else {
      NA
    }
  } else {
    if(p %in% vibrio.vf.anno$Pfam_ACC){
      unique(vibrio.vf.anno[which(vibrio.vf.anno$Pfam_ACC == p),"gene.short"])
    }
    else {
      NA
    }
  }
})

vibrio.vf.df$Category <- sapply(seq(1,nrow(vibrio.vf.df),1), function(i){
  k <- vibrio.vf.df[i,"KOfam_ACC"]
  p <- vibrio.vf.df[i,"Pfam_ACC"]
  if(k != ""){
      unique(vibrio.vf.anno[which(vibrio.vf.anno$KOfam_ACC == k),"Category"])
  } else {
    unique(vibrio.vf.anno[which(vibrio.vf.anno$Pfam_ACC == p),"Category"])
  }
})

# for paper, cut LPS, since as gram neg bacteria, we'd expect this anyway.
# bile genes aren't well annotated in vibrio species (most google hits match to euks), so cutting as well
# pyocyanin gene phzE has homology to other enzymes and since only 1 gene from the pathway is there, cutting
# siderophore entS is only in 1 ref genome, cutting
# so many MSHA genes, doesn't add much, remove
# only include Lux operon genes (not LuxR regulators that have other roles

vibrio.exclude.genes <- c(
  unique(grep("^bile",vibrio.vf.df$gene.short, value = T)),
  unique(grep("^iron",vibrio.vf.df$gene.short, value = T)),
  unique(grep("^LPS",vibrio.vf.df$gene.short, value = T)),
  unique(grep("^pyocyan",vibrio.vf.df$gene.short, value = T)),
  unique(grep("^siderophore",vibrio.vf.df$gene.short, value = T)),
  unique(grep("^LuxR\\.",vibrio.vf.df$gene.short, value = T)),
  unique(grep("^MSHA",vibrio.vf.df$gene.short, value = T))
)

vibrio.pangenes.heatmap <- vibrio.vf.df[
  which(!vibrio.vf.df$gene.short %in% vibrio.exclude.genes),
  c("genome_name","gene.short","Category")]

# don't care whether there are duplicate copies of genes
vibrio.pangenes.heatmap <- vibrio.pangenes.heatmap[!duplicated(vibrio.pangenes.heatmap),]
vibrio.pangenes.heatmap$present <- 1

vibrio.pangenes.heatmap$subj.name <- factor(
  vibrio.pangenes.heatmap$genome_name,
  levels = c("REF_V_anguillarum","REF_V_splendidus","REF_V_cholerae",
             "MAG10_Aliivibrio_salmonicida","REF_A_salmonicida","REF_A_wodanis",
             "MAG08_Aliivibrio_sp","MAG09_Aliivibrio_sp","REF_A_fischeri",
             "REF_P_phosphoreum","MAG06_Photobacterium_phosphoreum","MAG07_Photobacterium_iliopiscarium",
             "REF_P_iliopiscarium","REF_P_profundum","REF_P_damselae")
)
# ordered to match phylo tree

vibrio.pangenes.heatmap$gene.short <- factor(
  vibrio.pangenes.heatmap$gene.short,
  levels = c("chia","chitin.AraC","chitin.cbp","chitin.chbG",
             "mucin.dgaF","mucin.eda","mucin.kdgK","mucin.uxaC","mucin.uxuA","mucin.uxuB",
             "aerobactin.iucC","aerobactin.iucA","aerobactin.iucB","aerobactin.iucD",
             "LuxC","LuxE","LuxN","LuxP","LuxQ","LuxU","LuxO","LuxR","LuxS","LuxT",
             "hemolysin.hlyIII","hemolysin.MarR","hemolysin.shlA","hemolysin.shlB","hemolysin.tlyC","hemolysin.tsh",
             "path.cholera.hemolysin","path.cholera.RTX","path.cholera.RtxA",
             "path.cholera.tcpI","path.cholera.tcpP","path.cholera.tdh",
             "path.cholera.tlh","path.cholera.Zot","path.T1SS","path.T4SS")
)
```

#### Figure 5B

```
ggplot(vibrio.pangenes.heatmap, aes(gene.short, subj.name, fill = present))+
  geom_tile()+
  scale_fill_gradient(low="white", high="grey70", na.value = "white", limits = c(0,1))+
  geom_vline(xintercept = 4.5, size = 0.5, linetype = 1, color = "black")+
  geom_vline(xintercept = 10.5, size = 0.5, linetype = 1, color = "black")+
  geom_vline(xintercept = 14.5, size = 0.5, linetype = 1, color = "black")+
  geom_vline(xintercept = 24.5, size = 0.5, linetype = 1, color = "black")+
  geom_vline(xintercept = 30.5, size = 0.5, linetype = 1, color = "black")+
  geom_hline(yintercept = 3.5, size = 0.5, linetype = 2, color = "black")+
  geom_hline(yintercept = 9.5, size = 0.5, linetype = 2, color = "black")+
  theme_bw()+
  theme(axis.title = element_blank(),
        axis.text.x = element_text(size = 12, colour = "black", angle = 90),
        # axis.text.x = element_blank(),
        # axis.text.y = element_blank(), axis.ticks.y = element_blank(),
        legend.position = "none",
        panel.grid = element_blank())
```

```
# write and edit in Inkscape, to annotate more nicely, etc
# ggsave("figures/Figure_metag_vibrio_pangenomics_230822_nolabels.png", units = "in", dpi = 800, width = 5.5, height = 4)
```

**Fig. 5B**. Functional analysis of
*Vibrionaceae* MAGs compared to reference genomes. Genomes are
ordered by their phylogenetic relationships, from a tree built from
single-copy bacterial genes present in all genomes (tree not shown in
Rmarkdown). Trees were rooted using *Vibrio* reference genomes as
outgroups. Heatmap presents the presence (grey) or absence (white) of
genes involved in nutrient metabolism, colonisation and/or virulence
functions. Genes are grouped by related functions, including genes of
known relevance to the *Vibrionaceae* clade, specifically genes
involved in chitin degradation, (involved in the colonisation of
invertebrates), the D-glucuronate degradation pathway (involved in
adaptation to the gut), synthesis of aerobactin (siderophore used to
sequester iron), community communication via quorum sensing, hemolysin
toxin production and other toxin-producing genes. A complete list of
gene symbols with detailed names and functions is provided in Table
S12.

## HostG

General processing of HostG data:

- preprocessing (adapter removal, mapping to reference genome,
  duplicate removal, etc)
- genotype likelihoods estimated with ANGSD
- genotype probabilities estimated with Beagle (SNP input for
  GWAS)
- linkage disequilibrium pruning
- population structure with PCAngsd (covariates for GWAS)

### PCA of population structure

```
# order of samples in angsd, gemma etc output
hostg.sample.order <- read.delim("data/HostG/HostG_sample_order_GWAS.txt", header = F)
#PCAngsd results
hostg.pca <- read.csv("data/HostG/HostG_pcangsd_results.csv")

hostg.e <- data.frame(eigenvalue=hostg.pca$eigenvalues, PVE=hostg.pca$eigenvalues/sum(hostg.pca$eigenvalues))
hostg.pc1 <- round(hostg.e$PVE,3)[1]*100
hostg.pc2 <- round(hostg.e$PVE,3)[2]*100
hostg.pc3 <- round(hostg.e$PVE,3)[3]*100
hostg.pc4 <- round(hostg.e$PVE,3)[4]*100
hostg.pc4 <- round(hostg.e$PVE,3)[5]*100

hostg.pca <- merge(hostg.pca[,c("Fish.ID","V1","V2","V3","V4","V5")], meta[,c("Fish.ID",metavar.fish,metavar.exp)],
                  by = "Fish.ID", all.x = T, all.y = F)

hp1 <- 
ggplot(hostg.pca, aes(V1, V2, color=Feed.Type, shape=Feed.Type))+
  geom_vline(xintercept = 0, linetype = 3, size = 1, color = "grey42")+
  geom_hline(yintercept = 0, linetype = 3, size = 1, color = "grey42")+
  geom_point(size = 3)+
  scale_color_manual(values = anno_colours$Feed.Type)+
  labs(x = paste0("PC1 (",hostg.pc1,"%)"), y = paste0("PC2 (",hostg.pc2,"%)"), title = "Feed type")+
  theme_bw()+
  theme(axis.text = element_text(color = "black"))

hp2 <-
ggplot(hostg.pca, aes(V1, V2, shape=Feed.Type, color=Size.class))+
  geom_vline(xintercept = 0, linetype = 3, size = 1, color = "grey42")+
  geom_hline(yintercept = 0, linetype = 3, size = 1, color = "grey42")+
  geom_point(size = 3)+
  scale_color_manual(values = anno_colours$Size.class)+
  labs(x = paste0("PC1 (",hostg.pc1,"%)"), y = paste0("PC2 (",hostg.pc2,"%)"), title = "Size class")+
  theme_bw()+
  theme(axis.text = element_text(color = "black"))

hp3 <-
ggplot(hostg.pca, aes(V1, V2, shape=Size.class, color=Tapeworm))+
  geom_vline(xintercept = 0, linetype = 3, size = 1, color = "grey42")+
  geom_hline(yintercept = 0, linetype = 3, size = 1, color = "grey42")+
  geom_point(size = 3)+
  scale_color_manual(values = anno_colours$Tapeworm)+
  labs(x = paste0("PC1 (",hostg.pc1,"%)"), y = paste0("PC2 (",hostg.pc2,"%)"), title = "Cestode index")+
  theme_bw()+
  theme(axis.text = element_text(color = "black"))

cowplot::plot_grid(hp1, hp2, hp3, align = "hv", axis = "tblr", nrow = 1)
```

**PCA of salmon population structure** coloured by feed
type, size class and tapeworm index. Individuals are highly related.
Subpopulation clusters likely correlate with salmon paternal lines used
for broodstock, rather than the fish phenotypes, although since we don’t
have parental data, we can’t test this hypothesis here.

**Prepare metadata for GWAS runs**

```
bins.included.pa <- names(bin.PA[,-1])[which(colSums(bin.PA[,-1]) > 10 & colSums(bin.PA[,-1]) < (nrow(bin.PA) - 10))]
bins.included.ab <- names(bin.PA[,-1])[which(colSums(bin.PA[,-1]) > 50)]
```

For microbiome data, excluded bins detected (or not detected, in the
case of *M. salmoninae*) in < 10 samples for presence/absence
(resulting in 11 bins included) and those detected in < 50 samples
for abundance (resulting in 8 bins included). Sample order *must*
match order of samples in genotype probabilities file.

- Run 0: fish phenotypes
  - gutted weight (transformed to approximate normal using INT)
  - cestode detection (0,1)
  - feed type (0,1)
- Run 1: MAG abundance (transformed to approximate normal using
  INT)
- Run 2: MAG detection (0,1)
- Run 3: microbiome phenotypes
  - as defined in MetaG community analysis section
  - plus alpha diversity (INT)
  - plus NMDS 1 & 2 from MAG abundance ordination

```
## phenotype files
# run1: abundance
process_pheno_data_for_gwas_f(bin.filt[,bins.included.ab], bin.filt$Fish.ID, "data/HostG/HostG_sample_order_GWAS.txt", "data/HostG/GWAS_gemma_phenoData_MAG_INT.txt", abund_thresh = 0, qtrans = T)

# run2: presence/absence
temp.bin.pa <- bin.PA[,-1]
temp.bin.pa[temp.bin.pa == TRUE] <- 1
process_pheno_data_for_gwas_f(temp.bin.pa[,bins.included.pa], bin.PA$Fish.ID, "data/HostG/HostG_sample_order_GWAS.txt", "data/HostG/GWAS_gemma_phenoData_MAG_PA.txt", abund_thresh = -1, qtrans = F)
rm(temp.bin.pa)

# run3: microbiome phenotypes
temp.micro.pheno <- data.frame(
  Alpha.INT=qtrans(meta$Bin.alpha.hill1),
  NMDS1=meta$Bin.ab.NMDS1,
  NMDS2=meta$Bin.ab.NMDS2,
  Photo.PA=ifelse(meta$Bin.pa.Photo,1,0),
  Ali.PA=ifelse(meta$Bin.pa.Ali,1,0),
  Myco.PA=ifelse(meta$Bin.pa.Myco,1,0),
  Msal.high=ifelse(meta$Bin.pa.Msal.high,1,0)
)
row.names(temp.micro.pheno) <- meta$Fish.ID
temp.micro.pheno <- temp.micro.pheno[!is.na(temp.micro.pheno$Alpha.INT),]
process_pheno_data_for_gwas_f(temp.micro.pheno, row.names(temp.micro.pheno), "data/HostG/HostG_sample_order_GWAS.txt", "data/HostG/GWAS_gemma_phenoData_microbiome_vars.txt", abund_thresh = -1000, qtrans = F)
```

```
## covariate file:
# PC1-5
# gutted weight
# feed type
## will also use for run0 ("single association") of feed type, size and tapeworm

hostg.pca$Weight.INT <- qtrans(hostg.pca$Gutted.Weight.kg)
hostg.pca$Feed.Type <- ifelse(hostg.pca$Feed.Type == "Feed1",0,1)
hostg.pca$Tapeworm <- ifelse(hostg.pca$Tapeworm.bin,1,0)

process_cov_data_for_gwas_f(hostg.pca, hostg.pca$Fish.ID, "data/HostG/HostG_sample_order_GWAS.txt", "data/HostG/GWAS_gemma_covData.txt")
```

### GWAS results

MAG05 results on chr 5 region is the main association of
interest.

```
MAG05.chr5.res <- read.delim("output_GWAS/binPA_MAG05_Mycoplasma_sp_chr5.assoc.txt")
MAG05.chr5.snplist <- scan("output_GWAS/binPA_MAG05_Mycoplasma_sp_sigSNPs_chr5_list.txt", what = character())
MAG05.chr5.anno <- read.csv("output_GWAS/GWAS_SNP_annotation_chr5.csv")
MAG05.chr5.geno <- read.delim("output_GWAS/GWAS_SNP_genotype_probabilities_chr5.gprobs", header = T, sep = " ")

# "genotype" samples based on genotype probabilities
names(MAG05.chr5.geno)[1:3] <- c("marker","alleleA","alleleB")
names(MAG05.chr5.geno)[4:ncol(MAG05.chr5.geno)] <- rep(hostg.sample.order$V1, times = rep(3,length(hostg.sample.order$V1)))
names(MAG05.chr5.geno)[4:ncol(MAG05.chr5.geno)] <- sapply(seq(4,ncol(MAG05.chr5.geno)), function(x){
  if(x %% 3 == 1){
    # first entry = major/major (MM)
    paste(names(MAG05.chr5.geno)[x],".MM", sep = "")
  } else if(x %% 3 == 2){
    # second entry = major/minor (Mm)
    paste(names(MAG05.chr5.geno)[x],".Mm", sep = "")
  } else {
    # third entry = minor/minor (mm)
    paste(names(MAG05.chr5.geno)[x],".mm", sep = "")
  }
})

MAG05.chr5.geno.long <- melt(MAG05.chr5.geno, id.vars = c("marker","alleleA","alleleB"), 
                             measure.vars = c(4:ncol(MAG05.chr5.geno)),
                             variable.name = "Sample.allele", value.name = "gprob")
MAG05.chr5.geno.long$Fish.ID <- sapply(MAG05.chr5.geno.long$Sample.allele, function(x){
  strsplit(as.character(x), split = "\\.")[[1]][1]
})
MAG05.chr5.geno.long$allele <- sapply(MAG05.chr5.geno.long$Sample.allele, function(x){
  strsplit(as.character(x), split = "\\.")[[1]][2]
})
MAG05.chr5.geno.long$SNP.in.GWAS <- MAG05.chr5.geno.long$marker %in% MAG05.chr5.snplist

# call based on probability > 0.8
MAG05.chr5.geno.long$genotype.call <- MAG05.chr5.geno.long$gprob > 0.8

MAG05.chr5.geno.call <- dcast(subset(MAG05.chr5.geno.long, genotype.call), marker ~ Fish.ID, value.var = "allele")

# get frequency of SNP calls by MAG05 detection
MAG05.chr5.geno.freq <- data.frame(
  marker=MAG05.chr5.geno.call$marker,
  MAG05.pos.M=sapply(seq(1,nrow(MAG05.chr5.geno.call)), function(i){
    samples <- meta[which(meta$Bin.pa.MAG05 & meta$Fish.ID %in% hostg.samples),"Fish.ID"]
    MM <- length(grep("MM",MAG05.chr5.geno.call[i,samples]))
    Mm <- length(grep("Mm",MAG05.chr5.geno.call[i,samples]))
    (MM * 2 + Mm) / (2 * ncol(MAG05.chr5.geno.call[,samples]))
  }),
  MAG05.neg.M=sapply(seq(1,nrow(MAG05.chr5.geno.call)), function(i){
    samples <- meta[which(!meta$Bin.pa.MAG05 & meta$Fish.ID %in% hostg.samples),"Fish.ID"]
    MM <- length(grep("MM",MAG05.chr5.geno.call[i,samples]))
    Mm <- length(grep("Mm",MAG05.chr5.geno.call[i,samples]))
    (MM * 2 + Mm) / (2 * ncol(MAG05.chr5.geno.call[,samples]))
  }),
  MAG05.pos.m=sapply(seq(1,nrow(MAG05.chr5.geno.call)), function(i){
    samples <- meta[which(meta$Bin.pa.MAG05 & meta$Fish.ID %in% hostg.samples),"Fish.ID"]
    mm <- length(grep("mm",MAG05.chr5.geno.call[i,samples]))
    Mm <- length(grep("Mm",MAG05.chr5.geno.call[i,samples]))
    (mm * 2 + Mm) / (2 * ncol(MAG05.chr5.geno.call[,samples]))
  }),
  MAG05.neg.m=sapply(seq(1,nrow(MAG05.chr5.geno.call)), function(i){
    samples <- meta[which(!meta$Bin.pa.MAG05 & meta$Fish.ID %in% hostg.samples),"Fish.ID"]
    mm <- length(grep("mm",MAG05.chr5.geno.call[i,samples]))
    Mm <- length(grep("Mm",MAG05.chr5.geno.call[i,samples]))
    (mm * 2 + Mm) / (2 * ncol(MAG05.chr5.geno.call[,samples]))
  })
)

# save genes that have SNPs in RNA dataset
MAG05.chr5.rna.genes <- MAG05.chr5.anno[which(!MAG05.chr5.anno$RNA.gene %in% c("no.RNA.data","no.annotation")),c("rs","RNA.gene")]
```

#### Figure 3A

Manhatten plot

```
# Manhattan upper limit
# -log10(min(MAG05.chr5.res$p_wald))
# 8.791007

MAG05.chr5.res$p_trans <- -log10(MAG05.chr5.res$p_wald)
MAG05.chr5.res$highlight_snp <- MAG05.chr5.res$rs %in% MAG05.chr5.snplist
MAG05.chr5.res$annotated_gene <- MAG05.chr5.res$rs %in% MAG05.chr5.anno[which(MAG05.chr5.anno$RNA.gene != "no.annotation"),"rs"]

MAG05.chr5.res$annotation2 <- sapply(MAG05.chr5.res$rs, function(x){
  if(x %in% c("NC_059446.1_15487010","NC_059446.1_15487786")){
    "ANKHD1"
  } else if(x %in% c("NC_059446.1_16091245",
                     "NC_059446.1_16091912","NC_059446.1_16092937",
                     "NC_059446.1_16099402","NC_059446.1_16099761",
                     "NC_059446.1_16106363","NC_059446.1_16107109")){
    "TENM2"
  } else if(x %in% c("NC_059446.1_17334765","NC_059446.1_17343411","NC_059446.1_17347221",
                     "NC_059446.1_17347254","NC_059446.1_17359592")){
    "GRIA1A"
  } else if(x %in% c("NC_059446.1_16256914","NC_059446.1_16274373",
                       "NC_059446.1_16363100","NC_059446.1_16363213")){
    "lncRNA"
  } else if(x %in% MAG05.chr5.geno.freq$marker){
    "intergenic"
  } else {
    ""
  }
})

fig3a <-
ggplot(MAG05.chr5.res, aes(ps, p_trans, color = annotation2, shape = highlight_snp))+
  geom_hline(yintercept = -log10(1e-5), color = "blue")+
  geom_hline(yintercept = -log10(5e-8), color = "red")+
  geom_point(size = 2)+
  scale_y_continuous(limits = c(0,9))+
  scale_color_manual(values = c("grey70","#F781BF","#984EA3","black","gold","#4DAF4A"))+
  scale_shape_manual(values = c(16,17))+
  labs(x = "Position on chr 5",
       y = expression(paste("-log"[10], plain(P))))+
  theme_classic()+
  theme(axis.text = element_text(color = "black", size = 12), legend.position = "none",
        axis.title = element_text(size = 13), 
        plot.title = element_text(size = 13, color = "black", hjust = 0.5, face = "bold"))

fig3a
```

**Fig. 3A**. MAG05 Mycoplasma GWAS results.
(**A**) SNP peak observed on chromosome 5 for the GWAS
association with MAG05 presence/absence. P values have been -log10
transformed so that higher values are more significant. Moderately (p
< 1e-5, blue line) and highly (p < 5e-8, red line) associated SNPs
on the peak are highlighted in triangles against the background grey
circles of chromosome 5. SNPs falling within annotated transcripts in
this peak are coloured and annotated by their gene symbol, where
relevant (ANKHD1 (pink): ankyrin repeat and KH domain-containing protein
1-like; TENM2 (green): teneurin-2-like; GRM1 (purple): glutamate
receptor 1-like; lncRNAs (yellow): long noncoding RNAs). SNPs falling
within intergenic regions are shown as black triangles.

#### Figure 3B

qqplot

```
## note, qqplot takes a long time to load
gg_qqplot_df <- function(ps, ci = 0.95) {
  n  <- length(ps)
  df <- data.frame(
    observed = -log10(sort(ps)),
    expected = -log10(ppoints(n)),
    clower   = -log10(qbeta(p = (1 - ci) / 2, shape1 = 1:n, shape2 = n:1)),
    cupper   = -log10(qbeta(p = (1 + ci) / 2, shape1 = 1:n, shape2 = n:1))
  )
  return(df)
}

gwas.mag05.res.all <- read.delim(gzfile("output_GWAS/binPA_MAG05_Mycoplasma_sp_all.assoc.clean.txt.gz","rt"))
MAG05.qqplot.df <- gg_qqplot_df(gwas.mag05.res.all$p_wald)
# gwas.mag05.res.all is large, remove after getting plot data
rm(gwas.mag05.res.all)

fig3b <-
ggplot(MAG05.qqplot.df)+
  geom_hline(yintercept = -log10(1e-5), color = "blue")+
  geom_hline(yintercept = -log10(5e-8), color = "red")+
  geom_ribbon(
    mapping = aes(x = expected, ymin = clower, ymax = cupper),
    alpha = 0.1
  )+
  geom_point(aes(expected, observed), shape = 16, size = 2)+
  geom_abline(intercept = 0, slope = 1, alpha = 0.5)+
  labs(x = expression(paste("Expected -log"[10], plain(P))),
       y = expression(paste("-log"[10], plain(P))))+
  theme_classic()+
  theme(axis.text = element_text(color = "black", size = 12), legend.position = "none",
        axis.title = element_text(size = 13),
        plot.title = element_text(size = 13, color = "black", hjust = 0.5, face = "bold"))

# ggsave("figures/Figure_gwas_inflation_MAG05chr5.png", units = "in", dpi = 800, width = 5, height = 5)

fig3b
```

**Fig. 3B.** MAG05 Mycoplasma GWAS results.
(**B**) QQ plot and genomic inflation factor (λ) for the
GWAS association with MAG05 presence/absence.

#### Figure 3C

Heatmap of allele frequencies on chr5

```
row.names(MAG05.chr5.geno.freq) <- MAG05.chr5.geno.freq$marker

# SNP annotation for pheatmap
MAG05.chr5.hm.anno <- data.frame(
  location=sapply(MAG05.chr5.geno.freq$marker, function(x){
    if(x %in% c("NC_059446.1_15487010","NC_059446.1_17343411")){
      "CDS.syn"
    } else if(x %in% c("NC_059446.1_15487786","NC_059446.1_16091245",
                       "NC_059446.1_16091912","NC_059446.1_16092937",
                       "NC_059446.1_16099402","NC_059446.1_16099761",
                       "NC_059446.1_16106363","NC_059446.1_16107109",
                       "NC_059446.1_17334765","NC_059446.1_17347221",
                       "NC_059446.1_17347254","NC_059446.1_17359592")){
      "intron"
    } else if(x %in% c("NC_059446.1_16256914","NC_059446.1_16274373",
                       "NC_059446.1_16363100","NC_059446.1_16363213")){
      "lncRNA"
    } else {
      "intergenic"
    }
  }),
  gene=sapply(MAG05.chr5.geno.freq$marker, function(x){
    if(x %in% c("NC_059446.1_15487010","NC_059446.1_15487786")){
      "ANKHD1"
    } else if(x %in% c("NC_059446.1_16091245",
                       "NC_059446.1_16091912","NC_059446.1_16092937",
                       "NC_059446.1_16099402","NC_059446.1_16099761",
                       "NC_059446.1_16106363","NC_059446.1_16107109")){
      "TENM2"
    } else if(x %in% c("NC_059446.1_17334765","NC_059446.1_17343411","NC_059446.1_17347221",
                       "NC_059446.1_17347254","NC_059446.1_17359592")){
      "GRIA1A"
    } else {
      NA
    }
  })
)
row.names(MAG05.chr5.hm.anno) <- MAG05.chr5.geno.freq$marker
MAG05.chr5.hm.samples.anno <- data.frame(
  MAG05.det=c("Yes","No","Yes","No"),
  allele=c("M","M","m","m")
)
row.names(MAG05.chr5.hm.samples.anno) <- names(MAG05.chr5.geno.freq[,-1])

MAG05.chr5.hm.colors <- list(
  MAG05.det=c(Yes="#804ec2", No="#cfb0f7"),
  allele=c(M="grey50", m="grey80"),
  location=c(CDS.syn="#E41A1C", intron="#62d9d5", lncRNA="gold", intergenic="black"),
  gene=c(ANKHD1="#F781BF",TENM2="#4DAF4A",GRIA1A="#984EA3")
)
```

```
# pheatmap cannot be included in cowplot
# png("figures/Figure_mGWAS_allele_freq_heatmap_230530.png", units = "in", res = 600, width = 5, height = 3.4)
# pheatmap call, include: annotation_legend = F, filename = "figures/Figure_mGWAS_allele_freq_heatmap.png 
# dev.off()

pheatmap(MAG05.chr5.geno.freq[,c(5,3,4,2)],
         cluster_rows = F, cluster_cols = F,
         breaks = seq(0, 1, by = 0.01),
         annotation_row = MAG05.chr5.hm.anno, 
         annotation_col = MAG05.chr5.hm.samples.anno, 
         annotation_colors = MAG05.chr5.hm.colors,
         show_colnames = F, legend = T)
```

**Fig. 3C**. MAG05 Mycoplasma GWAS results.
(**C**) Minor and major allele frequencies for the 28
associated (bold face) and linked (plain face) SNPs. Frequencies have
been calculated separately for individuals with MAG05 present or absent.
SNPs are annotated by gene symbol (where relevant, same as in
**A**) and genomic region (CDS: coding sequence).

## HostRNA

```
# Get Salmon annotation info
# get OrgDb from AnnotationHub
ah <- AnnotationHub(cache = "C:/Users/jaelleb/R/AnnotationHub")
```

```
## snapshotDate(): 2022-04-25
```

```
# list OrgDb's available for Salmo salar
subset(ah, ah$species=="Salmo salar" & ah$rdataclass=="OrgDb")
```

```
## AnnotationHub with 1 record
## # snapshotDate(): 2022-04-25
## # names(): AH100820
## # $dataprovider: ftp://ftp.ncbi.nlm.nih.gov/gene/DATA/
## # $species: Salmo salar
## # $rdataclass: OrgDb
## # $rdatadateadded: 2022-04-21
## # $title: org.Salmo_salar.eg.sqlite
## # $description: NCBI gene ID based annotations about Salmo salar
## # $taxonomyid: 8030
## # $genome: NCBI genomes
## # $sourcetype: NCBI/UniProt
## # $sourceurl: ftp://ftp.ncbi.nlm.nih.gov/gene/DATA/, ftp://ftp.uniprot.org/p...
## # $sourcesize: NA
## # $tags: c("NCBI", "Gene", "Annotation") 
## # retrieve record with 'object[["AH100820"]]'
```

```
# retrieve the Ssal OrgDb
SsalOrg <- ah[["AH100820"]]
```

```
## loading from cache
```

Processing steps:

- STAR processing produced raw gene counts table
- Only retained samples with > 50% reads aligned
- Filtering out genes with < 50% of samples having a count of >=
  10
- Normalisation with DESeq:
  - Simple model design (feed type + size class)
  - Library size factors controlled by shorth function
    (normalisation)
  - VST transformation by DESeq2

```
# load and process RNA data
rna.mapping.stats <- read.csv("data/HostRNA/RNA_mapping_stats.csv")
# can't upload adjusted_read_counts_innerjoin.csv to github (file too big)
# needs to be downloaded from [PENDING]
rna.gene.cts <- read.csv("../../RCdr_HostRNA/adjusted_read_counts_innerjoin_FishID.csv")

# Filter samples at 50% reads mapped
rna.samples.50 <- rna.mapping.stats[which(rna.mapping.stats$Mapped.Percent > 50),"Fish.ID"]
print(paste(length(rna.samples.50), "samples remain after filtering at 50%."))
```

```
## [1] "343 samples remain after filtering at 50%."
```

```
rna.gene.cts <- rna.gene.cts[,c("chr.gene",rna.samples.50)]

# Save as csv
# write.csv(rna.gene.cts,"data/HostRNA/RNA_adjusted_read_counts_innerjoin_filtered50percent.csv", row.names = F, quote = F)

# Filtering gene steps:
# remove genes with zero counts
# remove genes with < 50% of samples with >= 10 counts
row.names(rna.gene.cts) <- rna.gene.cts$chr.gene
rna.gene.cts <- rna.gene.cts[,-1]
rna.gene.cts <- rna.gene.cts[rowSums(rna.gene.cts) >  0,]
rna.gene.cts <- rna.gene.cts[rowSums(rna.gene.cts >= 10) >= ncol(rna.gene.cts)/2,]

# Change the count table to integers for DESeq2 analysis (note rounds down, not up)
rna.gene.cts[] <- lapply(rna.gene.cts, as.integer)
```

```
# DESeq for normalisation and transformation, using simple model for error correction
# Subset metadata
rna.meta <- meta[which(meta$Fish.ID %in% rna.samples),c("Fish.ID", metavar.fish, grep("Bin",names(meta), value = T))]
row.names(rna.meta) <- rna.meta$Fish.ID

# Set up DDS object - make sure metadata samples (rows) match gene count samples (columns)
# go with simple design for data exploration
rna.design.simple <- model.matrix(~ Feed.Type + Size.class, data = rna.meta[order(rna.meta$Fish.ID),])
rna.meta <- rna.meta[order(rna.meta$Fish.ID),]
rna.gene.cts <- rna.gene.cts[,order(names(rna.gene.cts))]

rna.dds <- DESeqDataSetFromMatrix(
  countData = rna.gene.cts, 
  colData = rna.meta, 
  design = rna.design.simple
)

rna.dds <- estimateSizeFactors(rna.dds, locfunc = genefilter::shorth)

# transformed data for exploration
# Using VST transformation - blind = FALSE (did test blind = TRUE and it didn't make a big difference)
# note that VST transforms the data into log2 space (among other things), so can use tools like limma with it
rna.vst <- vst(rna.dds, blind = FALSE)
rna.vst.data <- assay(rna.vst)
```

### Ordination

```
rna.pca <- prcomp(t(rna.vst.data))

# get coordinates
rna.pca.coord <- as.data.frame(get_pca_ind(rna.pca)$coord)
rna.pca.coord$Fish.ID <- row.names(rna.pca.coord)
rna.pca.coord <- merge(rna.pca.coord[,c("Fish.ID","Dim.1","Dim.2","Dim.3","Dim.4")], rna.meta, by = "Fish.ID", all.x = TRUE)

# get % variance
rna.pca.eig <- get_eigenvalue(rna.pca)[c("Dim.1","Dim.2","Dim.3","Dim.4"),]

# get gene contributions
rna.pca.contrib <- get_pca_var(rna.pca)$contrib[,c("Dim.1","Dim.2","Dim.3","Dim.4")]
```

#### Suplementary figure

Observations: Some of the variation seems to be explained by feed
type and size class, but no strong trends.

```
supp.fig.pca1 <-
ggplot(rna.pca.coord, aes(Dim.1, Dim.3, color=Feed.Type))+
  geom_point(size=3)+
  scale_color_manual(values = anno_colours$Feed.Type)+
  labs(x = paste0("PC1: ",round(rna.pca.eig[1,2],1),"% variance"),
       y = paste0("PC3: ",round(rna.pca.eig[3,2],1),"% variance"),
       color = "Feed type")+
  theme_classic()+
  theme(axis.text = element_text(color = "black", size = 12), axis.title = element_text(size = 13),
        legend.position = "right", legend.text = element_text(size = 12), legend.title = element_text(size = 12))

supp.fig.pca2 <-
ggplot(rna.pca.coord, aes(Dim.1, Dim.4, color=Size.class))+
  geom_point(size=3)+
  scale_color_manual(values = anno_colours$Size.class)+
  labs(x = paste0("PC1: ",round(rna.pca.eig[1,2],1),"% variance"),
       y = paste0("PC4: ",round(rna.pca.eig[4,2],1),"% variance"),
       color = "Size class")+
  theme_classic()+
  theme(axis.text = element_text(color = "black", size = 12), axis.title = element_text(size = 13),
        legend.position = "right", legend.text = element_text(size = 12), legend.title = element_text(size = 12))

cowplot::plot_grid(supp.fig.pca1, supp.fig.pca2, align = "hv", axis = "tblr", nrow = 2, labels = c("A","B"))
```

```
# ggsave("figures/Supp_figure_hostRNA_PCAs.png", units = "in", dpi = 800, width = 6, height = 7)
```

**Fig. S2.** Ordination (PCA) of host gut epithelial
transcriptomes. Individuals showed some variation by feed type on PC3
(**A**) and some variation by size class on PC4
(**B**). PCAs were based on normalised and transformed gene
expression data. Based on PERMANOVA results using Euclidean distances,
feed type contributed to 2.7% of the variation in gene expression values
(F = 9.63, p = 0.001) and size class contributed to 2.0% of the
variation (F = 3.53, p = 0.001). Detailed results from the PERMANOVAs
are presented in Table S1.

```
rna.dist <- vegdist(t(rna.vst.data), method = "euclidean")

# adonis2(rna.dist ~ Feed.Type + Size.class, rna.meta,
#         permutations = 999, method = "euclidean")

perm.rna <- adonis2(rna.dist ~ Feed.Type + Size.class + Tapeworm, rna.meta,
                    permutations = 999, method = "euclidean")
perm.rna
```

```
## Permutation test for adonis under reduced model
## Terms added sequentially (first to last)
## Permutation: free
## Number of permutations: 999
## 
## adonis2(formula = rna.dist ~ Feed.Type + Size.class + Tapeworm, data = rna.meta, permutations = 999, method = "euclidean")
##             Df SumOfSqs      R2      F Pr(>F)    
## Feed.Type    1    84924 0.02709 9.6260  0.001 ***
## Size.class   2    62210 0.01984 3.5257  0.001 ***
## Tapeworm     3    23764 0.00758 0.8979  0.655    
## Residual   336  2964301 0.94549                  
## Total      342  3135199 1.00000                  
## ---
## Signif. codes:  0 '***' 0.001 '**' 0.01 '*' 0.05 '.' 0.1 ' ' 1
```

```
# format to print as table
perm.rna.df <- as.data.frame(perm.rna)
perm.rna.df$Variable <- row.names(perm.rna.df)
perm.rna.df$Dataset <- "HostRNA"
row.names(perm.rna.df) <- NULL
```

### Figure 3D

Only 1 gene from GWAS is present in RNA dataset:
NC\_059446.1+LOC106604312. Plot gene expression of this gene by MAG05
detection and SNP genotype

```
fig3d.df <- meta[which(!is.na(meta$Bin.pa.MAG05) & meta$HostTranscriptome),c("Fish.ID","Bin.pa.MAG05")]
fig3d.df$HostG.LOC106604312 <- sapply(fig3d.df$Fish.ID, function(x){
  if(x %in% names(MAG05.chr5.geno.call)){
    MAG05.chr5.geno.call[which(MAG05.chr5.geno.call$marker == MAG05.chr5.rna.genes$rs),x]
  } else {
    NA
  }
})

fig3d.df <- get_feature_abundance_meta( 
  abund.mat = as.matrix(rna.vst.data),  
  feature.ids = MAG05.chr5.rna.genes$RNA.gene,
  meta = fig3d.df,
  sample.var = "Fish.ID",
  sample.ids = fig3d.df[which(!is.na(fig3d.df$HostG.LOC106604312)),"Fish.ID"]
)

fig3d.df$SNP.MAG05det <- interaction(fig3d.df$HostG.LOC106604312, as.character(fig3d.df$Bin.pa.MAG05))
```

```
ggplot(fig3d.df, aes(SNP.MAG05det, abundance))+
  geom_point(aes(fill = SNP.MAG05det), shape = 21, color = "black", 
             size = 3, position = position_jitterdodge(jitter.width = 0.25))+
  geom_boxplot(aes(fill = SNP.MAG05det), outlier.colour = NA, alpha = 0.25)+
  ggsignif::geom_signif(comparisons = list(c("Mm.FALSE","MM.FALSE"),c("Mm.TRUE","MM.TRUE")))+
  ggsignif::geom_signif(y_position = 11, xmin = 1.5, xmax = 3.5, annotations = c(0.084), tip_length = 0)+
  scale_x_discrete(labels = c("AG\nMAG05 absent","AA\nMAG05 absent","AG\nMAG05 present","AA\nMAG05 present"))+
  scale_y_continuous(limits = c(4.5,11.4))+
  scale_fill_manual(values = rep("grey50",4))+
  labs(y = "Gene expression",
       x = "Genotype at position 1548778 + \nMAG05 detection",
       title = "LOC106604312 (ANKHD1-like)")+
  theme_classic()+
  theme(axis.text = element_text(color = "black", size = 12), legend.position = "none",
        axis.title = element_text(size = 13), strip.text.x = element_text(size = 10),
        plot.title = element_text(size = 13, color = "black", hjust = 0.5, face = "bold"),
        panel.grid.major.y = element_line(colour = "grey70", linetype = 2, size = 1), panel.ontop = FALSE)
```

**Fig. 3D**. MAG05 Mycoplasma GWAS results. Gene
expression (normalised and scaled counts) of ANKHD1, the only MAG05
associated gene for which comparable transcriptomic data was available.
Samples have been divided by MAG05 presence/absence and genotype at SNP
15487786 (Mm: major/minor, MM: major/major; minor/minor individuals were
excluded due to low sample numbers). P values are shown for Mm vs MM and
MAG05 absent vs present gene expression comparisons using the Wilcoxon
rank sum test.

```
# for composite plot, remove some labels (added using Inkscape afterwards)
fig3d <-
ggplot(fig3d.df, aes(SNP.MAG05det, abundance))+
  geom_point(aes(fill = SNP.MAG05det), shape = 21, color = "black", size = 3, position = position_jitterdodge(jitter.width = 0.25))+
  geom_boxplot(aes(fill = SNP.MAG05det), outlier.colour = NA, alpha = 0.25)+
  ggsignif::geom_signif(comparisons = list(c("Mm.FALSE","MM.FALSE"),c("Mm.TRUE","MM.TRUE")))+
  ggsignif::geom_signif(y_position = 11, xmin = 1.5, xmax = 3.5, annotations = c(0.084), tip_length = 0)+
  scale_x_discrete(labels = c("Mm","MM","Mm","MM"))+
  scale_y_continuous(limits = c(4.5,11.4))+
  scale_fill_manual(values = rep("grey50",4))+
  labs(y = "Gene expression", x = "")+ 
  theme_classic()+
  theme(axis.text = element_text(color = "black", size = 12), legend.position = "none",
        axis.title = element_text(size = 13), strip.text.x = element_text(size = 10),
        plot.title = element_text(size = 13, color = "black", hjust = 0.5, face = "bold"),
        panel.grid.major.y = element_line(colour = "grey70", linetype = 2, size = 1), panel.ontop = FALSE)
```

### Figure 3 composite

```
# missing heatmap (fig3c) as it's not compatible with cowplot - added in with Inkscape afterwards.
plot_grid(fig3a,
          fig3b,
          NULL,
          fig3d,
          align = "hv", axis = "ltb",
          nrow = 2,
          labels = c("A","B","C","D"))
```

```
# ggsave("figures/Figure_gwas_MAG05chr5.png", units = "in", dpi = 800, height = 7, width = 10)
```

### Differential expression

Are there any DEGs associated with MAG05 and/or the chr5 peak SNPs
within 1 Mbp of peak?

Three models in Supplementary tables:

- model 1: chr 5 SNP calls + feed type + size class + tapeworm
  detection
- model 2: MAG05 detection + feed type + size class + tapeworm
  detection
- model 3: chr 5 SNP calls + MAG05 detection + feed type + size class
  + tapeworm detection

```
# Set up RNA data within 1 Mbp window on chr5
MAG05.chr5.1Mb.gene <- read.delim("output_GWAS/MAG05_presence_sigSNPs_1Mb_anno_gene.txt", header = T)
# MAG05.chr5.1Mb.mrna <- read.delim("output_GWAS/MAG05_presence_sigSNPs_1Mb_anno_mRNA.txt", header = T)

MAG05.chr5.1Mb.genesym <- data.frame(
  gene.name=row.names(rna.vst.data),
  chr=sapply(row.names(rna.vst.data), function(x){ strsplit(x, split = "\\+")[[1]][1] }),
  sym=sapply(row.names(rna.vst.data), function(x){ strsplit(x, split = "\\+")[[1]][2] })
)
MAG05.chr5.1Mb.genesym <- subset(MAG05.chr5.1Mb.genesym, chr == "NC_059446.1")
MAG05.chr5.1Mb.genesym <- MAG05.chr5.1Mb.genesym[which(MAG05.chr5.1Mb.genesym$sym %in% unique(MAG05.chr5.1Mb.gene$GeneSymbol)),"gene.name"]

# Set up metadata, including SNP calls
MAG05.chr5.meta <- MAG05.chr5.geno.call[which(MAG05.chr5.geno.call$marker %in% MAG05.chr5.anno$rs),]
row.names(MAG05.chr5.meta) <- MAG05.chr5.meta$marker
MAG05.chr5.meta <- as.data.frame(t(MAG05.chr5.meta[,-1]))
MAG05.chr5.meta$Fish.ID <- row.names(MAG05.chr5.meta)

## exclude mm from analysis, as too low sample size
MAG05.chr5.meta[MAG05.chr5.meta == "mm"] <- NA
MAG05.chr5.meta$exclude.mm <- rowSums(is.na(MAG05.chr5.meta[,grep("NC",names(MAG05.chr5.meta))])) > 0
MAG05.chr5.meta <- merge(meta[,c("Fish.ID","Bin.pa.MAG05",metavar.fish,metavar.exp,"HostTranscriptome")], MAG05.chr5.meta,
                         by = "Fish.ID", all.x = F)
```

```
# Model 1: SNP calls + covariates
MAG05.chr5.deseq.m1.meta <- MAG05.chr5.meta[which(MAG05.chr5.meta$HostTranscriptome & !MAG05.chr5.meta$exclude.mm),]
MAG05.chr5.deseq.m1.meta <- MAG05.chr5.deseq.m1.meta[order(MAG05.chr5.deseq.m1.meta$Fish.ID),]
row.names(MAG05.chr5.deseq.m1.meta) <- MAG05.chr5.deseq.m1.meta$Fish.ID
## note design breaks if two variables are identical
## names(which(duplicated(t(MAG05.chr5.deseq.m1.design[,2:15]))))
## "NC_059446.1_16600137MM" "NC_059446.1_16875542MM"
## exclude from design
MAG05.chr5.deseq.m1.design <- model.matrix(~ NC_059446.1_15487786 + NC_059446.1_16099761 +
                                             NC_059446.1_16256914 + NC_059446.1_16274373 +
                                             NC_059446.1_16363100 + NC_059446.1_16935347 +
                                             NC_059446.1_16736455 + NC_059446.1_16834879 + 
                                             NC_059446.1_16955542 + NC_059446.1_17334765 +
                                             NC_059446.1_17347221 + NC_059446.1_17359592 +
                                             #NC_059446.1_16600137 + NC_059446.1_16875542 +
                                             Feed.Type + Size.class + Tapeworm.bin, 
                                           data = MAG05.chr5.deseq.m1.meta)

MAG05.chr5.deseq.m1.res <- run_deseq_f(
  ct.df = rna.gene.cts[MAG05.chr5.1Mb.genesym,row.names(MAG05.chr5.deseq.m1.design)],
  meta.df = MAG05.chr5.deseq.m1.meta,
  design.df = MAG05.chr5.deseq.m1.design,
  sig.level = 0.05,
  output.ext = "output_DESeq/HostRNA_DESeq_chr5_1Mb_M1_SNPcalls"
)
```

```
## [1] "No. of genes significantly associated at p < 0.05 with each variable:"
## [1] "Feed.TypeFeed2 - 15"
## [1] "Size.class.L - 2"
```

```
# Model 2: MAG05 detection + covariates
MAG05.chr5.deseq.m2.meta <- MAG05.chr5.meta[which(MAG05.chr5.meta$HostTranscriptome & !is.na(MAG05.chr5.meta$Bin.pa.MAG05)),]
MAG05.chr5.deseq.m2.meta <- MAG05.chr5.deseq.m2.meta[order(MAG05.chr5.deseq.m2.meta$Fish.ID),]
row.names(MAG05.chr5.deseq.m2.meta) <- MAG05.chr5.deseq.m2.meta$Fish.ID

MAG05.chr5.deseq.m2.design <- model.matrix(~ Bin.pa.MAG05 +
                                             Feed.Type + Size.class + Tapeworm.bin, 
                                           data = MAG05.chr5.deseq.m2.meta)

MAG05.chr5.deseq.m2.res <- run_deseq_f(
  ct.df = rna.gene.cts[MAG05.chr5.1Mb.genesym,row.names(MAG05.chr5.deseq.m2.design)],
  meta.df = MAG05.chr5.deseq.m2.meta,
  design.df = MAG05.chr5.deseq.m2.design,
  sig.level = 0.05,
  output.ext = "output_DESeq/HostRNA_DESeq_chr5_1Mb_M2_MAG05"
)
```

```
## [1] "No. of genes significantly associated at p < 0.05 with each variable:"
## [1] "Feed.TypeFeed2 - 17"
## [1] "Size.class.L - 4"
```

```
# Model 3: SNP calls + MAG05 detection + covariates
MAG05.chr5.deseq.m3.meta <- MAG05.chr5.meta[which(MAG05.chr5.meta$HostTranscriptome & 
                                                    !MAG05.chr5.meta$exclude.mm &
                                                    !is.na(MAG05.chr5.meta$Bin.pa.MAG05)),]
MAG05.chr5.deseq.m3.meta <- MAG05.chr5.deseq.m3.meta[order(MAG05.chr5.deseq.m3.meta$Fish.ID),]
row.names(MAG05.chr5.deseq.m3.meta) <- MAG05.chr5.deseq.m3.meta$Fish.ID

MAG05.chr5.deseq.m3.design <- model.matrix(~ NC_059446.1_15487786 + NC_059446.1_16099761 +
                                             NC_059446.1_16256914 + NC_059446.1_16274373 +
                                             NC_059446.1_16363100 + NC_059446.1_16935347 +
                                             NC_059446.1_16736455 + NC_059446.1_16834879 + 
                                             NC_059446.1_16955542 + NC_059446.1_17334765 +
                                             NC_059446.1_17347221 + NC_059446.1_17359592 +
                                             #NC_059446.1_16600137 + NC_059446.1_16875542 +
                                             Bin.pa.MAG05 +
                                             Feed.Type + Size.class + Tapeworm.bin, 
                                           data = MAG05.chr5.deseq.m3.meta)

MAG05.chr5.deseq.m3.res <- run_deseq_f(
  ct.df = rna.gene.cts[MAG05.chr5.1Mb.genesym,row.names(MAG05.chr5.deseq.m3.design)],
  meta.df = MAG05.chr5.deseq.m3.meta,
  design.df = MAG05.chr5.deseq.m3.design,
  sig.level = 0.05,
  output.ext = "output_DESeq/HostRNA_DESeq_chr5_1Mb_M3_SNPcall_MAG05"
)
```

```
## [1] "No. of genes significantly associated at p < 0.05 with each variable:"
## [1] "NC_059446.1_16099761MM - 1"
## [1] "NC_059446.1_16256914MM - 1"
## [1] "NC_059446.1_16274373MM - 2"
## [1] "Feed.TypeFeed2 - 14"
```

### QC

Summary of RNA integrity numbers (RINs) of sequenced samples:

```
summary(rna.mapping.stats$RNA.extract.RIN)
```

```
##    Min. 1st Qu.  Median    Mean 3rd Qu.    Max.    NA's 
##   0.000   2.400   2.500   2.599   2.600   9.600       6
```

#### Supp figure

Coverage curves of genes of interest (identified in GWAS above and
MOFA below) before and after DegNorm normalisation for RNA degradation -
only including samples used for analysis:

- GWAS: LOC106604312 = ANKHD1 ankyrin 232 repeat and KH
  domain-containing protein 1-like
- MOFA-cestode: GADL1 = glutamate decarboxylase like 1 [taurine
  biosynthesis]
- MOFA-cestode: LOC106576775 = CSAD cysteine sulfinic acid
  decarboxylase-like [taurine biosynthesis]
- MOFA-size/Vibrio: LOC106576106 = ladderlectin [immune pathway]
- MOFA-size/Vibrio: LOC106586541 = APOA1 apolipoprotein A-I
  [apolipoprotein]
- MOFA-size/Vibrio: LOC106564730 = actin, alpha skeletal muscle 2
  [ECM]

```
# GWAS gene: LOC106604312 (ANKHD1-like)
dn.ANKHD1 <- read.csv("data/HostRNA/DegNorm_coverage_LOC106604312.csv")
dn.ANKHD1$Step <- factor(dn.ANKHD1$Step, levels = c("Original","Normalised"))
dn.ANKHD1 <- subset(dn.ANKHD1, Fish.ID %in% rna.samples.50)

p.ANKHD1 <-
ggplot(dn.ANKHD1, aes(Position, coverage, group = Fish.ID))+
  geom_line(color = "grey50", alpha = 0.5)+
  labs(x = "Position in gene", y = "Coverage", title = "ANKHD1-like")+
  theme_classic()+
  theme(axis.text = element_text(color = "black", size = 10), legend.position = "none", 
        axis.title = element_text(size = 12),
        plot.title = element_text(size = 13, color = "black", hjust = 0.5, face = "bold"))+
  facet_wrap(~ Step)

# MOFA gene (cestode, taurine): LOC106576775 (CSAD-like)
dn.CSAD <- read.csv("data/HostRNA/DegNorm_coverage_LOC106576775.csv")
dn.CSAD$Step <- factor(dn.CSAD$Step, levels = c("Original","Normalised"))
dn.CSAD <- subset(dn.CSAD, Fish.ID %in% rna.samples.50)

p.CSAD <-
ggplot(dn.CSAD, aes(Position, coverage, group = Fish.ID))+
  geom_line(color = "grey50", alpha = 0.5)+
  labs(x = "Position in gene", y = "Coverage", title = "CSAD-like")+
  theme_classic()+
  theme(axis.text = element_text(color = "black", size = 10), legend.position = "none", 
        axis.title = element_text(size = 12),
        plot.title = element_text(size = 13, color = "black", hjust = 0.5, face = "bold"))+
  facet_wrap(~ Step)

# MOFA gene (cestode, taurine): GADL1
dn.GADL1 <- read.csv("data/HostRNA/DegNorm_coverage_GADL1.csv")
dn.GADL1$Step <- factor(dn.GADL1$Step, levels = c("Original","Normalised"))
dn.GADL1 <- subset(dn.GADL1, Fish.ID %in% rna.samples.50)

p.GADL1 <-
ggplot(dn.GADL1, aes(Position, coverage, group = Fish.ID))+
  geom_line(color = "grey50", alpha = 0.5)+
  labs(x = "Position in gene", y = "Coverage", title = "GADL1")+
  theme_classic()+
  theme(axis.text = element_text(color = "black", size = 10), legend.position = "none", 
        axis.title = element_text(size = 12),
        plot.title = element_text(size = 13, color = "black", hjust = 0.5, face = "bold"))+
  facet_wrap(~ Step)

# MOFA gene (size, immune): LOC106576106 (ladderlectin)
dn.ladder <- read.csv("data/HostRNA/DegNorm_coverage_LOC106576106.csv")
dn.ladder$Step <- factor(dn.ladder$Step, levels = c("Original","Normalised"))
dn.ladder <- subset(dn.ladder, Fish.ID %in% rna.samples.50)

p.ladder <-
ggplot(dn.ladder, aes(Position, coverage, group = Fish.ID))+
  geom_line(color = "grey50", alpha = 0.5)+
  labs(x = "Position in gene", y = "Coverage", title = "Ladderlectin")+
  theme_classic()+
  theme(axis.text = element_text(color = "black", size = 10), legend.position = "none", 
        axis.title = element_text(size = 12),
        plot.title = element_text(size = 13, color = "black", hjust = 0.5, face = "bold"))+
  facet_wrap(~ Step)

# MOFA gene (size, apolipoprotein): LOC106586541 (APOA1)
dn.apoli <- read.csv("data/HostRNA/DegNorm_coverage_LOC106586541.csv")
dn.apoli$Step <- factor(dn.apoli$Step, levels = c("Original","Normalised"))
dn.apoli <- subset(dn.apoli, Fish.ID %in% rna.samples.50)

p.apoli <-
ggplot(dn.apoli, aes(Position, coverage, group = Fish.ID))+
  geom_line(color = "grey50", alpha = 0.5)+
  labs(x = "Position in gene", y = "Coverage", title = "APOA1")+
  theme_classic()+
  theme(axis.text = element_text(color = "black", size = 10), legend.position = "none", 
        axis.title = element_text(size = 12),
        plot.title = element_text(size = 13, color = "black", hjust = 0.5, face = "bold"))+
  facet_wrap(~ Step)

# MOFA gene (size, ECM): LOC106564730 (actin)
dn.actin <- read.csv("data/HostRNA/DegNorm_coverage_LOC106564730.csv")
dn.actin$Step <- factor(dn.actin$Step, levels = c("Original","Normalised"))
dn.actin <- subset(dn.actin, Fish.ID %in% rna.samples.50)

p.actin <-
ggplot(dn.actin, aes(Position, coverage, group = Fish.ID))+
  geom_line(color = "grey50", alpha = 0.5)+
  labs(x = "Position in gene", y = "Coverage", title = "Actin")+
  theme_classic()+
  theme(axis.text = element_text(color = "black", size = 10), legend.position = "none", 
        axis.title = element_text(size = 12),
        plot.title = element_text(size = 13, color = "black", hjust = 0.5, face = "bold"))+
  facet_wrap(~ Step)
```

```
cowplot::plot_grid(p.GADL1,p.CSAD,p.ladder,p.apoli,p.actin,p.ANKHD1, 
                   axis = "tblr", align = "hv", nrow = 3,
                   labels = c("A","B","C","D","E","F"))
```

```
ggsave("figures/Supp_figure_hostRNA_DegNorm_coverage_plots_genes_of_interest.png", units = "in", dpi = 800, width = 9, height = 11)
```

**Fig. S11**. RNA coverage curves before and after
normalisation by DegNorm for each of the 343 samples included in the
final RNA analyses, for six genes of interest, based on the GWAS and
MOFA analyses. (**A**) Glutamate decarboxylase like 1
(GADL1) and (**B**) cysteine sulfinic acid
decarboxylase-like (CSAD-like), two genes involved in taurine
biosynthesis that were associated with cestode presence and Mycoplasma
MAGs in the MOFA (factor 3 in Fig. 4). (**C**) Ladderlectin
and (**D**) apolipoprotein A-I (APOA1), two genes involved
in immune-related pathways that were associated with small fish and
Vibrionaceae MAGs in the MOFA (factor 7 in Fig. 4). (**E**)
Actin, alpha skeletal muscle 2 (Actin), a gene involved in cell division
and the cytoskeleton that was associated with large fish in the MOFA
(factor7 in Fig. 4). (**F**) Ankyrin repeat and KH
domain-containing protein 1-like (ANKHD1-like), which contained a SNP
that was associated with MAG05 detection in the GWAS (Fig. 3).

## Metabolomics

**Processing steps**:

- Removed metabolites present in < 50% of fish samples, to assist
  with zero inflation problems
- Removed samples with > 80% of metabolites not detected
- Converted 0 abundances to NAs, as I believe it’s more accurate to do
  this, then impute, rather than assigning missing values as
  0/pseudo0

Analysis with MetaboDiff:

- For metaboDiff:
  - Imputed values for all metabolites with NAs in < 40% of samples
    (except there aren’t any in this dataset)
  - Outlier removal based on cluster analysis (see heatmap below)
  - Default normalisation

```
# abundance data
mbol.raw <- read.csv("data/Metabolome/Metabolome_feature_table.csv")

# remove feature info and transform so that samples = rows
row.names(mbol.raw) <- mbol.raw$row.ID
mbol.fish <- as.data.frame(t(mbol.raw[,4:ncol(mbol.raw)]))

# Currently not-detected metabolites given 0 abundance
# Based on this paper: https://bmcbioinformatics.biomedcentral.com/articles/10.1186/s12859-019-3250-2
# I think it is more correct to assign not-detected metabolites as NA and use imputation, instead of 0 abundance, or a pseudozero count
mbol.fish[mbol.fish == 0] <- NA

# Filter out metabolites present in < 50% of the samples
# (actually, in this data, for a metabolite, max 38% of samples are NAs, so either threshold makes no difference)
mbol.fish <- mbol.fish[, which(!colMeans(is.na(mbol.fish)) > 0.5)]

# Remove samples with > 80% NAs, as not enough data for big analyses
# (again, in this data, for a sample, max 21% of metabolites are NAs, so no filtering necessary)
mbol.fish <- mbol.fish[which(!rowMeans(is.na(mbol.fish)) > 0.8),]

# make sure sample orders are identical everywhere - match to mbol.fish
mbol.meta <- meta[which(meta$Fish.ID %in% row.names(mbol.fish)),
                  c("Fish.ID",metavar.fish,metavar.exp,"Mbol.batch")]
mbol.meta <- mbol.meta[match(row.names(mbol.fish),mbol.meta$Fish.ID),]
row.names(mbol.meta) <- mbol.meta$Fish.ID

# metabolite classification data
## add NAs into unannotated metabolites
mbol.anno <- read.delim("data/Metabolome/FT_Classification.txt")

mbol.anno$FT_ID <- gsub("^","FT_",mbol.anno$FT_ID)
mbol.anno <- merge(mbol.anno, data.frame(FT_ID=names(mbol.fish)), by = "FT_ID", all = T)
mbol.anno <- mbol.anno[match(names(mbol.fish),mbol.anno$FT_ID),]
row.names(mbol.anno) <- mbol.anno$FT_ID
```

```
# get mbol data in format for MetaboDiff
#rowData
mbol.chemdata <- mbol.anno[,c("FT_ID","superclass","class","subclass","level.5","most.specific.class")]
names(mbol.chemdata) <- c("FT_ID","R1.superclass","R2.class","R3.subclass","R4.level5","R5.specific")
mbol.chemdata$BIOCHEMICAL <- mbol.chemdata$FT_ID #seems required for some downstream analysis
row.names(mbol.chemdata) <- mbol.chemdata$FT_ID

#colData - mbol.meta

# met object
mbol.met <- create_mae(t(data.matrix(mbol.fish)), mbol.chemdata, mbol.meta)

## check for missing values & imputation
na_heatmap(mbol.met, group_factor="Mbol.batch", label_colors=gg_color_hue(4))
```

```
# impute
mbol.met = knn_impute(mbol.met, cutoff=0.4)
outlier_heatmap(mbol.met, group_factor="Mbol.batch", label_colors=gg_color_hue(4), k=2)
```

```
# one cluster of samples with small abundances - doesn't correlate with # of NAs per sample, although most of these samples are missing some number of metabolites
# some clustering by batch, of course
# remove this cluster (cluster 2) to improve normalisation and downstream unsupervised analysis

mbol.met <- remove_cluster(mbol.met, cluster=2)

## normalisation
mbol.met <- normalize_met(mbol.met)
quality_plot(mbol.met, group_factor="Mbol.batch", label_colors=gg_color_hue(4))
```

```
# update meta to exclude outlier samples
mbol.meta <- mbol.meta[which(mbol.meta$Fish.ID %in% mbol.met$Fish.ID),]
```

### Ordination

```
# PCA
mbol.pca <- prcomp(t(assay(mbol.met[["norm_imputed"]])))

mbol.pca.coord <- as.data.frame(get_pca_ind(mbol.pca)$coord)
mbol.pca.coord$Fish.ID <- row.names(mbol.pca.coord)
mbol.pca.coord <- merge(mbol.pca.coord[,c("Fish.ID","Dim.1","Dim.2","Dim.3")], mbol.meta, by = "Fish.ID", all.x = TRUE)

# get % variance
mbol.pca.eig <- get_eigenvalue(mbol.pca)[c("Dim.1","Dim.2","Dim.3"),]

# get gene contributions
mbol.pca.contrib <- get_pca_var(mbol.pca)$contrib[,c("Dim.1","Dim.2","Dim.3")]

# Supp figure: before batch effect removal
pca.before.batch <-
ggplot(mbol.pca.coord, aes(Dim.1, Dim.2, color = Mbol.batch))+
  geom_point(size=3)+
  scale_color_manual(values = anno_colours$Mbol.batch)+
  labs(x = paste0("PC1: ",round(mbol.pca.eig[1,2],1),"% variance"),
       y = paste0("PC2: ",round(mbol.pca.eig[2,2],1),"% variance"),
       color = "Batch",
       title = "Before removal of batch effects")+
  theme_bw()+
  theme(axis.text = element_text(color = "black", size = 12), axis.title = element_text(size = 13),
        legend.position = "right", legend.text = element_text(size = 12), legend.title = element_text(size = 12),
        plot.title = element_text(color = "black", size = 14, hjust = 0.5))

pca.before.feed <-
ggplot(mbol.pca.coord, aes(Dim.1, Dim.2, color=Feed.Type))+
  geom_point(size=3)+
  scale_color_manual(values = anno_colours$Feed.Type)+
  labs(x = paste0("PC1: ",round(mbol.pca.eig[1,2],1),"% variance"),
       y = paste0("PC2: ",round(mbol.pca.eig[2,2],1),"% variance"),
       color = "Feed type")+
  theme_bw()+
  theme(axis.text = element_text(color = "black", size = 12), axis.title = element_text(size = 13),
        legend.position = "right", legend.text = element_text(size = 12), legend.title = element_text(size = 12))

pca.before.size <-
ggplot(mbol.pca.coord, aes(Dim.1, Dim.2, color=Size.class))+
  geom_point(size=3)+
  scale_color_manual(values = anno_colours$Size.class)+
  labs(x = paste0("PC1: ",round(mbol.pca.eig[1,2],1),"% variance"),
       y = paste0("PC2: ",round(mbol.pca.eig[2,2],1),"% variance"),
       color = "Size class")+
  theme_bw()+
  theme(axis.text = element_text(color = "black", size = 12), axis.title = element_text(size = 13),
        legend.position = "right", legend.text = element_text(size = 12), legend.title = element_text(size = 12))

pca.before.batch
```

```
pca.before.feed
```

```
pca.before.size
```

**PCA of metabolite abundances**, coloured by batch,
feed type and size class. Some clustering by batch evident in PCA -
known problem with metabolomics data. No clear clustering by metadata
variables.

**Removal of batch effects with limma**, for
unsupervised analysis (like ordination, MOFA).

```
# remove batch effects with limma, ready for UNSUPERVISED analysis (should not be used for hypothesis testing)
# see: https://rdrr.io/bioc/limma/man/removeBatchEffect.html

mbol.design.simple <- model.matrix(~ Feed.Type + Size.class + Tapeworm.bin, data = data.frame(colData(mbol.met)))

mbol.nobatch <- removeBatchEffect(assay(mbol.met[["norm_imputed"]]), batch = data.frame(colData(mbol.met))[,"Mbol.batch"],
                                  design = mbol.design.simple)
```

Repeat PCA to see effect.

```
mbol.nb.pca <- prcomp(t(mbol.nobatch))

mbol.nb.pca.coord <- as.data.frame(get_pca_ind(mbol.nb.pca)$coord)
mbol.nb.pca.coord$Fish.ID <- row.names(mbol.nb.pca.coord)
mbol.nb.pca.coord <- merge(mbol.nb.pca.coord[,c("Fish.ID","Dim.1","Dim.2","Dim.3")], mbol.meta, by = "Fish.ID", all.x = TRUE)

# get % variance
mbol.nb.pca.eig <- get_eigenvalue(mbol.nb.pca)[c("Dim.1","Dim.2","Dim.3"),]

# get gene contributions
mbol.nb.pca.contrib <- get_pca_var(mbol.nb.pca)$contrib[,c("Dim.1","Dim.2","Dim.3")]

## Supp figure: after batch effects removal

pca.after.batch <-
ggplot(mbol.nb.pca.coord, aes(Dim.1, Dim.2, color = Mbol.batch))+
  geom_point(size=3)+
  scale_color_manual(values = anno_colours$Mbol.batch)+
  labs(x = paste0("PC1: ",round(mbol.nb.pca.eig[1,2],1),"% variance"),
       y = paste0("PC2: ",round(mbol.nb.pca.eig[2,2],1),"% variance"),
       color = "Batch",
       title = "After removal of batch effects")+
  theme_bw()+
  theme(axis.text = element_text(color = "black", size = 12), axis.title = element_text(size = 13),
        legend.position = "right", legend.text = element_text(size = 12), legend.title = element_text(size = 12),
        plot.title = element_text(color = "black", size = 14, hjust = 0.5))

pca.after.feed <-
ggplot(mbol.nb.pca.coord, aes(Dim.1, Dim.2, color=Feed.Type))+
  geom_point(size=3)+
  scale_color_manual(values = anno_colours$Feed.Type)+
  labs(x = paste0("PC1: ",round(mbol.nb.pca.eig[1,2],1),"% variance"),
       y = paste0("PC2: ",round(mbol.nb.pca.eig[2,2],1),"% variance"),
       color = "Feed type")+
  theme_bw()+
  theme(axis.text = element_text(color = "black", size = 12), axis.title = element_text(size = 13),
        legend.position = "right", legend.text = element_text(size = 12), legend.title = element_text(size = 12))

pca.after.size <-
ggplot(mbol.nb.pca.coord, aes(Dim.1, Dim.2, color=Size.class))+
  geom_point(size=3)+
  scale_color_manual(values = anno_colours$Size.class)+
  labs(x = paste0("PC1: ",round(mbol.nb.pca.eig[1,2],1),"% variance"),
       y = paste0("PC2: ",round(mbol.nb.pca.eig[2,2],1),"% variance"),
       color = "Size class")+
  theme_bw()+
  theme(axis.text = element_text(color = "black", size = 12), axis.title = element_text(size = 13),
        legend.position = "right", legend.text = element_text(size = 12), legend.title = element_text(size = 12))
```

#### Supp figure

```
cowplot::plot_grid(pca.before.batch, pca.after.batch,
                   pca.before.feed, pca.after.feed,
                   pca.before.size, pca.after.size, 
                   align = "hv", axis = "tblr", ncol = 2, labels = c("A","B","C","D","E","F"))
```

```
ggsave("figures/Supp_figure_mbol_PCAs.png", units = "in", dpi = 600, width = 10, height = 13)
```

**Fig. S3.** Ordination (PCA) of gut metabolomes, before
(**A, C, E**) and after (**B, D, F**) removal
of batch effects. Batch effects are a common problem in metabolomic
studies and in this study, metabolome batch explains 11.4% of the
variation in metabolite abundance before correction (F = 14.98, p =
0.001) (A), while after correction, batch does not significantly explain
metabolite abundance variation (B). Feed type consistently explains
2.2-2.5% of the variation (F = 8.62, p = 0.001) (C–D) and size class
explains 3.2-3.6% of the variation (F = 6.39, p = 0.001). Detailed
results from the PERMANOVAs are presented in Table S1. PERMANOVAs were
based on Euclidean distances.

```
# permanovas
mbol.before.dist <- vegdist(t(assay(mbol.met[["norm_imputed"]])), method = "euclidean")

perm.mbol.before <-adonis2(mbol.before.dist ~ Mbol.batch + Feed.Type + Size.class + Tapeworm,
                                mbol.meta[match(mbol.meta$Fish.ID,colnames(assay(mbol.met[["norm_imputed"]]))),],
                                permutations = 999, method = "euclidean")
perm.mbol.before
```

```
## Permutation test for adonis under reduced model
## Terms added sequentially (first to last)
## Permutation: free
## Number of permutations: 999
## 
## adonis2(formula = mbol.before.dist ~ Mbol.batch + Feed.Type + Size.class + Tapeworm, data = mbol.meta[match(mbol.meta$Fish.ID, colnames(assay(mbol.met[["norm_imputed"]]))), ], permutations = 999, method = "euclidean")
##             Df SumOfSqs      R2       F Pr(>F)    
## Mbol.batch   3   196273 0.11362 14.9827  0.001 ***
## Feed.Type    1    37622 0.02178  8.6157  0.001 ***
## Size.class   2    55827 0.03232  6.3924  0.001 ***
## Tapeworm     3    22984 0.01330  1.7545  0.004 ** 
## Residual   324  1414796 0.81898                   
## Total      333  1727502 1.00000                   
## ---
## Signif. codes:  0 '***' 0.001 '**' 0.01 '*' 0.05 '.' 0.1 ' ' 1
```

```
mbol.after.dist <- vegdist(t(mbol.nobatch), method = "euclidean")

perm.mbol.after <- adonis2(mbol.after.dist ~ Mbol.batch + Feed.Type + Size.class + Tapeworm,
                           mbol.meta[match(mbol.meta$Fish.ID,colnames(mbol.nobatch)),],
                           permutations = 999, method = "euclidean")
perm.mbol.after
```

```
## Permutation test for adonis under reduced model
## Terms added sequentially (first to last)
## Permutation: free
## Number of permutations: 999
## 
## adonis2(formula = mbol.after.dist ~ Mbol.batch + Feed.Type + Size.class + Tapeworm, data = mbol.meta[match(mbol.meta$Fish.ID, colnames(mbol.nobatch)), ], permutations = 999, method = "euclidean")
##             Df SumOfSqs      R2      F Pr(>F)    
## Mbol.batch   3     1307 0.00085 0.0998  1.000    
## Feed.Type    1    37622 0.02455 8.6157  0.001 ***
## Size.class   2    55827 0.03643 6.3924  0.001 ***
## Tapeworm     3    22984 0.01500 1.7545  0.004 ** 
## Residual   324  1414796 0.92317                  
## Total      333  1532536 1.00000                  
## ---
## Signif. codes:  0 '***' 0.001 '**' 0.01 '*' 0.05 '.' 0.1 ' ' 1
```

```
# format to print as table
perm.mbol.before.df <- as.data.frame(perm.mbol.before)
perm.mbol.before.df$Variable <- row.names(perm.mbol.before.df)
perm.mbol.before.df$Dataset <- "Mbol.before"
row.names(perm.mbol.before.df) <- NULL

perm.mbol.after.df <- as.data.frame(perm.mbol.after)
perm.mbol.after.df$Variable <- row.names(perm.mbol.after.df)
perm.mbol.after.df$Dataset <- "Mbol.after"
row.names(perm.mbol.after.df) <- NULL

### write all permanovas into one table
if(!file.exists("tables/Supp_table_permanovas.csv")){
  perm.all <- rbind.data.frame(
    fatty.acid.perm.df,
    perm.euc.df,
    perm.PAjac.df,
    perm.rna.df,
    perm.mbol.before.df,
    perm.mbol.after.df
  )
  write.csv(perm.all[,c(7,6,1:5)],
            "tables/Supp_table_permanovas.csv",
            row.names = F, quote = F)
  rm(perm.all)
}
```

```
# add additional metabolite annotation for MOFA analysis
mbol.chemdata$top.anno <- sapply(mbol.chemdata$R1.superclass, function(x){
  if(is.na(x)){ "unknown" }
  else if(x == "Benzenoids") { "Benzenoids" }
  else if(x == "Lipids and lipid-like molecules") { "Lipids" }
  else if(x == "Organic acids and derivatives") { "Organic.acids" }
  else if(x == "Organic nitrogen compounds") { "Nitrogens" }
  else if(x == "Organic oxygen compounds") { "Oxygens" }
  else if(x == "Organohalogen compounds") { "Organohalogens" }
  else if(x == "Organoheterocyclic compounds") { "Organoheterocyclics" }
  else { "rare.other" }
})
row.names(mbol.chemdata) <- mbol.chemdata$FT_ID
```

## Multi-omics integration

Using MOFA2 to
integrate MAG detection, Host RNA and Metabolome datasets.

Useful documentation:

- http://www.bioconductor.org/packages/release/bioc/vignettes/MOFA2/inst/doc/getting\_started\_R.html
- http://htmlpreview.github.io/?https://github.com/bioFAM/MOFA2\_tutorials/blob/master/R\_tutorials/CLL.html
- https://muon-tutorials.readthedocs.io/en/latest/CLL.html

Feature selection:

- Metagenome:
  - MAG presence/absence
  - microbiome presence/absence phenotypes
- Host RNA
  - 500 most variable genes (2.56% of genes in dataset)
  - after VST normalisation
- Metabolome:
  - 500 most variable metabolites (142.05% of metabolites in
    dataset)
  - after normalisation
  - after removal of batch effects

```
# metagenome
# MAG presence/absence + microbiome presence/absence phenotypes
bin.pa.for.mofa <- merge(bin.PA[,c("Fish.ID",bins.included.pa)],
                         meta[,c("Fish.ID","Bin.pa.Myco","Bin.pa.Photo","Bin.pa.Ali","Bin.pa.Msal.high")],
                         by = "Fish.ID", all.y = F)
row.names(bin.pa.for.mofa) <- bin.pa.for.mofa$Fish.ID
bin.pa.for.mofa <- bin.pa.for.mofa[,-1]

# host RNA
rna.var.genes <- rownames(rna.vst.data)[order(apply(rna.vst.data, 1, var), decreasing = T)][1:500]

# metabolites
mbol.nobatch <- mbol.nobatch[,order(colnames(mbol.nobatch))]
mbol.var.mbolites <- rownames(mbol.nobatch)[order(apply(mbol.nobatch, 1, var), decreasing = T)][1:500]
```

### Combined model

For all samples, not separated by feed type.

```
# some package incompatibilities, try to have as few packages loaded as possible for this analysis
rm(bin.PA.ps, bin.ps, bin.ps.log, bin.taxa.ps, 
   m16S.ps, m16S.ps.blanks, m16S.ps.blanks.relab, m16S.ps.clr, m16S.ps.filt,
   m16S.ps.gut, m16S.ps.relab)
unloadNamespace("microbiome")
unloadNamespace("phyloseq")


library(basilisk)
library(MOFA2)

# MOFA
mofa.input <- rbind.data.frame(
  cbind.data.frame(
    melt(as.matrix(bin.pa.for.mofa),varnames = c("sample","feature")),
    data.frame(view = "MetaG")),
  cbind.data.frame(
    melt(t(mbol.nobatch[mbol.var.mbolites,]),varnames = c("sample","feature")),
    data.frame(view = "mbol")),
  cbind.data.frame(
    melt(t(rna.vst.data[rna.var.genes,]),varnames = c("sample","feature")),
    data.frame(view = "HostRNA"))
)
# table(mofa.input$view)

row.names(meta) <- meta$Fish.ID
mofa.trained <- run_mofa_default(
  df.input = mofa.input, 
  outfile = "output_MOFA/mofa_results_combined", 
  metadata = meta,
  covar = c("Feed.Type","Size.class","Tapeworm.bin","Mbol.batch"),
  view.distrib = c("gaussian","gaussian","bernoulli"),
  num_factors = 15
)
```

```
plot_variance_explained(mofa.trained$trained.obj, plot_total = T)[[2]]
```

```
plot_variance_explained(mofa.trained$trained.obj, x="view", y="factor")
```

```
mofa.trained$corr$Factor <- factor(mofa.trained$corr$Factor, 
                                   levels = unique(mofa.trained$corr$Factor)[c(1,8:15,2:7)])
ggplot(mofa.trained$corr, aes(Phenotype, Factor, fill = cor.logp))+
  geom_tile()+
  scale_fill_gradient(low = "white", high = "red")+
  theme_bw()+
  theme(title = element_text(size = 16), 
        axis.text = element_text(size = 14, colour = "black"),
        legend.text = element_text(size = 14, colour = "black"))
```

```
mofa.sample.weights <- merge(
  mofa.trained$sample.weights, 
  meta[,c("Fish.ID","Feed.Type","Size.class","Tapeworm.bin","Mbol.batch")],
  by.x = "sample", by.y = "Fish.ID", all = F
)
```

```
# annotate features by functional categories (defined in annotate_mofa_f function)
mofa.anno.mgx <- annotate_mofa_f(mofa.trained$feature.weights, "MetaG",
                                 c("Factor1","Factor2","Factor3","Factor7"), threshold = 0.2)

mofa.anno.tx <- annotate_mofa_f(mofa.trained$feature.weights, "HostRNA",
                                c("Factor1","Factor2","Factor3","Factor7"), threshold = 0.2)

mofa.anno.mbol <- annotate_mofa_f(mofa.trained$feature.weights, "mbol",
                                  c("Factor1","Factor2","Factor3","Factor7"), threshold = 0.2)
```

### Feed1 model

Repeat MOFA for Feed1 samples only.

```
samples.feed1 <- meta[which(meta$Feed.Type == "Feed1"),"Fish.ID"]

mofa.input.feed1 <- mofa.input[which(mofa.input$sample %in% samples.feed1),]
# table(mofa.input.feed1$view)

mofa.trained.feed1 <- run_mofa_default(
  df.input = mofa.input.feed1, 
  outfile = "output_MOFA/mofa_results_feed1", 
  metadata = meta,
  covar = c("Size.class","Tapeworm.bin","Mbol.batch"),
  view.distrib = c("gaussian","gaussian","bernoulli"),
  num_factors = 15
)
```

```
plot_variance_explained(mofa.trained.feed1$trained.obj, plot_total = T)[[2]]
```

```
plot_variance_explained(mofa.trained.feed1$trained.obj, x="view", y="factor")
```

```
mofa.trained.feed1$corr$Factor <- factor(mofa.trained.feed1$corr$Factor, 
                                   levels = unique(mofa.trained.feed1$corr$Factor)[c(1,8:15,2:7)])
ggplot(mofa.trained.feed1$corr, aes(Phenotype, Factor, fill = cor.logp))+
  geom_tile()+
  scale_fill_gradient(low = "white", high = "red")+
  theme_bw()+
  theme(title = element_text(size = 16), 
        axis.text = element_text(size = 14, colour = "black"),
        legend.text = element_text(size = 14, colour = "black"))
```

```
mofa.f1.sample.weights <- merge(
  mofa.trained.feed1$sample.weights, 
  meta[,c("Fish.ID","Feed.Type","Size.class","Tapeworm.bin","Mbol.batch")],
  by.x = "sample", by.y = "Fish.ID", all = F
)
```

```
# annotate features by functional categories (defined in annotate_mofa_f function)
mofa.anno.mgx.f1 <- annotate_mofa_f(mofa.trained.feed1$feature.weights, "MetaG",
                                    c("Factor1","Factor2","Factor3","Factor6","Factor7"), threshold = 0.2)

mofa.anno.tx.f1 <- annotate_mofa_f(mofa.trained.feed1$feature.weights, "HostRNA",
                                   c("Factor1","Factor2","Factor3","Factor6","Factor7"), threshold = 0.2)

mofa.anno.mbol.f1 <- annotate_mofa_f(mofa.trained.feed1$feature.weights, "mbol",
                                     c("Factor1","Factor2","Factor3","Factor6","Factor7"), threshold = 0.2)
```

#### Supp figure

Comparisons with combined model:

- Factor1 (RNA, correlated with size class) is similar to Factor2 in
  combined model
- Factor2 (metabolome, correlated with no fish phenotypes) is similar
  to Factor1 in combined model
- Factor3 (metagenome, correlated with tapeworm detection) is similar
  to Factor3 in combined model
- Factor6 (metagenome, RNA) is somewhat similar to Factor7 in combined
  model
- Factor7 (metagenome, metabolome) is also somewhat similar to Factor7
  in combined model

```
# order features by weight
mofa.anno.mgx.f1$rank <- paste(mofa.anno.mgx.f1$feature,mofa.anno.mgx.f1$factor, sep = "_")
mofa.anno.mgx.f1$rank <- factor(mofa.anno.mgx.f1$rank, 
                                levels = mofa.anno.mgx.f1[order(mofa.anno.mgx.f1$value, decreasing = F),"rank"])
mofa.anno.mbol.f1$rank <- paste(mofa.anno.mbol.f1$feature,mofa.anno.mbol.f1$factor, sep = "_")
mofa.anno.mbol.f1$rank <- factor(mofa.anno.mbol.f1$rank, 
                                 levels = mofa.anno.mbol.f1[order(mofa.anno.mbol.f1$value, decreasing = F),"rank"])
mofa.anno.tx.f1$rank <- paste(mofa.anno.tx.f1$feature,mofa.anno.tx.f1$factor, sep = "_")
mofa.anno.tx.f1$rank <- factor(mofa.anno.tx.f1$rank, 
                               levels = mofa.anno.tx.f1[order(mofa.anno.tx.f1$value, decreasing = F),"rank"])
```

```
# table(mofa.anno.mgx.f1$anno.figure)
# summary(mofa.anno.mgx.f1$value)

mofa.anno.mgx.f1$anno.figure <- factor(mofa.anno.mgx.f1$anno.figure, 
                                      levels = c("M.salmoninae","mycoplasma","Photobacterium","Aliivibrio",""))
# Aliivibrio not in sig. MAGs

feed1.figMG <-
ggplot(mofa.anno.mgx.f1, aes(rank, value))+
    geom_hline(yintercept = 0.2, color = "grey50", linetype = 2)+
    geom_hline(yintercept = -0.2, color = "grey50", linetype = 2)+
    geom_point(color = "grey60", size = 1, alpha = 0.7, shape = 16)+
    geom_point(aes(color = anno.figure, alpha = anno.figure, size = anno.figure), shape = 16)+
    scale_color_manual(values = c(RColorBrewer::brewer.pal(3,"Set1"),"grey60"))+
    scale_alpha_manual(values = c(rep(1,3),0))+
    scale_size_manual(values = c(rep(2,3),1))+
    scale_y_continuous(limits = c(-1.6,1.6))+
    labs(y = "Weight", x = "MAG", color = "MAG\nannotation", size = "MAG\nannotation", alpha = "MAG\nannotation")+
    theme_classic()+
    theme(axis.text.x = element_blank(), axis.ticks.x = element_blank(),
          axis.text = element_text(color = "black", size = 12), legend.position = "right",
          axis.title = element_text(size = 13), legend.text = element_text(size = 11), legend.title = element_text(size = 12),
          strip.text.x = element_blank(), strip.background = element_blank())+
  facet_wrap(~ factor, scales = "free_x", nrow = 1)

feed1.figMG
```

```
# summary(mofa.anno.mbol.f1$value)
# table(mofa.anno.mbol.f1$anno.figure)
mofa.anno.mbol.f1$anno.figure <- factor(mofa.anno.mbol.f1$anno.figure, 
                                 levels = c("acyl.carnitines","amino.acids","bile.acids","hydroxysteroids",""))

feed1.figMB <-
ggplot(mofa.anno.mbol.f1, aes(rank, value))+
    geom_hline(yintercept = 0.2, color = "grey50", linetype = 2)+
    geom_hline(yintercept = -0.2, color = "grey50", linetype = 2)+
    geom_point(color = "grey60", size = 1, alpha = 0.3, shape = 16)+
    geom_point(aes(color = anno.figure, alpha = anno.figure, size = anno.figure), shape = 16)+
    scale_color_manual(values = c(RColorBrewer::brewer.pal(4,"Set1"),"grey60"))+
    scale_alpha_manual(values = c(rep(1,4),0))+
    scale_size_manual(values = c(rep(2,4),1))+
    scale_y_continuous(limits = c(-1,1.8))+
    expand_limits(x = c(-10, 510))+
    labs(y = "Weight", x = "Metabolite", color = "Metabolite\nannotation", size = "Metabolite\nannotation", alpha = "Metabolite\nannotation")+
    theme_classic()+
    theme(axis.text.x = element_blank(), axis.ticks.x = element_blank(),
          axis.text = element_text(color = "black", size = 12), legend.position = "right",
          axis.title = element_text(size = 13), legend.text = element_text(size = 11), legend.title = element_text(size = 12),
          strip.text.x = element_blank(), strip.background = element_blank())+
  facet_wrap(~ factor, scales = "free_x", nrow = 1)
## bunch of unannotated metabolites in positive end of factor7 -- look into!
## 2 fatty acids, 1 Prostaglandins (lipid-related fatty acyl), 1 Oligopeptides, 2 unannotated
feed1.figMB
```

```
# summary(mofa.anno.tx.f1$value)
# table(mofa.anno.tx.f1$anno.figure)

mofa.anno.tx.f1$anno.figure <- factor(mofa.anno.tx.f1$anno.figure, 
                                      levels = c("immune","lipid","apolipoprotein","signaling","taurine","heme","ECM",""))
feed1.figTX <-
ggplot(mofa.anno.tx.f1, aes(rank, value))+
    geom_hline(yintercept = 0.2, color = "grey50", linetype = 2)+
    geom_hline(yintercept = -0.2, color = "grey50", linetype = 2)+
    geom_point(color = "grey60", size = 1, alpha = 0.3, shape = 16)+
    geom_point(aes(color = anno.figure, alpha = anno.figure, size = anno.figure), shape = 16)+
    scale_color_manual(values = c(brewer.pal(8,"Set1")[c(1:5,8)],"gold","grey60"))+
    scale_alpha_manual(values = c(rep(1,7),0))+
    scale_size_manual(values = c(rep(2,7),1))+
    scale_y_continuous(limits = c(-1,1))+
    expand_limits(x = c(-10, 510))+
    labs(y = "Weight", x = "Transcript", color = "Transcript\nannotation", size = "Transcript\nannotation", alpha = "Transcript\nannotation")+
    theme_classic()+
    theme(axis.text.x = element_blank(), axis.ticks.x = element_blank(),
          axis.text = element_text(color = "black", size = 12), legend.position = "right",
          axis.title = element_text(size = 13), legend.text = element_text(size = 11), legend.title = element_text(size = 12),
          strip.text.x = element_blank(), strip.background = element_blank())+
  facet_wrap(~ factor, scales = "free_x", nrow = 1)

feed1.figTX
```

```
feed1.figSIZE <-
ggplot(mofa.f1.sample.weights[which(mofa.f1.sample.weights$factor %in% c("Factor1","Factor2","Factor3","Factor6","Factor7")),], 
       aes(Size.class, value))+
  geom_hline(yintercept = 0, color = "grey50", linetype = 2)+
  geom_boxplot(aes(fill = Size.class), alpha = 0.25, outlier.colour = NA)+
  geom_point(aes(fill = Size.class), shape = 21, color = "black", size = 3, position = position_jitterdodge(jitter.width = 0.5))+
  # geom_signif(comparisons = list(c("Large","Small")), map_signif_level = FALSE)+
  geom_signif(comparisons = list(c("Large","Small")), map_signif_level = function(p){ ifelse(p < 0.05,"*","ns")})+ 
  #wilcox test and MOFA corr match
  scale_color_manual(values = anno_colours$Size.class)+
  scale_fill_manual(values = anno_colours$Size.class)+
  scale_y_continuous(limits = c(-5,10))+
  labs(x = "Size class", y = "Factor value")+
  theme_classic()+
  theme(axis.text = element_text(color = "black", size = 12), legend.position = "none",
        axis.title = element_text(size = 13),
        strip.text.x = element_text(size = 13, face = "bold"), strip.background = element_rect(color = "white", fill = "white"))+
  facet_wrap(~ factor, nrow = 1)

feed1.figCESTODE <-
ggplot(mofa.f1.sample.weights[which(mofa.f1.sample.weights$factor %in% c("Factor1","Factor2","Factor3","Factor6","Factor7")),], 
       aes(Tapeworm.bin, value))+
  geom_hline(yintercept = 0, color = "grey50", linetype = 2)+
  geom_boxplot(aes(fill = Tapeworm.bin), alpha = 0.25, outlier.colour = NA)+
  geom_point(aes(fill = Tapeworm.bin), shape = 21, color = "black", size = 3, position = position_jitterdodge(jitter.width = 0.5))+
  # geom_signif(comparisons = list(c("FALSE","TRUE")), map_signif_level = FALSE)+
  geom_signif(comparisons = list(c("FALSE","TRUE")), map_signif_level = function(p){ ifelse(p < 0.05,"*","ns")})+ 
  #wilcox test and MOFA corr match
  scale_color_manual(values = anno_colours$Tapeworm.bin)+
  scale_fill_manual(values = anno_colours$Tapeworm.bin)+
  scale_y_continuous(limits = c(-5,10))+
  labs(x = "Cestode detected", y = "Factor value")+
  theme_classic()+
  theme(axis.text = element_text(color = "black", size = 12), legend.position = "none",
        axis.title = element_text(size = 13),
        strip.text.x = element_blank(), strip.background = element_blank())+
  facet_wrap(~ factor, nrow = 1)

feed1.figSIZE
```

```
feed1.figCESTODE
```

```
plot_grid(feed1.figSIZE,feed1.figCESTODE,feed1.figMG,feed1.figMB,feed1.figTX,
          align = "hv", axis = "lr", ncol = 1, labels = c("A","B","C","D","E"),
          rel_heights = c(1,1,0.8,0.8,0.8))
```

```
ggsave("figures/Supp_figure_mofa_FEED1_summary_results.png", units = "in", dpi = 800, width = 12, height = 13)
```

**Fig. S9.** MOFA results from the Feed1 model, for
Factors 1, 2, 3, 6 and 7. These factors from the Feed1 model are shown
here because they captured similar patterns in variation as in the full
combined model (Fig. 4). The factor numbers are not comparable between
models (for links between factors in each model, see Table S9).
(**A**) Factors 1, 6 and 7 were correlated with size class.
(**B**) Factors 3 and 6 were correlated with cestode
detection. (**C–E**) Feature weights for the metagenome
(**C**), metabolome (**D**) and transcriptome
(**E**) for Factors 1, 2, 3, 6 and 7. Features are ranked
according to their weight. The higher the absolute weight, the more
strongly associated a feature is with that factor. A positive weight
indicates the feature has higher levels in samples with positive factor
values, while a negative weight indicates the opposite. Features with
weights > 0.2 or < -0.2 are coloured by MAG species or genus (C)
or most frequent functional annotation (D-E), while features with less
frequent annotations, or those with weights outside the threshold range
(as indicated by the dashed lines) are shown in grey.

### Feed2 model

Repeat MOFA for Feed2 samples only.

```
samples.feed2 <- meta[which(meta$Feed.Type == "Feed2"),"Fish.ID"]

mofa.input.feed2 <- mofa.input[which(mofa.input$sample %in% samples.feed2),]
# table(mofa.input.feed2$view)

mofa.trained.feed2 <- run_mofa_default(
  df.input = mofa.input.feed2, 
  outfile = "output_MOFA/mofa_results_feed2", 
  metadata = meta,
  covar = c("Size.class","Tapeworm.bin","Mbol.batch"),
  view.distrib = c("gaussian","gaussian","bernoulli"),
  num_factors = 15
)
```

```
plot_variance_explained(mofa.trained.feed2$trained.obj, plot_total = T)[[2]]
```

```
plot_variance_explained(mofa.trained.feed2$trained.obj, x="view", y="factor")
```

```
mofa.trained.feed2$corr$Factor <- factor(mofa.trained.feed2$corr$Factor, 
                                   levels = unique(mofa.trained.feed2$corr$Factor)[c(1,8:15,2:7)])
ggplot(mofa.trained.feed2$corr, aes(Phenotype, Factor, fill = cor.logp))+
  geom_tile()+
  scale_fill_gradient(low = "white", high = "red")+
  theme_bw()+
  theme(title = element_text(size = 16), 
        axis.text = element_text(size = 14, colour = "black"),
        legend.text = element_text(size = 14, colour = "black"))
```

```
mofa.f2.sample.weights <- merge(
  mofa.trained.feed2$sample.weights, 
  meta[,c("Fish.ID","Feed.Type","Size.class","Tapeworm.bin","Mbol.batch")],
  by.x = "sample", by.y = "Fish.ID", all = F
)
```

```
# annotate features by functional categories (defined in annotate_mofa_f function)
mofa.anno.mgx.f2 <- annotate_mofa_f(mofa.trained.feed2$feature.weights, "MetaG",
                                    c("Factor1","Factor2","Factor3","Factor4","Factor5","Factor6"), threshold = 0.2)

mofa.anno.tx.f2 <- annotate_mofa_f(mofa.trained.feed2$feature.weights, "HostRNA",
                                   c("Factor1","Factor2","Factor3","Factor4","Factor5","Factor6"), threshold = 0.2)

mofa.anno.mbol.f2 <- annotate_mofa_f(mofa.trained.feed2$feature.weights, "mbol",
                                     c("Factor1","Factor2","Factor3","Factor4","Factor5","Factor6"), threshold = 0.2)

## after running this, realised that Factor3 doesn't correlate with fish phenotypes and is difficult to interpret
## so won't include in the figure.
```

#### Supp figure

Comparisons with combined model:

- Factor1 (metabolome, correlated with size class) is similar to
  Factor1 in combined model
- Factor2 (RNA, correlated with no fish phenotypes) is similar to
  Factor2 in combined model
- Factor4 (metagenome, correlated with tapeworm detection) is similar
  to Factor3 in combined model
- Factor5 (metagenome, RNA, correlated with no fish phenotypes) is
  somewhat similar to Factor7 in combined model
- Factor6 (RNA, correlated with size class and tapeworm detection) is
  somewhat similar to Factor2 and 7 in combined model

```
# order features by weight
mofa.anno.mgx.f2$rank <- paste(mofa.anno.mgx.f2$feature,mofa.anno.mgx.f2$factor, sep = "_")
mofa.anno.mgx.f2$rank <- factor(mofa.anno.mgx.f2$rank, 
                                levels = mofa.anno.mgx.f2[order(mofa.anno.mgx.f2$value, decreasing = F),"rank"])
mofa.anno.mbol.f2$rank <- paste(mofa.anno.mbol.f2$feature,mofa.anno.mbol.f2$factor, sep = "_")
mofa.anno.mbol.f2$rank <- factor(mofa.anno.mbol.f2$rank, 
                                 levels = mofa.anno.mbol.f2[order(mofa.anno.mbol.f2$value, decreasing = F),"rank"])
mofa.anno.tx.f2$rank <- paste(mofa.anno.tx.f2$feature,mofa.anno.tx.f2$factor, sep = "_")
mofa.anno.tx.f2$rank <- factor(mofa.anno.tx.f2$rank, 
                               levels = mofa.anno.tx.f2[order(mofa.anno.tx.f2$value, decreasing = F),"rank"])
```

```
# table(mofa.anno.mgx.f2$anno.figure)
# summary(mofa.anno.mgx.f2$value)

mofa.anno.mgx.f2$anno.figure <- factor(mofa.anno.mgx.f2$anno.figure, 
                                      levels = c("M.salmoninae","mycoplasma","Photobacterium","Aliivibrio",""))
feed2.figMG <-
ggplot(subset(mofa.anno.mgx.f2, factor != "Factor3"), aes(rank, value))+
    geom_hline(yintercept = 0.2, color = "grey50", linetype = 2)+
    geom_hline(yintercept = -0.2, color = "grey50", linetype = 2)+
    geom_point(color = "grey60", size = 1, alpha = 0.7, shape = 16)+
    geom_point(aes(color = anno.figure, alpha = anno.figure, size = anno.figure), shape = 16)+
    scale_color_manual(values = c(RColorBrewer::brewer.pal(4,"Set1"),"grey60"))+
    scale_alpha_manual(values = c(rep(1,4),0))+
    scale_size_manual(values = c(rep(2,4),1))+
    scale_y_continuous(limits = c(-1,1.3))+
    labs(y = "Weight", x = "MAG", color = "MAG\nannotation", size = "MAG\nannotation", alpha = "MAG\nannotation")+
    theme_classic()+
    theme(axis.text.x = element_blank(), axis.ticks.x = element_blank(),
          axis.text = element_text(color = "black", size = 12), legend.position = "right",
          axis.title = element_text(size = 13), legend.text = element_text(size = 11), legend.title = element_text(size = 12),
          strip.text.x = element_blank(), strip.background = element_blank())+
  facet_wrap(~ factor, scales = "free_x", nrow = 1)

feed2.figMG
```

```
# summary(mofa.anno.mbol.f2$value)
# table(mofa.anno.mbol.f2$anno.figure)

mofa.anno.mbol.f2$anno.figure <- factor(mofa.anno.mbol.f2$anno.figure, 
                                 levels = c("acyl.carnitines","amino.acids","bile.acids","hydroxysteroids",""))

feed2.figMB <-
ggplot(subset(mofa.anno.mbol.f2, factor != "Factor3"), aes(rank, value))+
    geom_hline(yintercept = 0.2, color = "grey50", linetype = 2)+
    geom_hline(yintercept = -0.2, color = "grey50", linetype = 2)+
    geom_point(color = "grey60", size = 1, alpha = 0.3, shape = 16)+
    geom_point(aes(color = anno.figure, alpha = anno.figure, size = anno.figure), shape = 16)+
    scale_color_manual(values = c(RColorBrewer::brewer.pal(4,"Set1"),"grey60"))+
    scale_alpha_manual(values = c(rep(1,4),0))+
    scale_size_manual(values = c(rep(2,4),1))+
    scale_y_continuous(limits = c(-1.4,1.4))+
    expand_limits(x = c(-10, 510))+
    labs(y = "Weight", x = "Metabolite", color = "Metabolite\nannotation", 
         size = "Metabolite\nannotation", alpha = "Metabolite\nannotation")+
    theme_classic()+
    theme(axis.text.x = element_blank(), axis.ticks.x = element_blank(),
          axis.text = element_text(color = "black", size = 12), legend.position = "right",
          axis.title = element_text(size = 13), legend.text = element_text(size = 11), legend.title = element_text(size = 12),
          strip.text.x = element_blank(), strip.background = element_blank())+
  facet_wrap(~ factor, scales = "free_x", nrow = 1)

feed2.figMB
```

```
# summary(mofa.anno.tx.f2$value)
# table(mofa.anno.tx.f2$anno.figure)

mofa.anno.tx.f2$anno.figure <- factor(mofa.anno.tx.f2$anno.figure, 
                                      levels = c("immune","lipid","apolipoprotein","signaling","taurine","heme","ECM",""))
feed2.figTX <-
ggplot(subset(mofa.anno.tx.f2, factor != "Factor3"), aes(rank, value))+
    geom_hline(yintercept = 0.2, color = "grey50", linetype = 2)+
    geom_hline(yintercept = -0.2, color = "grey50", linetype = 2)+
    geom_point(color = "grey60", size = 1, alpha = 0.3, shape = 16)+
    geom_point(aes(color = anno.figure, alpha = anno.figure, size = anno.figure), shape = 16)+
    scale_color_manual(values = c(brewer.pal(8,"Set1")[c(1:5,8)],"gold","grey60"))+
    scale_alpha_manual(values = c(rep(1,7),0))+
    scale_size_manual(values = c(rep(2,7),1))+
    scale_y_continuous(limits = c(-1.1,1.1))+
    expand_limits(x = c(-10, 510))+
    labs(y = "Weight", x = "Transcript", color = "Transcript\nannotation", size = "Transcript\nannotation", alpha = "Transcript\nannotation")+
    theme_classic()+
    theme(axis.text.x = element_blank(), axis.ticks.x = element_blank(),
          axis.text = element_text(color = "black", size = 12), legend.position = "right",
          axis.title = element_text(size = 13), legend.text = element_text(size = 11), legend.title = element_text(size = 12),
          strip.text.x = element_blank(), strip.background = element_blank())+
  facet_wrap(~ factor, scales = "free_x", nrow = 1)

feed2.figTX
```

```
feed2.figSIZE <-
ggplot(mofa.f2.sample.weights[which(mofa.f2.sample.weights$factor %in% c("Factor1","Factor2","Factor4","Factor5","Factor6")),], 
       aes(Size.class, value))+
  geom_hline(yintercept = 0, color = "grey50", linetype = 2)+
  geom_boxplot(aes(fill = Size.class), alpha = 0.25, outlier.colour = NA)+
  geom_point(aes(fill = Size.class), shape = 21, color = "black", size = 3, position = position_jitterdodge(jitter.width = 0.5))+
  # geom_signif(comparisons = list(c("Large","Small")), map_signif_level = FALSE)+
  geom_signif(comparisons = list(c("Large","Small")), map_signif_level = function(p){ ifelse(p < 0.05,"*","ns")})+ #wilcox test and MOFA corr match
  scale_color_manual(values = anno_colours$Size.class)+
  scale_fill_manual(values = anno_colours$Size.class)+
  scale_y_continuous(limits = c(-7.5,5))+
  labs(x = "Size class", y = "Factor value")+
  theme_classic()+
  theme(axis.text = element_text(color = "black", size = 12), legend.position = "none",
        axis.title = element_text(size = 13),
        strip.text.x = element_text(size = 13, face = "bold"), strip.background = element_rect(color = "white", fill = "white"))+
  facet_wrap(~ factor, nrow = 1)

feed2.figCESTODE <-
ggplot(mofa.f2.sample.weights[which(mofa.f2.sample.weights$factor %in% c("Factor1","Factor2","Factor4","Factor5","Factor6")),], 
       aes(Tapeworm.bin, value))+
  geom_hline(yintercept = 0, color = "grey50", linetype = 2)+
  geom_boxplot(aes(fill = Tapeworm.bin), alpha = 0.25, outlier.colour = NA)+
  geom_point(aes(fill = Tapeworm.bin), shape = 21, color = "black", size = 3, position = position_jitterdodge(jitter.width = 0.5))+
  # geom_signif(comparisons = list(c("FALSE","TRUE")), map_signif_level = FALSE)+
  geom_signif(comparisons = list(c("FALSE","TRUE")), map_signif_level = function(p){ ifelse(p < 0.05,"*","ns")})+ #wilcox test and MOFA corr match
  scale_color_manual(values = anno_colours$Tapeworm.bin)+
  scale_fill_manual(values = anno_colours$Tapeworm.bin)+
  scale_y_continuous(limits = c(-7.5,5))+
  labs(x = "Cestode detected", y = "Factor value")+
  theme_classic()+
  theme(axis.text = element_text(color = "black", size = 12), legend.position = "none",
        axis.title = element_text(size = 13),
        strip.text.x = element_blank(), strip.background = element_blank())+
  facet_wrap(~ factor, nrow = 1)

feed2.figSIZE
```

```
feed2.figCESTODE
```

```
plot_grid(feed2.figSIZE,feed2.figCESTODE,feed2.figMG,feed2.figMB,feed2.figTX,
          align = "hv", axis = "lr", ncol = 1, labels = c("A","B","C","D","E"),
          rel_heights = c(1,1,0.8,0.8,0.8))
```

```
ggsave("figures/Supp_figure_mofa_FEED2_summary_results.png", units = "in", dpi = 800, width = 12, height = 13)
```

**Fig. S10.** MOFA results from the Feed2 model, for
Factors 1, 2, 4–6. These factors from the Feed2 model are shown here
because they captured similar patterns in variation as in the full
combined model (Fig. 4). The factor numbers are not comparable between
models (for links between factors in each model, see Table S9).
(**A**) Factors 1, 5 and 6 were correlated with size class.
(**B**) Factors 4 and 6 were correlated with cestode
detection. (**C–E**) Feature weights for the metagenome
(**C**), metabolome (**D**) and transcriptome
(**E**) for Factors 1, 2, 4–6. Features are ranked
according to their weight. The higher the absolute weight, the more
strongly associated a feature is with that factor. A positive weight
indicates the feature has higher levels in samples with positive factor
values, while a negative weight indicates the opposite. Features with
weights > 0.2 or < -0.2 are coloured by MAG species or genus (C)
or most frequent functional annotation (D-E), while features with less
frequent annotations, or those with weights outside the threshold range
(as indicated by the dashed lines) are shown in grey.

### Figure 4

```
# compare combined model strongly-weighted features (absolute value > 0.2) to feed type models,
# to identify sets of features that are consistently associated with fish phenotypes
# (not worrying about direction of values (pos or neg), as it seems pretty consistent)

# cestode + myco: run1 F3 = feed1 F3 = feed2 F4
cestode.mags <- intersect(
  as.character(subset(mofa.trained.feed1$feature.weights, 
                      view == "MetaG" & factor == "Factor3" & abs(value) > 0.2)$feature),
  as.character(subset(mofa.trained.feed2$feature.weights, 
                      view == "MetaG" & factor == "Factor4" & abs(value) > 0.2)$feature)
)
cestode.mbolites <- intersect(
  as.character(subset(mofa.trained.feed1$feature.weights, 
                      view == "mbol" & factor == "Factor3" & abs(value) > 0.2)$feature),
  as.character(subset(mofa.trained.feed2$feature.weights, 
                      view == "mbol" & factor == "Factor4" & abs(value) > 0.2)$feature)
)
cestode.genes <- intersect(
  as.character(subset(mofa.trained.feed1$feature.weights, 
                      view == "HostRNA" & factor == "Factor3" & abs(value) > 0.2)$feature),
  as.character(subset(mofa.trained.feed2$feature.weights, 
                      view == "HostRNA" & factor == "Factor4" & abs(value) > 0.2)$feature)
)

# size (no microbiome): MBOL: run1 F1 = feed1 F2 = feed2 F1 ; RNA: run1 F2 = feed1 F1 = feed2 F2+F6
size.mbolites <- intersect(
  as.character(subset(mofa.trained.feed1$feature.weights, 
                      view == "mbol" & factor == "Factor2" & abs(value) > 0.2)$feature),
  as.character(subset(mofa.trained.feed2$feature.weights, 
                      view == "mbol" & factor == "Factor1" & abs(value) > 0.2)$feature)
)
size.genes <- intersect(
  as.character(subset(mofa.trained.feed1$feature.weights, 
                      view == "HostRNA" & factor == "Factor1" & abs(value) > 0.2)$feature),
  unique(c(as.character(subset(mofa.trained.feed2$feature.weights, 
                               view == "HostRNA" & factor == "Factor2" & abs(value) > 0.2)$feature),
           as.character(subset(mofa.trained.feed2$feature.weights, 
                               view == "HostRNA" & factor == "Factor6" & abs(value) > 0.2)$feature)))
)

# size + Photobacterium: RNA: run1 F2 = feed1 F6 & 7; MBOL: run1 F1 = feed1 F7
size.photo.genes <- unique(c(as.character(subset(mofa.trained.feed1$feature.weights, 
                                                 view == "HostRNA" & factor == "Factor6" & abs(value) > 0.2)$feature),
                             as.character(subset(mofa.trained.feed1$feature.weights, 
                                                 view == "HostRNA" & factor == "Factor7" & abs(value) > 0.2)$feature)))
size.photo.mbolites <- as.character(subset(mofa.trained.feed1$feature.weights, 
                                           view == "mbol" & factor == "Factor7" & abs(value) > 0.2)$feature)
size.photo.mags <- unique(as.character(
  subset(mofa.trained.feed1$feature.weights, 
         view == "MetaG" & factor %in% c("Factor6","Factor7") & abs(value) > 0.2)$feature))

# size + Aliivibrio: RNA: run1 F7 = feed2 F5
size.ali.genes <- as.character(subset(mofa.trained.feed2$feature.weights, 
                                      view == "HostRNA" & factor == "Factor5" & abs(value) > 0.2)$feature)
size.ali.mags <- as.character(subset(mofa.trained.feed2$feature.weights, 
                                     view == "MetaG" & factor == "Factor5" & abs(value) > 0.2)$feature)

# annotate on whether finding is robust
mofa.anno.tx$comment <- sapply(seq(1,nrow(mofa.anno.tx)), function(i){
  if(abs(mofa.anno.tx[i,"value"]) > 0.2){ 
    f <- mofa.anno.tx[i,"factor"]
    feature <- mofa.anno.tx[i,"feature"]
    if(f == "Factor3"){ 
      # RNA cestode
      return(ifelse(feature %in% cestode.genes,"TW.Robust",""))
    } else if(f == "Factor2"){
      a <- c()
      # RNA size (no microbes)
      if(feature %in% size.genes){
        a <- append(a,"Size.NoMG.Robust")
      }
      # RNA size + photo
      if(feature %in% size.photo.genes){
        a <- append(a,"Size.Photo.Robust")
      }
      return(paste(a, collapse = ";"))
    } else if(f == "Factor7"){
      a <- c()
      # RNA size + ali
      if(feature %in% size.ali.genes){
        a <- append(a,"Size.Ali.Robust")
      }
      # RNA size + photo
      if(feature %in% size.photo.genes){
        a <- append(a,"Size.Photo.Robust")
      }
      return(paste(a, collapse = ";"))
    } else {
      return("")
    }
  } else {
    return("")
  }
})

mofa.anno.mbol$comment <- sapply(seq(1,nrow(mofa.anno.mbol)), function(i){
  if(abs(mofa.anno.mbol[i,"value"]) > 0.2){
    f <- mofa.anno.mbol[i,"factor"]
    feature <- mofa.anno.mbol[i,"feature"]
    if(f == "Factor3"){ 
      # mbol cestode
      return(ifelse(feature %in% cestode.mbolites,"TW.Robust",""))
    } else if(f == "Factor1"){
      a <- c()
      # mbol size (no microbes)
      if(feature %in% size.mbolites){
        a <- append(a,"Size.NoMG.Robust")
      }
      # mbol size + photo
      if(feature %in% size.photo.mbolites){
        a <- append(a,"Size.Photo.Robust")
      }
      return(paste(a, collapse = ";"))
    } else {
      return("")
    }
  } else {
    return("")
  }
})

mofa.anno.mgx$comment <- sapply(seq(1,nrow(mofa.anno.mgx)), function(i){
  if(abs(mofa.anno.mgx[i,"value"]) > 0.2){
    f <- mofa.anno.mgx[i,"factor"]
    feature <- mofa.anno.mgx[i,"feature"]
    if(f == "Factor3"){
      # metag cestode
      return(ifelse(feature %in% cestode.mags,"TW.Robust",""))
    } else if(f == "Factor7"){
      a <- c()
      # metag aliivibrio
      if(feature %in% size.ali.mags){
        a <- append(a,"Ali")
      }
      # metag photobacterium
      if(feature %in% size.photo.mags){
        a <- append(a,"Photo")
      }
      return(paste(a, collapse = ";"))
    } else {
      return("")
    }
  } else {
    return("")
  }
})

mofa.anno.mgx$rank <- paste(mofa.anno.mgx$feature,mofa.anno.mgx$factor, sep = "_")
mofa.anno.mgx$rank <- factor(mofa.anno.mgx$rank, 
                             levels = mofa.anno.mgx[order(mofa.anno.mgx$value, decreasing = F),"rank"])
mofa.anno.mbol$rank <- paste(mofa.anno.mbol$feature,mofa.anno.mbol$factor, sep = "_")
mofa.anno.mbol$rank <- factor(mofa.anno.mbol$rank, 
                              levels = mofa.anno.mbol[order(mofa.anno.mbol$value, decreasing = F),"rank"])
mofa.anno.tx$rank <- paste(mofa.anno.tx$feature,mofa.anno.tx$factor, sep = "_")
mofa.anno.tx$rank <- factor(mofa.anno.tx$rank, 
                            levels = mofa.anno.tx[order(mofa.anno.tx$value, decreasing = F),"rank"])
```

```
# summary(mofa.anno.mgx$value)
# table(mofa.anno.mgx$anno.figure)
mofa.anno.mgx$anno.figure <- factor(mofa.anno.mgx$anno.figure, 
                                      levels = c("M.salmoninae","mycoplasma","Photobacterium","Aliivibrio",""))
mofa.anno.mgx$robust <- ifelse(mofa.anno.mgx$comment == "","No","Yes")

mofa.mgx.plot <-
ggplot(mofa.anno.mgx, aes(rank, value))+
  geom_hline(yintercept = 0.2, color = "grey50", linetype = 2)+
  geom_hline(yintercept = -0.2, color = "grey50", linetype = 2)+
  geom_point(color = "grey60", size = 1, alpha = 0.7)+
  geom_point(aes(color = anno.figure, alpha = anno.figure, size = anno.figure, shape = robust))+
  scale_color_manual(values = c(RColorBrewer::brewer.pal(4,"Set1"),"grey60"))+
  scale_alpha_manual(values = c(rep(1,4),0))+
  scale_size_manual(values = c(rep(2,4),1))+
  scale_shape_manual(values = c(16,17))+
  scale_y_continuous(limits = c(-1.5,1))+
  labs(y = "Weight", x = "MAG", color = "MAG\nannotation", size = "MAG\nannotation", alpha = "MAG\nannotation",
       shape = "Robust")+
  theme_classic()+
  theme(axis.text.x = element_blank(), axis.ticks.x = element_blank(),
        axis.text = element_text(color = "black", size = 12), legend.position = "right",
        axis.title = element_text(size = 13), legend.text = element_text(size = 11), legend.title = element_text(size = 12),
        legend.box = "horizontal",
        strip.text.x = element_blank(), strip.background = element_blank())+
  facet_wrap(~ factor, scales = "free_x", nrow = 1)

mofa.mgx.plot
```

```
# summary(mofa.anno.mbol$value)
# table(mofa.anno.mbol$anno.figure)
mofa.anno.mbol$anno.figure <- factor(mofa.anno.mbol$anno.figure, 
                                      levels = c("acyl.carnitines","amino.acids","bile.acids","hydroxysteroids",""))
mofa.anno.mbol$robust <- ifelse(mofa.anno.mbol$comment == "","No","Yes")

mofa.mbol.plot <-
ggplot(mofa.anno.mbol, aes(rank, value))+
  geom_hline(yintercept = 0.2, color = "grey50", linetype = 2)+
  geom_hline(yintercept = -0.2, color = "grey50", linetype = 2)+
  geom_point(color = "grey60", size = 1, alpha = 0.7)+
  geom_point(aes(color = anno.figure, alpha = anno.figure, size = anno.figure, shape = robust))+
  scale_color_manual(values = c(RColorBrewer::brewer.pal(4,"Set1"),"grey60"))+
  scale_alpha_manual(values = c(rep(1,4),0))+
  scale_size_manual(values = c(rep(2,4),1))+
  scale_shape_manual(values = c(16,17))+
  scale_y_continuous(limits = c(-1.5,1.5))+
  expand_limits(x = c(-10, 510))+
  labs(y = "Weight", x = "Metabolite", color = "Metabolite\nannotation", size = "Metabolite\nannotation", 
       alpha = "Metabolite\nannotation", shape = "Robust")+
  theme_classic()+
  theme(axis.text.x = element_blank(), axis.ticks.x = element_blank(),
        axis.text = element_text(color = "black", size = 12), legend.position = "right",
        axis.title = element_text(size = 13), legend.text = element_text(size = 11), legend.title = element_text(size = 12),
        legend.box = "horizontal",
        strip.text.x = element_blank(), strip.background = element_blank())+
  facet_wrap(~ factor, scales = "free_x", nrow = 1)

mofa.mbol.plot
```

```
# summary(mofa.anno.tx$value)
# table(mofa.anno.tx$anno.figure)
mofa.anno.tx$anno.figure <- factor(mofa.anno.tx$anno.figure, 
                                      levels = c("immune","lipid","apolipoprotein","signaling","taurine","heme","ECM",""))
mofa.anno.tx$robust <- ifelse(mofa.anno.tx$comment == "","No","Yes")

mofa.tx.plot <-
ggplot(mofa.anno.tx, aes(rank, value))+
  geom_hline(yintercept = 0.2, color = "grey50", linetype = 2)+
  geom_hline(yintercept = -0.2, color = "grey50", linetype = 2)+
  geom_point(color = "grey60", size = 1, alpha = 0.7)+
  geom_point(aes(color = anno.figure, alpha = anno.figure, size = anno.figure, shape = robust))+
  scale_color_manual(values = c(brewer.pal(8,"Set1")[c(1:5,8)],"gold","grey60"))+
  scale_alpha_manual(values = c(rep(1,7),0))+
  scale_size_manual(values = c(rep(2,7),1))+
  scale_shape_manual(values = c(16,17))+
  scale_y_continuous(limits = c(-1,1))+
  expand_limits(x = c(-10, 510))+
  labs(y = "Weight", x = "Transcript", color = "Transcript\nannotation", size = "Transcript\nannotation", alpha = "Transcript\nannotation",
       shape = "Robust")+
  theme_classic()+
  theme(axis.text.x = element_blank(), axis.ticks.x = element_blank(),
        axis.text = element_text(color = "black", size = 12), legend.position = "right",
        axis.title = element_text(size = 13), legend.text = element_text(size = 11), legend.title = element_text(size = 12),
        legend.box = "horizontal",
        strip.text.x = element_blank(), strip.background = element_blank())+
  facet_wrap(~ factor, scales = "free_x", nrow = 1)

mofa.tx.plot
```

```
mofa.size <-
ggplot(mofa.sample.weights[which(mofa.sample.weights$factor %in% c("Factor1","Factor2","Factor3","Factor7")),], 
       aes(Size.class, value))+
  geom_hline(yintercept = 0, color = "grey50", linetype = 2)+
  geom_boxplot(aes(fill = Size.class), alpha = 0.25, outlier.colour = NA)+
  geom_point(aes(fill = Size.class), shape = 21, color = "black", size = 3, 
             position = position_jitterdodge(jitter.width = 0.5))+
  geom_signif(comparisons = list(c("Large","Small")), map_signif_level = TRUE, annotations = c("*"))+ 
  #manual adding because wilcox test doesn't match MOFA correlation
  scale_color_manual(values = anno_colours$Size.class)+
  scale_fill_manual(values = anno_colours$Size.class)+
  scale_y_continuous(limits = c(-6.2,6))+
  labs(x = "Size class", y = "Factor value")+
  theme_classic()+
  theme(axis.text = element_text(color = "black", size = 12), legend.position = "none",
        axis.title = element_text(size = 13),
        strip.text.x = element_text(size = 13, face = "bold"), 
        strip.background = element_rect(color = "white", fill = "white"))+
  facet_wrap(~ factor, nrow = 1)

mofa.cestode <-
ggplot(mofa.sample.weights[which(mofa.sample.weights$factor %in% c("Factor1","Factor2","Factor3","Factor7")),], 
       aes(Tapeworm.bin, value))+
  geom_hline(yintercept = 0, color = "grey50", linetype = 2)+
  geom_boxplot(aes(fill = Tapeworm.bin), alpha = 0.25, outlier.colour = NA)+
  geom_point(aes(fill = Tapeworm.bin), shape = 21, color = "black", size = 3, 
             position = position_jitterdodge(jitter.width = 0.5))+
  geom_signif(comparisons = list(c("FALSE","TRUE")), map_signif_level = function(p){ ifelse(p < 0.05,"*","ns")})+ 
  #wilcox test and MOFA corr match
  scale_color_manual(values = anno_colours$Tapeworm.bin)+
  scale_fill_manual(values = anno_colours$Tapeworm.bin)+
  scale_y_continuous(limits = c(-6.2,6))+
  labs(x = "Cestode detected", y = "Factor value")+
  theme_classic()+
  theme(axis.text = element_text(color = "black", size = 12), legend.position = "none",
        axis.title = element_text(size = 13),
        strip.text.x = element_blank(), strip.background = element_blank())+
  facet_wrap(~ factor, nrow = 1)

mofa.feedtype <-
ggplot(mofa.sample.weights[which(mofa.sample.weights$factor %in% c("Factor1","Factor2","Factor3","Factor7")),], 
       aes(Feed.Type, value))+
  geom_hline(yintercept = 0, color = "grey50", linetype = 2)+
  geom_boxplot(aes(fill = Feed.Type), alpha = 0.25, outlier.colour = NA)+
  geom_point(aes(fill = Feed.Type), shape = 21, color = "black", size = 3, 
             position = position_jitterdodge(jitter.width = 0.5))+
  geom_signif(comparisons = list(c("Feed1","Feed2")), map_signif_level = function(p){ ifelse(p < 0.05,"*","ns")},
              test = "t.test")+ 
  #t test and MOFA corr match
  scale_color_manual(values = anno_colours$Feed.Type)+
  scale_fill_manual(values = anno_colours$Feed.Type)+
  scale_y_continuous(limits = c(-6.2,6))+
  labs(x = "Feed type", y = "Factor value")+
  theme_classic()+
  theme(axis.text = element_text(color = "black", size = 12), legend.position = "none",
        axis.title = element_text(size = 13),
        strip.text.x = element_blank(), strip.background = element_blank())+
  facet_wrap(~ factor, nrow = 1)

mofa.size
```

```
mofa.cestode
```

```
mofa.feedtype
```

```
plot_grid(mofa.size,mofa.cestode,mofa.feedtype,mofa.mgx.plot,mofa.mbol.plot,mofa.tx.plot,
          align = "hv", axis = "lr", ncol = 1, labels = c("A","B","C","D","E","F"),
          rel_heights = c(1,1,1,0.8,0.8,0.8))
```

```
ggsave("figures/Figure_mofa_summary_results_combined.png", units = "in", dpi = 800, width = 12, height = 16)
```

**Fig. 4.** Multi-omics factor analysis (MOFA) results
for Factors 1, 2, 3 and 7 in the full model (i.e. both feed types
combined). (**A**) All four factors were correlated with
size class. (**B**) Factor 3 and 7 were correlated with
cestode detection. (**C**) Factors 1, 3 and 7 were
correlated with feed type. In A–C, \* indicates adjusted p < 0.05, ns
indicates adjusted p ≥ 0.05. (**D–F**) Feature weights for
the metagenome (**D**), metabolome (**E**) and
transcriptome (**F**) for Factors 1, 2, 3 and 7. Features
are ranked according to their weight. The higher the absolute weight,
the more strongly associated a feature is with that factor. A positive
weight indicates the feature has higher levels in samples with positive
factor values, while a negative weight indicates the opposite. Features
with weights > 0.2 or < -0.2 are coloured by MAG species or genus
(D) or most frequent functional annotation (E-F), while features with
less frequent annotations, or those with weights outside the threshold
range (as indicated by the dashed lines) are shown in grey. Features
that were consistently found between the two feed type MOFA models to
have similar patterns as in the full model (i.e. found in factors with
similar fish phenotype correlations and similar metagenome composition
patterns) are labelled as ‘robust’.

```
# Summaries for main text
mofa.anno.mbol$direction <- ifelse(mofa.anno.mbol$value > 0.2,"pos",ifelse(mofa.anno.mbol$value < -0.2,"neg",NA))
# table(mofa.anno.mbol[,c("direction","anno.figure","factor")])

mofa.anno.tx$direction <- ifelse(mofa.anno.tx$value > 0.2,"pos",ifelse(mofa.anno.tx$value < -0.2,"neg",NA))
# table(mofa.anno.tx[,c("direction","anno.figure","factor")])

mofa.anno.mbol.f1$direction <- ifelse(mofa.anno.mbol.f1$value > 0.2,"pos",ifelse(mofa.anno.mbol.f1$value < -0.2,"neg",NA))
# table(mofa.anno.mbol.f1[,c("direction","anno.figure","factor")])

mofa.anno.tx.f1$direction <- ifelse(mofa.anno.tx.f1$value > 0.2,"pos",ifelse(mofa.anno.tx.f1$value < -0.2,"neg",NA))
# table(mofa.anno.tx.f1[,c("direction","anno.figure","factor")])

mofa.anno.mbol.f2$direction <- ifelse(mofa.anno.mbol.f2$value > 0.2,"pos",ifelse(mofa.anno.mbol.f2$value < -0.2,"neg",NA))
# table(mofa.anno.mbol.f2[,c("direction","anno.figure","factor")])

mofa.anno.tx.f2$direction <- ifelse(mofa.anno.tx.f2$value > 0.2,"pos",ifelse(mofa.anno.tx.f2$value < -0.2,"neg",NA))
# table(mofa.anno.tx.f2[,c("direction","anno.figure","factor")])

# # factor 1/2 and size
# sort(table(mofa.anno.mbol[which(mofa.anno.mbol$factor == "Factor1" & mofa.anno.mbol$direction == "pos"),c("R5.specific")]))
# mofa.anno.tx[which(mofa.anno.tx$factor == "Factor2" & mofa.anno.tx$direction == "pos"),c("gene.name")]
# View(mofa.anno.tx.f1[which(mofa.anno.tx.f1$factor == "Factor1" & mofa.anno.tx.f1$direction == "pos"),])
# View(mofa.anno.tx.f1[which(mofa.anno.mgx.f1$factor == "Factor6" & mofa.anno.tx.f1$direction == "pos"),])
# View(mofa.anno.tx.f2[which(mofa.anno.tx.f2$factor == "Factor5" & mofa.anno.tx.f2$direction == "pos"),])
```

### MOFA Supp data

```
# write summaries - only "significant" findings to cut down size

write.table(mofa.anno.mbol[which(abs(mofa.anno.mbol$value) > 0.2),
                           c("feature","value","factor","R1.superclass", "R2.class",
                             "R3.subclass", "R4.level5", "R5.specific", "top.anno",
                             "anno.figure","robust")],
            "tables/Supp_tabl_mofa_combined_mainFig_features_metabolome.txt", 
            sep = "\t", quote = F, row.names = F)

write.table(mofa.anno.mgx[which(abs(mofa.anno.mgx$value) > 0.2),c("feature","value","factor",
                                                                  "anno.figure","robust")],
            "tables/Supp_tabl_mofa_combined_mainFig_features_metagenome.txt", 
            sep = "\t", quote = F, row.names = F)

write.table(mofa.anno.tx[which(abs(mofa.anno.tx$value) > 0.2),c("feature","value","factor",
                                                                "gene.name",
                                                                "anno.figure","robust")],
            "tables/Supp_tabl_mofa_combined_mainFig_features_transcriptome.txt", 
            sep = "\t", quote = F, row.names = F)
```

# END
